# Temozolomide promotes glioblastoma stemness expression through senescence-associated reprogramming via HIF1α/HIF2α regulation

**DOI:** 10.1038/s41419-025-07617-w

**Published:** 2025-04-19

**Authors:** Pan Wang, Bin Liao, Sheng Gong, HaiYan Guo, Lu Zhao, Jie Liu, Nan Wu

**Affiliations:** 1 Department of Neurosurgery, Chongqing Research Center for Glioma Precision Medicine, Chongqing General Hospital, Chongqing University, Chongqing, China; 2 Chongqing Medical University, Chongqing, China

**Keywords:** CNS cancer, Cancer microenvironment, Cancer stem cells

## Abstract

A critical challenge in glioblastoma multiforme (GBM) treatment is that tumors recurring after temozolomide (TMZ) therapy become more malignant, exhibiting increased invasiveness and stemness compared to the primary tumor. However, the underlying mechanisms remain unclear. While the majority of GBM cells are eradicated by TMZ, a subset enters cell cycle arrest, adopts a senescence-associated secretory phenotype (SASP), and activates senescence-related signaling pathways. These cells eventually escape senescence, re-enter the cell cycle, and form aggregates exhibiting stem-like characteristics such as elevated stemness marker expression, enhanced colony formation, increased invasiveness, and resistance to chemotherapy. Furthermore, these aggregates promote the invasion and chemotherapy resistance of surrounding cells. Gene Set Enrichment Analysis (GSEA) and KEGG pathway analysis of miRNA and mRNA sequences revealed activation of hallmark hypoxia and HIF1 signaling pathways. The study demonstrated that HIF1α and HIF2α expression fluctuates during and after TMZ treatment. Knockout of HIF1α and HIF2α in GBM cells exposed to TMZ reduced the formation of senescent cells and stem-like aggregates. These findings challenge the efficacy of TMZ therapy by highlighting its role in inducing the process of cellular senescence, thereby contributing to the enhanced stemness and malignancy of recurrent GBM. The regulatory roles of HIF1α and HIF2α are emphasized, underscoring the necessity of preventing senescent cell formation and inhibiting HIF1α/HIF2α expression to improve therapeutic outcomes.

## Introduction

Temozolomide (TMZ) is a cornerstone in GBM treatment due to its potent cytotoxic effects, inducing apoptosis and necrosis in tumor cells. However, GBM frequently recurs after TMZ therapy, with recurrent tumors demonstrating greater invasiveness and resistance to chemotherapy, attributed to increased stemness [1, 2]. Although TMZ effectively eliminates most GBM cells, a small subset survives, serving as seeds for recurrence. Prior studies suggest that glioma stem cells (GSCs) are primary contributors to recurrent GBM due to their robust chemotherapy resistance [3]. Additionally, our previous research demonstrated that hypoxic microenvironments can induce the dedifferentiation of differentiated GBM cells into stem-like cells [4, 5]. This raises the question of whether differentiated GBM cells also undergo an increased expression of stemness in response to TMZ, akin to the hypoxic environment, and subsequently acquire stem-like characteristics.

Upon TMZ exposure, GBM cells typically undergo cell death through apoptosis or necrosis, while a subset enters a state of senescence. Cellular senescence, characterized by cell cycle arrest, halts proliferation, thereby allowing survival without division [6, 7]. Senescence activation by TMZ has been widely reported as a tumor suppressor mechanism, associated with features such as cellular hypertrophy, flattening, negative cell cycle regulator activation, hypermetabolism, SASP secretion, and chromatin reorganization regulated by P53, Wnt, DDR, and autophagy pathways [7–9]. However, recent evidence suggests that senescence is reversible through polyploidization and mitotic bypass [10], enabling senescent cells to escape and undergo atypical divisions, eventually re-emerging with enhanced stemness, invasiveness, and therapeutic resistance [11]. Consequently, senescent cells may give rise to new cells with stemness, and TMZ-induced senescence could drive an increased expression of stemness of GBM cells, leading to the formation of stem-like cells.

A study by Auffinger et al. [12] revealed that recurrent GBM post-TMZ therapy exhibits high expression of stemness markers and increased levels of HIF1α and HIF2α. These factors significantly upregulate transcription factors such as Sox2, Oct4, Klf4, and Nanog [13–15], which are known to reprogram differentiated tumor cells into stem-like phenotypes [16, 17]. Our prior findings demonstrated that HIF1α and HIF2α regulate the dedifferentiation of GBM cells in hypoxic conditions via Sox2 and Klf4 [5, 18]. These observations suggest that HIF1α and HIF2α may similarly mediate dedifferentiation under TMZ treatment, a hypothesis investigated in this study.

Overall, TMZ therapy delays but does not prevent GBM recurrence, with recurrent tumors exhibiting increased stemness and invasiveness. Previous research has largely overlooked the role of differentiated GBM cells in recurrence following TMZ therapy. This study examines the processes by which differentiated GBM cells enter senescence, subsequently acquire stem-like properties, and the molecular mechanisms underlying this transformation. Understanding these processes is crucial for developing therapeutic strategies to eradicate GBM.

## Materials and methods

### CD133-CD15- cell sorting and culturing

Primary GBM cells were isolated from tumor tissues during the operation, and the detailed steps were according to our previous article [5]. This primary GBM tissue was obtained in compliance with the principles of the Declaration of Helsinki. Ethical approval related to GBM tissues was approved by the ethics committee of Chongqing General Hospital (No. KY S2022-094-01), and informed consent was obtained from the patients. U87 and U118 cell lines were obtained from ATCC and cultured in DMEM/F12 medium (Hyclone, USA) supplemented with 10% fetal bovine serum (FBS, Gibco, USA) under standard conditions (21% O₂ and 5% CO₂ at 37 °C). After digestion with 0.25% trypsin, suspended cells in PBS containing 0.08% EDTA and 0.5% BSA (PBSE; 10⁸ cells/500 µl) were prepared. These cells were incubated with polyclonal rabbit anti-human CD133^+^ IgGs (Miltenyi Biotech, Germany) at 4 °C for 15 min, washed with PBS containing 1% BSA, and resuspended in PBSE (10⁸ cells/300 µl). Anti-rabbit IgG microbeads (Miltenyi Biotech, Germany) were added, and the cells were incubated at 10 °C for 15 min, followed by washing and resuspension in PBSE.

A cell separation column with a flow resistor was placed in a miniMACS magnet fixed to a magnet-activated cell sorting multistand, flushed with 500 µl PBSE, and loaded with the cell suspension. Unlabeled CD133^−^ cells passed through and were collected. These CD133^−^ cells were further processed to isolate CD15^−^ cells using the same procedure. The sorting process was repeated five times to ensure high purity of CD133^−^CD15^−^ cells. The resulting cells were cultured in DMEM/F12 medium containing 10% FBS and BMP2 to maintain their differentiated state. Cells were authenticated using STR profiling, and all the cells were found to have no mycoplasma contamination.

### TMZ treatment schedule, corresponding assays, and sequence analysis

CD133^−^CD15^−^ GBM cells cultured in DMEM/F12 medium supplemented with 10% FBS and BMP2 were treated with TMZ at concentrations of 25, 50, and 100 μM. Cell death was assessed using flow cytometry (FCM) following three and six days of treatment. A concentration of 50 μM was selected for subsequent experiments, with the culture medium replenished every four days at the same concentration. CD133^−^CD15^−^ cells were continuously exposed to 50 μM TMZ, and the proportions of β-Gal-positive and C_12_FDG-positive cells were quantified at days 2, 4, 6, and 8. Cell phenotypes were observed and documented at weeks 1, 2, and after a minimum of six weeks of TMZ exposure. Expression of senescence-associated secretory phenotype (SASP) markers—including IL1a, IL1b, IL6, IL8, CCL2, CDKN1A, CDKN2B, P53, CXCL3, ATM, COL5A1, EGF, FGF2, IGFBP3, MMP1, and uPA—was measured using RT-qPCR and ELISA on days 2, 4, 6, and 8 following TMZ treatment. Control and aggregation GBM cells were subjected to mRNA, miRNA, and protein sequence analyses by Sangon Biotech (Shanghai, China), which were uploaded in the NCBI Gene Expression Omnibus (GEO) database (www.ncbi.nlm.nih.gov/geo) under accession numbers GSE282387 and GSE282388. The mass spectrometry proteomics data have been deposited to the ProteomeXchange Consortium via the PRIDE [19] partner repository with the dataset identifier PXD058156. Bioinformatics analyses, including Gene Ontology (GO), KEGG pathway, Hallmark GSEA, and enrichment studies, were conducted to elucidate the characteristics of newly formed cells.

### Senescence detection and isolation of senescent cells

Cellular senescence was assessed using a β-Galactosidase Staining Kit (Beyotime, C0602), following the manufacturer’s protocol. GBM cells were collected at designated intervals, washed twice with PBS, and fixed with a solution of 2% formaldehyde and 0.2% glutaraldehyde in PBS. Cells were stained overnight at 37 °C with a solution containing 40 mmol/L citric acid/phosphate buffer (pH 6.0), 150 mmol/L NaCl, 2 mmol/L MgCl2, 5 mmol/L potassium ferrocyanide, 5 mmol/L potassium ferricyanide, and 0.1% x-Gal. Following washing, the cells were overlaid with 70% glycerin for imaging. Micrographs were captured using imaging software (Olympus) and a Zeiss Axiovert 35 microscope. Flow cytometry (FCM) was employed to detect C_12_FDG-positive cells. Cells were incubated with C_12_FDG in culture medium for 90 min at 37 °C, washed with PBS, trypsinized, and resuspended in PBS for analysis using a BD FACSCanto II flow cytometer. Differentiated GBM cells were cultured in TMZ (50 μM) for two weeks, resuspended in dissociation buffer containing culture medium, and sorted for C_12_FDG-positive and -negative cells using a BD FACS Aria sorter. The sorting procedure was repeated three times to ensure the purity of C_12_FDG-positive and -negative populations.

### Clonogenicity assays

Aggregation cells from CD133^−^CD15^−^ cells were digested with 0.25% trypsin, resuspended in DMEM/F12 medium, centrifuged, and cultured in stem cell medium (DMEM/F12 supplemented with EGF, FGF2, and B27), and cell morphology was observed, imaged, and recorded on day 5.

### Immunofluorescence detection

Aggregation cells collected at designated time points were washed with PBS, fixed in 4% paraformaldehyde for 30 min at 4 °C, washed three times with PBS, and permeabilized with 0.5% Triton X-100 (Sigma, USA) for 10 min. After additional washing, cells were blocked with 10% normal goat serum and incubated overnight at 4 °C with primary antibodies against CD133, CD15, Nestin, Sox2, Klf4, HIF1α, and HIF2α (Supplementary Table S13). Following three washes with PBS, cells were incubated with fluorophore-labeled secondary antibodies (CST, USA) at 37 °C for 2 h. Fluorescence images were obtained using a Zeiss LSM780 confocal microscope.

### Western blot detection

CD133^−^CD15^−^ cells treated with TMZ for one and two weeks, along with aggregation cells, were subjected to western blotting to evaluate the expression of stemness markers and transcription factors. Total protein was extracted using prechilled RIPA buffer (Beyotime Biotechnology, China). Protein samples were resolved via SDS-PAGE, transferred to PVDF membranes, and blocked with 5% non-fat milk. Membranes were incubated overnight at 4 °C with primary antibodies against CD133, CD15, Nestin, Sox2, and Klf4 (Supplementary Table S14). Subsequently, HRP-conjugated secondary antibodies (Beyotime Biotechnology, China) were applied at room temperature for 2 h. Protein bands were visualized using enhanced chemiluminescence.

### Real-time quantitative polymerase chain reaction

CD133^−^CD15^−^ cells treated with TMZ were used to assess RNA expression at specified intervals through RT-qPCR. Total RNA was extracted using TRIzol (Invitrogen, USA) and reverse-transcribed into cDNA with the MightyScript First Strand cDNA Synthesis Master Mix (Sangon Biotech, China). PCR amplification involved an initial denaturation at 94 °C for 5 min, followed by 40 cycles of denaturation at 94 °C for 30 s, annealing at 57 °C for 30 s, and extension at 72 °C for 30 s. Primer sequences are provided in Supplementary Table S15.

### Flow cytometry analysis

FCM was utilized to assess cell cycle progression, cell death, and stemness marker expression in CD133^−^CD15^−^ cells treated with TMZ (50 μM). For cell cycle analysis, cells were fixed at a density of 5 × 10^5^ cells/ml in PBS for 24 h at 4 °C, centrifuged, washed, and stained with propidium iodide (PI). For cell death analysis, Annexin V-FITC and PI staining were performed according to standard protocols. For stemness marker analysis, C_12_FDG-positive and -negative cells were cultured for 7, 14, and 21 days, then analyzed for the expression of CD133 and CD15 using specific antibodies (Miltenyi Biotech and R&D Systems). FACS analysis was performed with a BD Accuri C6 cytometer.

### Cell proliferation assay

Aggregation cells were labeled with RFP, mixed with control cells at a 1:1 ratio, and cultured in the presence of TMZ at concentrations of 0, 50, 100, and 200 µM for 1, 3, 5, and 7 days. At specified time points, FCM was employed to sort RFP-labeled aggregation cells, and the total number of RFP-labeled and non-labeled cells was counted. The proportion of aggregation and control cells at each time point was determined using the formula: (RFP-labeled cells or RFP-non-labeled cells) / (RFP-labeled cells + RFP-non-labeled cells).

### ELISA assays

CD133^−^CD15^−^ GBM cells at a density of 1 × 10^6^ cells per well were cultured in TMZ (50 µM) for 2, 4, 6, and 8 days. The culture medium was collected to measure the concentrations of IL1a, IL6, and IL8. Following removal of the conditioned media, cells were stored at −80 °C and subsequently counted. IL1a, IL6, and IL8 levels in the conditioned media were quantified using a commercial ELISA kit (R&D Systems) in accordance with the manufacturer’s instructions. Absorbance was measured at 450 nm using an ELISA plate reader (Varioskan Flash, Thermo Scientific, USA).

### Invasion assays

Invasion assays were conducted using 24-well plates containing transwell chambers. CD133^−^CD15^−^ GBM cells (1 × 10^5^) and aggregation cells (1 × 10^5^) in serum-free medium were introduced into the upper chambers, which were coated with a 40 μl Matrigel layer. DMEM/F12 supplemented with 10% FBS (600 μl) was added to the lower chambers. Cells were incubated for 48 h at 37 °C, after which the medium was aspirated. The cells were fixed with methanol, stained with 0.1% crystal violet solution for 15 min, and counted under a microscope, with the average taken from five random fields. For invasion detection, CD133^−^CD15^−^ GBM cells (1 × 10^5^) were added to the upper chambers, while CD133^−^CD15^−^ GBM cells (1 × 10^5^) or aggregation cells (1 × 10^5^) were placed in the lower Chambers.

RFP-labeled CD133^−^CD15^−^ cells (5 × 10^5^) were co-cultured with CD133^−^CD15^−^ cells (5 × 10^5^) or aggregation cells (5 × 10^5^). Similarly, RFP-labeled aggregation cells (5 × 10^5^) were co-cultured with CD133^−^CD15^−^ cells (5 × 10^5^) or aggregation cells (5 × 10^5^). FCM was used to sort RFP-labeled cells after 48 h of co-culturing. These sorted cells (1 × 10^5^) were introduced into the upper chamber to quantify the number of cells migrating through the chamber after an additional 48 h.

### In vivo study

U87-Luc cells were prepared as described previously. CD133^−^CD15^−^ control-Luc cells (1 × 10^6^) or aggregation cells-Luc (1 × 10^6^) were orthotopically implanted into BALB/c-nu mice (male, 4 ~ 6 weeks). In another experiment, CD133^−^CD15^−^ control-Luc cells (5 × 10^5^) were orthotopically implanted along with control cells (5 × 10^5^) or aggregation cells (5 × 10^5^). Mice were divided into groups that were either fed with TMZ or left untreated, and subsequent experiments were performed. Five mice per group were monitored for three weeks, during which tumor volume was measured via bioluminescence imaging. Mice were anesthetized and administered 200 μl (15 mg/ml) of luciferin intraperitoneally, and bioluminescence was detected using the NightOWL Macro Imaging system (LB983 NC320, Berthold Technologies, Germany). Tumor tissues from an additional five mice were collected after three weeks for western blot analysis of stemness marker expression. The remaining twenty mice were used for survival analysis.

All the animal procedures were approved by the ethics committee of ChongQing Medical University (No. IACUC-CQMU-2023-0167).

### HIF1α and HIF2α knockout assays

Plasmid constructs containing human HIF1α and HIF2α sgRNAs were designed using the CRISPR design program (http://crispr.mit.edu). The sgRNA oligonucleotide sequences are provided in Supplementary Table S16. Annealed sgRNAs were cloned into the lentiCRISPRv2 vector (Addgene, #52961, USA) and transfected into 293T cells along with packaging vectors psPAX2 (Addgene, #12260, USA) and pMD2.G (Addgene, #12259, USA). After 48 h, the viral supernatant was collected, filtered, and used to transduce U87 and U118 cells. Knockout of HIF1α and HIF2α was confirmed via western blot. To assess the effects of HIF1α or HIF2α knockout on dedifferentiation and senescence, cultured cells were analyzed under TMZ treatment and untreated conditions. Parameters such as stemness expression, cell death, cell cycle progression, and senescence markers (including β-Gal, C_12_FDG, and SASP expression) were evaluated following the methods described earlier.

### Statistical analysis

Statistical analyses were conducted using SPSS 22.0. All experiments were performed at least three times, and data are presented as the mean ± standard deviation (SD). Differences between two groups were analyzed using Student’s *t*-test, while comparisons among three or more groups were performed using one-way analysis of variance (ANOVA). Overall survival (OS) was analyzed using the log-rank test, and the Cox proportional hazards model was used to calculate hazard ratios with 95% confidence intervals. A *p*-value < 0.05 was considered statistically significant.

## Results

### Activation of stemness and senescence-associated factors from proteome analysis under TMZ treatment

**Fig. 1:**
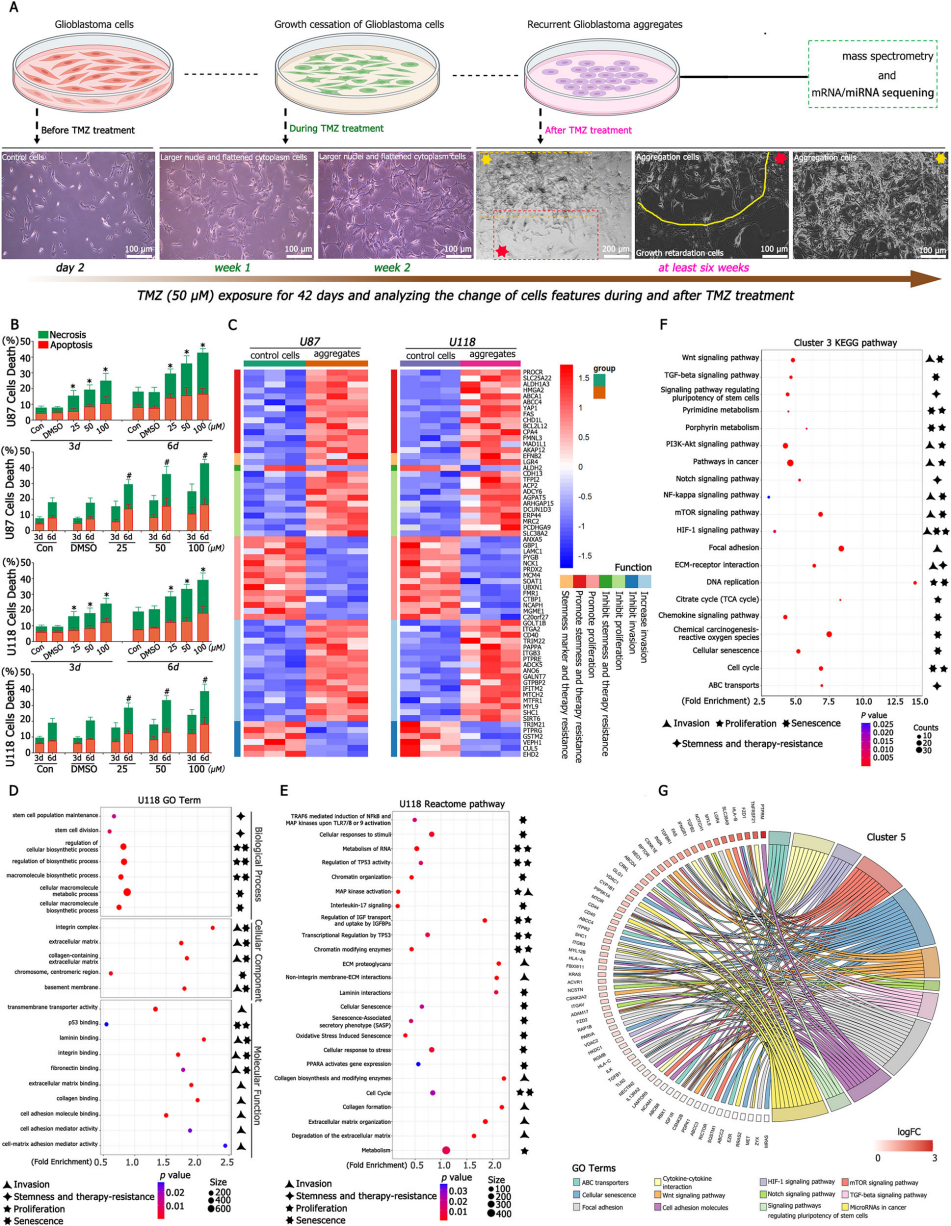
Activation of senescence- and stemness-associated pathways through proteomic analysis following TMZ treatment. **A** TMZ treatment process and associated changes in cellular morphology before, during, and after treatment. GBM cells displayed larger nuclei and a flattened cytoplasm, while new types of GBM aggregation cells formed after six to eight weeks of treatment. **B** GBM cells were cultured under varying TMZ concentrations (0, 25, 50, and 100 μM) for three to six days. Approximately 20% of GBM cells (including apoptotic and necrotic cells) died after three days of TMZ treatment at 50 μM, with significantly higher death rates compared to the control. The death rate increased proportionally with higher TMZ concentrations and longer exposure duration. **C** Mass spectrometry analysis indicated that aggregation cells highly expressed proteins associated with promoting stemness, invasion, and therapy resistance, whereas proteins that inhibited these processes or promoted proliferation were less expressed. **D** GO analysis revealed significant differences in terms related to stemness, invasion, chemotherapy resistance, and senescence between control and treated groups. Key terms included stem cell population maintenance, stem cell division, extracellular matrix, integrin complex, centromeric region, mismatched DNA binding, mismatch repair complex, integrin alpha2-beta1 complex, cell motility, and Notch signaling pathway. **E** Reactome pathway analysis highlighted the activation of pathways involved in extracellular matrix organization, transcriptional regulation by TP53, senescence-associated secretory phenotype (SASP), cellular senescence, cell cycle regulation, and diseases of mismatch repair (MMR). **F**, **G** KEGG pathway analysis identified activation of pathways associated with cellular senescence, focal adhesion, HIF1 signaling, mismatch repair, pluripotency of stem cells, cell cycle regulation, MAPK signaling, and others linked to senescence, stemness, invasion, and chemotherapy resistance. ^*^P < 0.05 were determined using one-way ANOVA and ^#^*P* < 0.05 were determined using Student’s *t*-test.

Approximately 20% and 40% of CD133^−^CD15^−^ U87 and U118 cells underwent apoptosis and necrosis, respectively, following treatment with TMZ (50 μM) for three and six days. The cell death rate increased in a dose- and time-dependent manner, with a pronounced increase in necrosis relative to apoptosis (Fig. 1A, B). Surviving cells exhibited larger cell and nuclear sizes, as well as flattened cytoplasm, but did not divide or proliferate under TMZ treatment (Fig. 1A). These enlarged cells demonstrated growth retardation during the six-week TMZ treatment period and up to six to eight weeks post-treatment, a subset of these cells subsequently re-entered the cell cycle, forming 6–9 aggregations per 1 × 10^7^ CD133^−^CD15^−^ cells (Fig. 1A). Sorted U87 aggregations cultured for an additional two weeks were processed for mass spectrometry, and differential expression proteins (DEPs) between control and aggregation cells were identified. These DEPs were localized to the cytoplasm (26.49%), nucleus (25.86%), plasma membrane (16.03%), mitochondria (12.82%), and extracellular compartment (10.92%), exhibiting similar trends in U118 cells (Fig. S1A). Among the DEPs, 159 overlapping upregulated and 272 overlapping downregulated proteins were identified in U87 and U118 cells (Fig. S1B). Further analysis revealed high expression of stemness-associated proteins (e.g., ABCA1, ABCC4, LGR4) and invasion-promoting proteins (e.g., ITGA2, ITGB3, SLC25A22), whereas proteins inhibiting invasion (e.g., CUL5, EDH2) were expressed at lower levels in aggregations (Fig. 1C, Table S1).

### Gene ontology analysis of differential proteins under TMZ treatment

Gene Ontology (GO) analysis classified the differential proteins into molecular function (MF), cellular component (CC), and biological process (BP) categories, revealing significant activation of processes such as stem cell population maintenance, stem cell division, extracellular matrix organization, integrin complex formation, cell adhesion molecule binding, mismatched DNA binding, mismatch repair complex activity, integrin alpha2-beta1 complex formation, cell motility, and Notch signaling pathway. These processes were associated with stemness, invasion, chemotherapy resistance, and senescence (Fig. 1D, Fig. S1C, and Table S2). Reactome pathway analysis corroborated these findings, highlighting the activation of pathways such as extracellular matrix organization, transcriptional regulation by TP53, SASP, cellular senescence, cell cycle regulation, and mismatch repair (MMR)-related diseases (Fig. 1E, Fig. S1D, and Table S3).

Differential proteins were categorized into six clusters based on expression trends (Fig. S2A). KEGG pathway analysis of these clusters revealed significant activation of pathways such as cellular senescence, focal adhesion, HIF1 signaling, mismatch repair, stem cell pluripotency, and cell cycle regulation, which are linked to senescence, stemness, invasion, and chemotherapy resistance (Fig. 1F, G and Table S4). Other clusters displayed similar pathways associated with these phenomena, as visualized in the UpSet plot (Fig. S2B).

### Activation of stemness and senescence-associated factors through mRNA sequencing

**Fig. 2:**
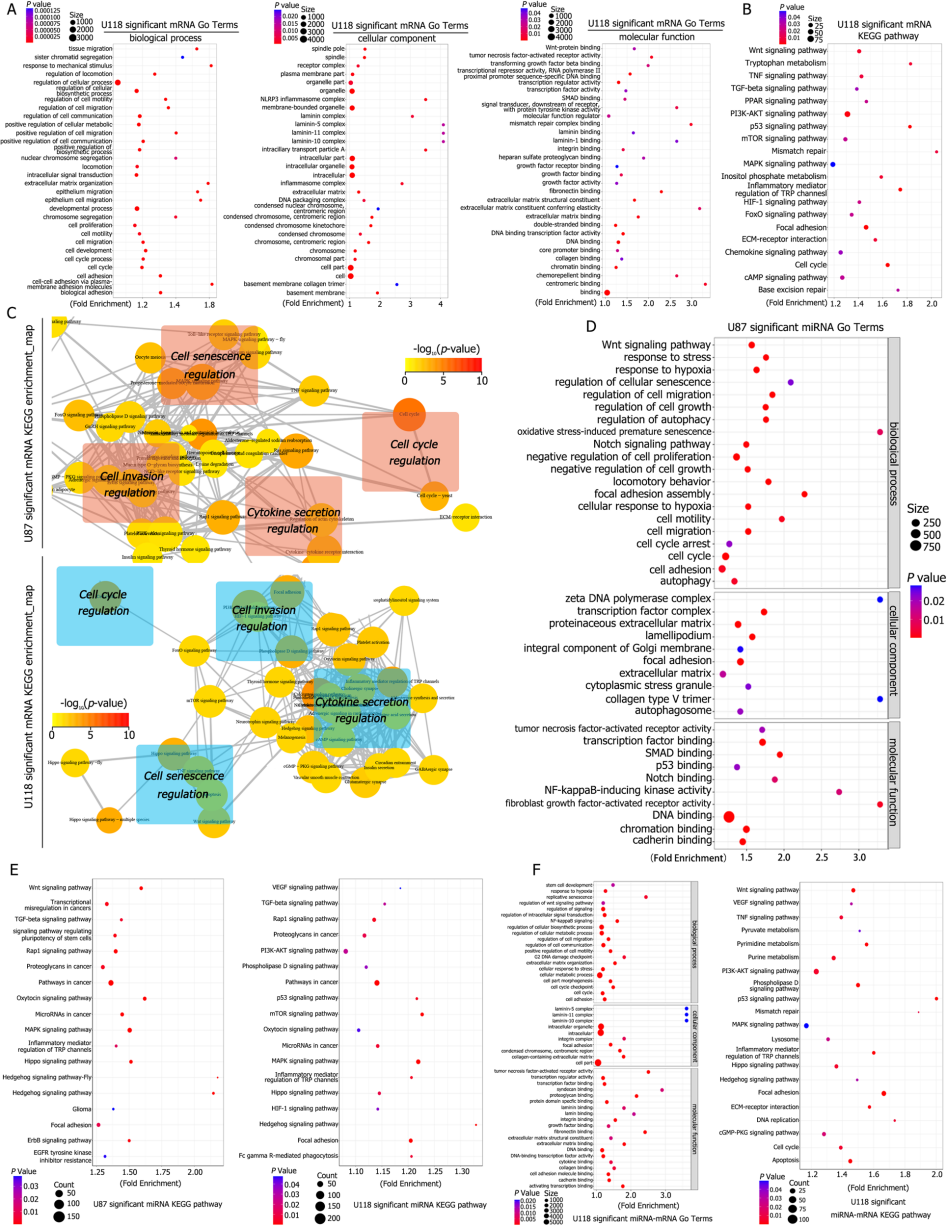
mRNA and miRNA sequencing analysis of control and aggregation cells. **A** GO analysis of differentially expressed mRNAs demonstrated activation of terms related to stemness, invasion, chemotherapy resistance, and senescence, including cell cycle regulation, positive regulation of locomotion, replicative senescence, cell migration, condensed chromosome, and mismatch repair complex binding. **B** KEGG pathway analysis of differentially expressed mRNAs revealed significant differences in pathways related to stemness, invasion, and senescence, such as cell cycle, focal adhesion, p53 signaling, mismatch repair, HIF1 signaling, ECM-receptor interaction, TNF signaling, and others. **C** KEGG pathway enrichment maps showed activation of pathways associated with cellular senescence, invasion, hypoxia response, and stemness, including focal adhesion, HIF1 signaling, PI3K-AKT signaling, and cGMP-PKG signaling. **D** GO analysis of differentially expressed miRNAs indicated activation of terms associated with stemness, invasion, hypoxia response, and senescence, including response to hypoxia, cell cycle regulation, Notch signaling, cell motility, autophagy, and regulation of cellular senescence. **E** KEGG pathway analysis of differentially expressed miRNAs revealed activation of pathways such as MAPK signaling, Wnt signaling, regulation of stem cell pluripotency, focal adhesion, HIF1 signaling, and mTOR signaling. **F** Combined analysis of differentially expressed mRNAs and miRNAs demonstrated significant differences in terms associated with replicative senescence, stem cell development, hypoxia response, cell cycle regulation, focal adhesion, collagen-containing extracellular matrix, integrin binding, and laminin binding. KEGG pathway analysis confirmed activation of processes related to stemness, invasion, hypoxia response, senescence, and cell communication.

To detect differential gene expression, mRNA sequencing was performed on aggregations formed after TMZ treatment, compared to control cells. GO analysis of the identified significant mRNAs revealed activation of MF, CC, and BP processes, including cell cycle regulation, positive regulation of locomotion, regulation of cell communication, replicative senescence, cell migration, condensed chromosome activity, mismatch repair complex binding, and Wnt-protein binding, all associated with stemness, invasion, chemotherapy resistance, and senescence (Fig. 2A and Table S5). KEGG pathway analysis further identified pathways such as cell cycle, focal adhesion, p53 signaling, mismatch repair, HIF1 signaling, ECM-receptor interaction, and TNF signaling as significantly different and relevant to these phenomena (Fig. 2B and Table S6).

Analysis of the 50 most enriched features highlighted correlations among chromosome segregation, cellular component organization, organelle organization, cell cycle processes, metabolic activity, and cell migration, demonstrating associations with the cell cycle, senescence, and invasion (Fig. S3). The comprehensive Gene Ontology enrichment map also indicated activation of processes such as condensed chromosome activity, cell cycle regulation, and intracellular organelle formation, which are implicated in senescence and invasion (Fig. S4).

KEGG pathway enrichment analysis underscored the activation of senescence-related pathways, including Wnt, TNF, p53, MAPK, phospholipase D, cell cycle, and mTOR signaling pathways. Invasion-related pathways such as focal adhesion, ECM-receptor interaction, and actin cytoskeleton regulation were also activated, alongside hypoxia, stemness, and signal transduction pathways, such as HIF1, PI3K-AKT, and cGMP-PKG signaling (Fig. 2C).

### Activation of stemness and senescence-associated factors through miRNA sequencing

MicroRNA (miRNA) sequence analysis was performed to identify differential miRNA expression in aggregations formed after TMZ treatment compared to control cells. GO analysis of significant miRNAs revealed activation of MF, CC, and BP processes related to stemness, invasion, hypoxia response, senescence, and cell communication. These processes included response to hypoxia, cell cycle regulation, Notch signaling, cell motility, autophagy, regulation of cellular senescence, replicative senescence, focal adhesion, and intracellular signal transduction (Fig. 2D and Table S7). KEGG pathway analysis demonstrated activation of pathways such as MAPK signaling, Wnt signaling, stem cell pluripotency, focal adhesion, HIF1 signaling, and mTOR signaling, all associated with the aforementioned phenomena (Fig. 2E and Table S8).

Integrating differential mRNAs and miRNAs for GO term analysis revealed significant differences in replicative senescence, stem cell development, hypoxia response, cell cycle regulation, focal adhesion, collagen-containing extracellular matrix formation, and binding activities involving cell adhesion molecules, integrins, and laminins (Fig. 2F and Table S9). KEGG pathway analysis showed consistent results, identifying significant processes such as ECM-receptor interaction, p53 signaling, Wnt signaling, DNA replication, mismatch repair, stem cell pluripotency, cytokine-cytokine receptor interaction, and NF-kappa B signaling (Fig. 2F and Table S10).

The network analysis of intersecting KEGG enrichment pathways between mRNAs and miRNAs revealed activation of focal adhesion and ECM-receptor interaction due to the upregulation of collagen and laminin genes, which are associated with cellular senescence and invasion. The phospholipase D signaling pathway was activated through ADCY family proteins, indicating hypermetabolism and senescence. Additionally, Wnt and p53 signaling pathways, implicated in regulating senescence, stemness, cell cycle progression, and invasion, were activated by differential expression of TP53, CCND1, CCND2, FAS, and ATM (Fig. S5).

### Aggregations formed after TMZ treatment exhibited enhanced stemness and invasiveness

**Fig. 3:**
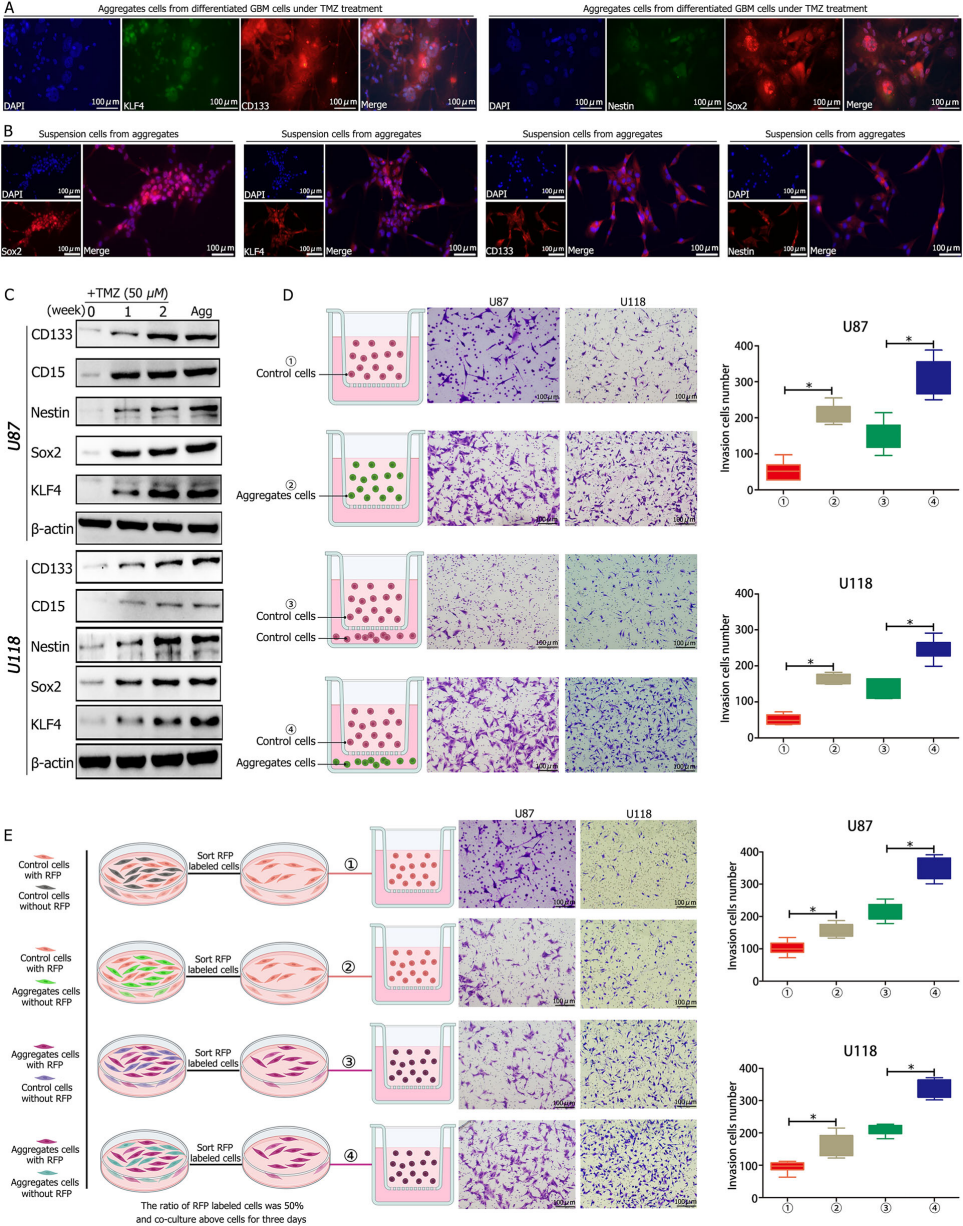
Characteristics of newly formed aggregation cells, highlighting stemness and invasion capabilities. **A** Immunofluorescence analysis showed high expression levels of stemness markers CD133, Nestin, and transcription factors Sox2 and Klf4 in aggregation cells. **B** Elevated expression levels of CD133, Nestin, Sox2, and Klf4 were observed after two weeks of culturing aggregation cells. **C** Western blot analysis confirmed increased expression of stemness markers CD133, CD15, Nestin, Sox2, and Klf4 after one to two weeks of TMZ treatment, with higher expression levels in aggregation cells. **D** Aggregation cells (group ②) demonstrated greater invasive capacity compared to controls (group ①). Co-culturing control and aggregation cells showed a higher number of migrating cells when control cells were co-cultured with aggregation cells (group ④) than with control cells (group ③). **E** RFP-labeled aggregation cells co-cultured with aggregation cells exhibited the highest invasive capacity (group ④) compared to other groups, in the order: group ④ > group ③ (RFP-aggregation cells co-cultured with control cells) > group ② (RFP-control cells co-cultured with aggregation cells) > group ① (RFP-control cells co-cultured with control cells). ^*^P < 0.05 was determined using Student’s *t*-test.

The expression of stemness markers, including CD133, CD15, Nestin, and transcription factors Sox2 and Klf4, was assessed in cells treated with TMZ. Immunofluorescence analysis demonstrated strong expression of CD133, Nestin, Sox2, and Klf4 in aggregations (Fig. 3A) and in suspension cells cultured for an additional two weeks from aggregations (Fig. 3B). Flow cytometric analysis indicated higher levels of CD133 and CD15 in the newly formed aggregations (Fig. S6A). Cells in aggregations were cultured in stem cell medium (DMEM/F12 supplemented with EGF, FGF2, and B27), growing in suspension (Fig. S6B).

**Fig. 4:**
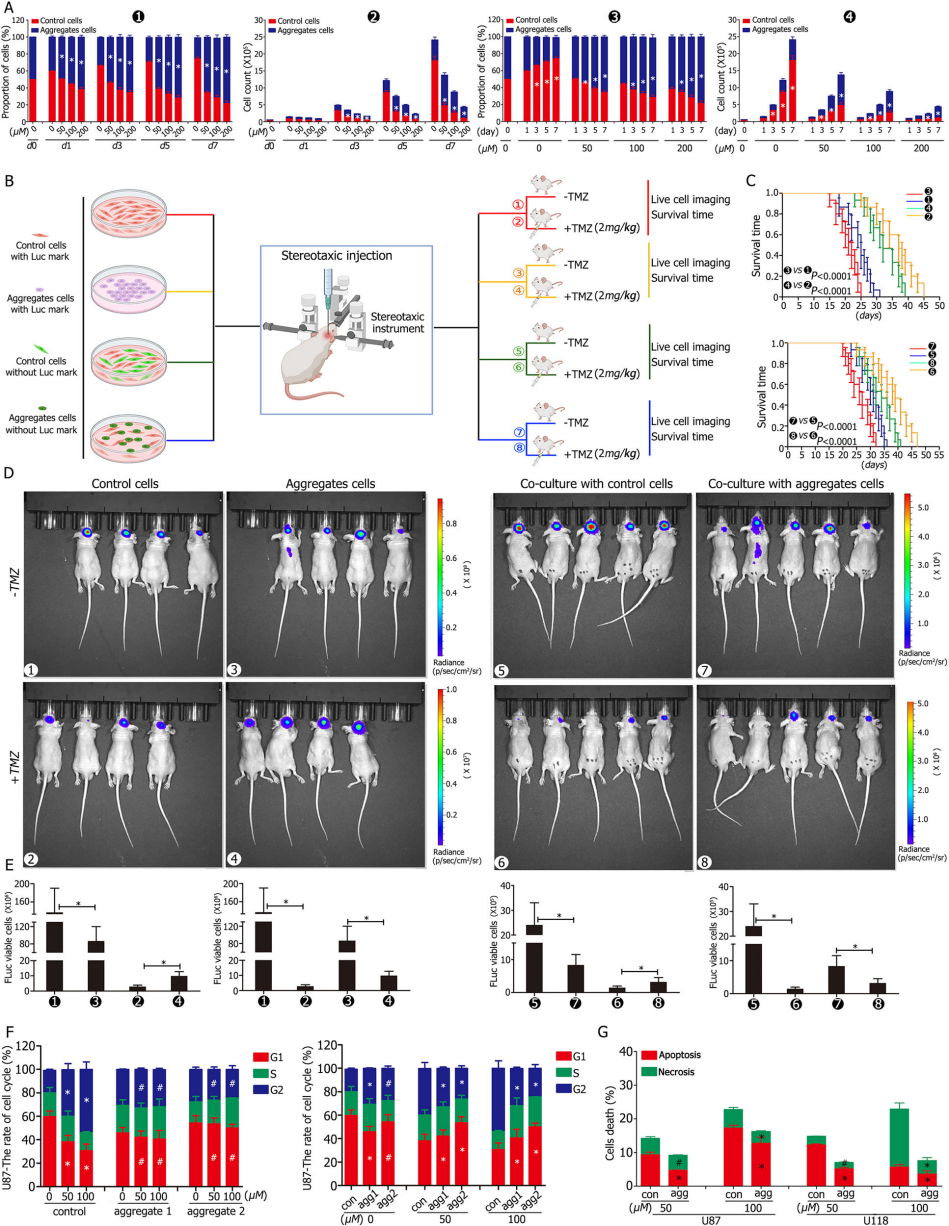
Aggregation cells exhibit lower proliferation rates but higher chemotherapy resistance. **A** Control and aggregation cells were co-cultured at a 1:1 ratio with or without TMZ for one week to assess proliferation. Without TMZ treatment, the proportion of U87 aggregation cells decreased from ~40% to ~30% over seven days (③), with cell counts of 6.12 × 10^5^ and 18.03 × 10^5^ for control cells (④). With TMZ treatment, the proportion of aggregation cells increased with longer exposure times (③) or higher TMZ concentrations (①), showing similar trends in cell counts (② and ④). **B** Schematic of in vivo experiments involving orthotopic implantation of control or aggregation cells with or without TMZ intraperitoneal injection. **C**–**E** Without TMZ, control cells (group ①, median survival: 25 days) formed larger tumors but resulted in longer survival compared to aggregation cells (group ③, median survival: 22 days). TMZ treatment reduced tumor volume and extended survival in both groups, with longer survival in control cells (group ②, median survival: 37 days) than aggregation cells (group ④, median survival: 32 days). Mixing control and aggregation cells (1:1 ratio) and orthotopically implanting them showed similar trends, with median survival of 31 vs. 27 days (groups ⑤ vs. ⑦) without TMZ and 39 vs. 34 days (groups ⑥ vs. ⑧) with TMZ. **F** Cell cycle analysis revealed that control cells predominantly occupied the G1 phase, while aggregation cells were in the G2 + S state. TMZ (50 μM) arrested control cells in the G2 phase, with an increased G2 proportion at 100 μM. Aggregation cells displayed no significant change in the cell cycle proportion with increasing TMZ concentration. **G** Aggregation cells exhibited lower apoptosis and necrosis rates than control cells under TMZ concentrations of 50 and 100 μM. ^*^P < 0.05 was determined using one-way ANOVA or Student’s *t*-test, and ^#^*P* > 0.05 was determined using Student’s *t*-test. Overall survival was analyzed using the log-rank test.

Moreover, CD133^−^CD15^−^ cells cultured in TMZ for two weeks exhibited increased expression of CD133, Nestin, Sox2, and Klf4 (Fig. S7A). Western blot analysis revealed no expression of CD133, CD15, Nestin, Sox2, or Klf4 in control cells, while their expression levels increased in a time-dependent manner following TMZ exposure. The newly formed aggregations also showed high expression of these markers (Fig. 3C, Fig. S8A). In vivo, CD133^−^CD15^−^ cells and aggregation cells were orthotopically implanted into mice treated with or without TMZ for three weeks (Fig. 4B, D). Western blot analysis of tumor tissues revealed elevated expression of CD133, CD15, Nestin, Sox2, and Klf4 in mice implanted with CD133^−^CD15^−^ cells treated with TMZ compared to controls without TMZ treatment. Mice implanted with aggregation cells also exhibited high expression levels of these markers (Fig. S7B).

Sequence data identified significant activation of pathways associated with invasion and cell communication (Figs. 1E, F, 2B, E, F). Aggregation-derived stem-like cells demonstrated greater invasive capabilities than control cells (Fig. 3D ①, ②, Fig. S8B ①, ②). Co-culturing experiments revealed an increased number of cells passing through the transwell when control cells were co-cultured with aggregation stem-like cells (Fig. 3D ③, ④, Fig. S8B ③, ④). Mixed culture experiments involving control cells, RFP-labeled control cells, aggregation stem-like cells, and RFP-labeled aggregation stem-like cells (Fig. 3E, Fig. S8C) indicated that RFP-aggregation stem-like cells co-cultured with aggregation stem-like cells exhibited the highest invasiveness (group ④), followed by RFP-aggregation stem-like cells co-cultured with control cells (group ③), RFP-control cells co-cultured with aggregation stem-like cells (group ②), and RFP-control cells co-cultured with control cells (group ①).

### Aggregation stem-like cells exhibit reduced proliferation but increased chemotherapy resistance

U87 control and aggregation stem-like cells were mixed at a 1:1 ratio and cultured with varying TMZ concentrations (0 μM, 50 μM, 100 μM, and 200 μM) for one week to compare their proliferation capacities. In the absence of TMZ treatment, aggregation stem-like cells constituted approximately 40% of the total cell population after one day of culture (Fig. 4A①, ③). This proportion decreased significantly to 25% after seven days, with aggregation stem-like cells numbering approximately 6.12 × 10^5^ and control cells numbering 18.03 × 10^5^ (Fig. 4A③-④). However, in the presence of TMZ, aggregation stem-like cells exhibited an increasing proportion of the cell population as TMZ exposure time and concentration increased. After three days of exposure to 50 μM TMZ, the proportion of aggregation stem-like cells surpassed that of control cells. This trend became more pronounced with higher TMZ concentrations and longer exposure times (Fig. 4A①, ③). By day seven under 200 μM TMZ, aggregation stem-like cells constituted nearly 80% of the population (3.40 × 10^5^), while control cells represented less than 20% (0.96 × 10^5^) (Fig. 4A①,②,③,④). Similar trends were observed in U118 and primary GBM cells (Fig. S9).

In vivo, control or aggregation stem-like cells were orthotopically implanted into mice with or without intraperitoneal TMZ injections (Fig. 4B①–④). Without TMZ treatment, tumor volumes were larger (Fig. 4D, E), but survival times were longer in the control group (group ①, median survival: 25 days) compared to the aggregation stem-like cell group (group ③, median survival: 22 days) (Fig. 4C). Following TMZ treatment, tumor volumes were reduced (Fig. 4D, E), and survival times increased for control cells (group ②, median survival: 37 days) compared to aggregation stem-like cells (group ④, median survival: 32 days) (Fig. 4C).

When cells were mixed in a 1:1 ratio with Luc-marked control cells and implanted orthotopically with or without TMZ treatment (Fig. 4B⑤–⑧), results indicated that, without TMZ, the tumor volume was larger in the control group (group ⑤) but with longer survival times than the group mixed with aggregation stem-like cells (group ⑦, median survival: 31 days *vs* 27 days, respectively). Following TMZ treatment, tumor volumes decreased, and survival times were longer in the control group (group ⑥) than in the group with aggregation stem-like cells (group ⑧, median survival: 39 days *vs* 34 days, respectively) (Fig. 4C–E).

Cell cycle analysis revealed that control cells had a higher proportion of cells in the G1 phase compared to aggregation stem-like cells. Upon exposure to 50 μM TMZ, control cells were arrested in the G2 phase, with this proportion increasing at 100 μM TMZ. In contrast, aggregation stem-like cells preferentially entered the S phase, and the proportion of cells in this phase did not significantly change with increasing TMZ concentrations (Fig. 4F, Fig. S10A). Finally, when exposed to TMZ (50 μM and 100 μM) for 48 h, aggregation stem-like cells exhibited lower apoptosis rates compared to controls under 50 μM TMZ exposure. At 100 μM TMZ, aggregation stem-like cells showed lower rates of both apoptosis and necrosis than control cells (Fig. 4G, Fig. S10B).

### Aggregation stem-like cells were formed through cellular senescence under TMZ treatment

**Fig. 5:**
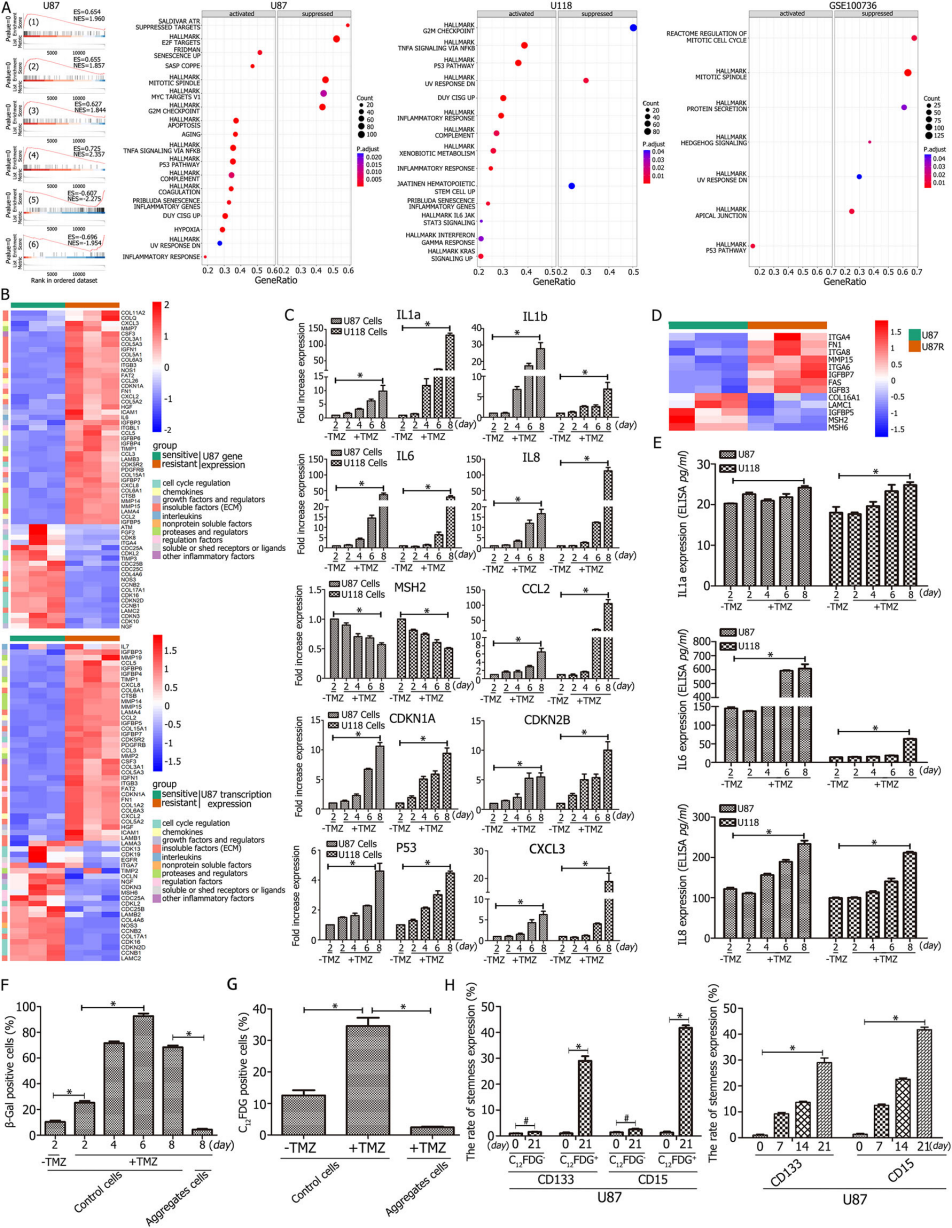
Aggregation cells are formed through cellular senescence under TMZ treatment. **A** GSEA analysis indicated upregulation of senescence-associated hallmarks and inhibition of growth-promoting pathways. **B** The heatmap of U87 overlapping differentially expressed genes (DEGs) with SASP at the mRNA level revealed upregulation of most DEGs, including IL6, IL7, CXCL3, CXCL2, ICAM1, CCL2, CCL3, MMP7, and TIMP1. Conversely, senescence-inhibiting genes such as CDC25B, CDC25C, CDC25A, CDKN2D, MSH6, and MSH5 were downregulated. **C** RT-qPCR analysis demonstrated significant time-dependent increases in the expression of senescence-promoting genes, including IL1a, IL1b, IL6, IL8, CCL2, CDKN1A, CDKN2B, P53, and CXCL3, while the expression of the senescence-inhibiting gene MSH2 decreased significantly. **D** Mass spectrometry revealed high expression of senescence-promoting SASP proteins, including ITGA4, MMP15, FN1, IGFB3, and FAS, with a concomitant decrease in senescence-suppressing SASP proteins, MSH2 and MSH6. **E** ELISA confirmed increased expression of IL1a, IL6, and IL8 in a time-dependent manner following TMZ treatment. **F** SA-β-Gal staining revealed a significant time-dependent increase in β-Gal-positive cells, and the rate of β-Gal positivity was lower in aggregation cells compared to control cells under TMZ treatment. **G** C_12_FDG expression increased approximately threefold after one week of TMZ treatment in CD133^−^CD15^−^ GBM cells, with lower levels of expression in aggregation cells under equivalent TMZ concentrations. **H** C_12_FDG-negative and -positive cells were cultured under TMZ for 21 days, showing significantly higher levels of CD133 and CD15 in C_12_FDG-positive cells over time, while CD133 and CD15 expression was not significant difference in C_12_FDG-negative cells. ^*^P < 0.05 and ^#^*P* > 0.05 were determined using Student’s *t*-test.

Sequence analysis revealed the activation of senescence-associated pathways, including cellular senescence, mismatch repair, cell cycle regulation, TNF signaling, and p53 signaling (Fig. 1E–G, Fig. 2B, E, F). GSEA analysis of mRNA sequences demonstrated the upregulation of senescence-related hallmarks such as aging, fridman senescence-up, senescence inflammatory genes, SASP-Coppe, the p53 pathway, and TNFA signaling via NF-kappa B, while growth-promoting pathways, including the G2M checkpoint, ATR-suppressed targets, and mitotic spindle formation, were inhibited (Fig. 5A, Fig. S12B, Table S11).

The senescence-associated secretory phenotype (SASP), a hallmark of cellular senescence, was highly prominent. Proteomic, mRNA, and miRNA analyses showed significant differences in senescence-associated genes between control and aggregation stem-like cells. At the mRNA level, there were 62, 59, 105, 104, and 41 overlapping SASP-associated genes in U87 DEGs, U87 transcript DEGs, U118 DEGs, U118 transcript DEGs, and GSE 100736 DEGs, respectively (Fig. S11A, Table S12). Additionally, 13 and 35 overlapping genes were identified in U87 DEGs and U118 DEGs at the proteome level (Fig. S11B, Table S12), while 57 and 108 overlapping target genes were detected at the miRNA level (Fig. S11C, Table S12).

Heatmap analysis of overlapping DEGs with SASP at the mRNA level showed significant upregulation of genes such as IL6, IL7, CXCL3, CXCL2, ICAM1, CCL2, CCL3, MMP7, and TIMP1, while senescence-inhibiting genes including CDC25B, CDC25 C, CDC25A, CDKN2D, MSH6, MSH5, and MSH2 were downregulated (Fig. 5B, Fig. S12A). RT-qPCR analysis confirmed time-dependent increases in SASP-related genes such as IL1a, IL1b, IL6, IL8, CCL2, CDKN1A, CDKN2B, P53, CXCL3, ATM, COL5A1, EGF, FGF2, IGFBP3, MMP1, and uPA, while MSH2 expression decreased significantly (Fig. 5C, Fig. S12C).

SASP-associated protein detection revealed high expression levels of ITGA4, MMP15, FN1, IGFBP3, and FAS, alongside decreased levels of MSH2 and MSH6 (Fig. 5D). ELISA results indicated a time-dependent increase in IL1a, IL6, and IL8 expression following TMZ treatment (Fig. 5E, Fig. S14A). Morphological analysis showed that surviving GBM cells under TMZ treatment became hypertrophic and flattened, with notable growth retardation (Fig. 1A, Fig. S13A). These cells were labeled by calcein and exhibited minimal PI expression (Fig. S13B). SA-β-Gal activity increased over time with TMZ exposure (Fig. 5F, Fig. S14B), while C_12_FDG expression approximately at least tripled after one week of TMZ treatment (Fig. 5G, Fig. S14C).

Exposure of aggregate cells to TMZ (50 μM) for eight days resulted in significantly lower SA-β-Gal and C_12_FDG expression compared to control cells treated with the same dose of TMZ (Fig. 5F, G, Fig. S14B-C). Further investigations into stemness expression revealed that C_12_FDG-positive cells exhibited a time-dependent increase in CD133 and CD15 levels, with approximately 30- and 40-fold increases, respectively, by day 21 compared to day 0. Conversely, C_12_FDG-negative cells showed no significant difference in expression of CD133 and CD15 over the same period (Fig. 5H, Fig. S14D).

### Cellular senescence and the formation of aggregation stem-like cells under TMZ treatment was regulated via HIF1α/HIF2α

**Fig. 6:**
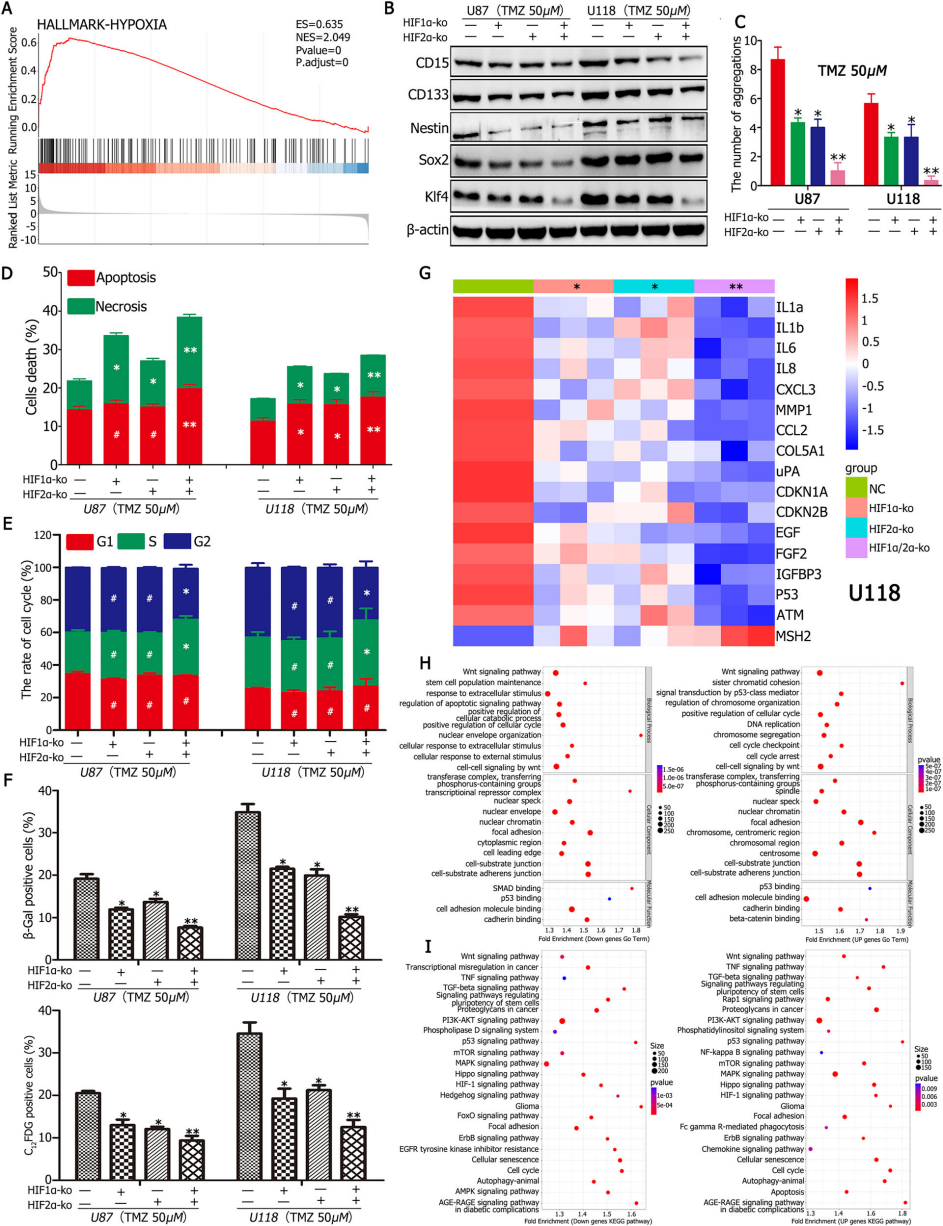
Regulation of senescence and aggregation stem-like cell formation by HIF1α/HIF2α under TMZ treatment. **A** GSEA analysis demonstrated significant upregulation of hypoxia hallmark pathways in newly formed aggregation cells. **B** HIF1α and HIF2α knockout CD133^−^CD15^−^ cells cultured under TMZ for two months exhibited the lowest expression levels of stemness markers CD133, CD15, Nestin, and transcription factors Sox2 and Klf4. Cells with simultaneous knockout showed the least expression, followed by single knockouts, compared to the control. **C** HIF1α and HIF2α knockout CD133^−^CD15^−^ cells cultured with TMZ for two months exhibited almost no aggregation formation. Aggregation formation was significantly reduced in single knockouts compared to the control. **D** Apoptosis and necrosis rates were most significantly increased in cells with simultaneous HIF1α and HIF2α knockouts, followed by single knockouts, compared to the control. **E** Cell cycle analysis revealed that control cells arrested in the G2/M phase, while HIF1α and HIF2α knockout cells entered the S phase. **F** The rates of β-Gal-positive and C_12_FDG-positive cells decreased most significantly in simultaneous HIF1α and HIF2α knockout cells, with intermediate decreases in single knockouts compared to the control. **G** RT-qPCR analysis showed the lowest expression of SASP factors, including IL1a, IL1b, IL6, IL8, CCL2, and others, in simultaneous knockouts, followed by single knockouts, compared to the control in U118 CD133^−^CD15^−^ cells. **H** GO analysis of miRNA sequences from simultaneous and single HIF1α and HIF2α knockouts revealed activation of terms associated with senescence and stemness, such as stem cell population maintenance, DNA replication, cell cycle arrest, centrosomes, and p53 binding. **I** KEGG pathway analysis of miRNA sequences from simultaneous and single HIF1α and HIF2α knockouts indicated activation of pathways associated with senescence and stemness, including cell cycle regulation, cellular senescence, Wnt signaling, regulation of pluripotency in stem cells, focal adhesion, and autophagy. ^*^P < 0.05, ^**^P < 0.01, and ^#^*P* > 0.05 were determined using Student’s *t*-test.

Proteomic, mRNA, and miRNA sequence analyses demonstrated the activation of the HIF1 signaling pathway under TMZ treatment (Fig. 1F, Fig. 2B, E). GO term enrichment indicated significant involvement of hypoxia-responsive processes (Fig. 2D, F), while GSEA analysis revealed that the Hallmark-hypoxia pathway was markedly upregulated (Fig. 6A). These findings prompted investigations into whether HIF1α and HIF2α regulate GBM cell senescence and the subsequent formation of aggregation stem-like cells.

CD133^−^CD15^−^ GBM cells exhibited high expression levels of HIF1α and HIF2α after two weeks of TMZ exposure, during which the cells displayed senescent characteristics (Fig. S15A). Western blot analysis further confirmed elevated HIF1α and HIF2α levels in CD133^−^CD15^−^ GBM cells treated with TMZ for 3, 6, and 9 days (Fig. S15B). Knockout of HIF1α and HIF2α in CD133^−^CD15^−^ GBM cells (Fig. S15C, Fig. S16A) significantly reduced aggregation formation (Fig. 6C, Fig. S16B) and stemness marker expression, including CD133, CD15, Nestin, Sox2, and Klf4, particularly under simultaneous knockout of HIF1α and HIF2α after TMZ treatment (Fig. 6B, Fig. S16A).

The role of HIF1α and HIF2α in senescence and apoptosis was further examined. Cells with dual knockout exhibited the highest apoptosis and necrosis rates (Fig. 6D, Fig. S1C) and the most substantial decrease in β-Gal or C_12_FDG positivity (Fig. 6F, Fig. S16D, E). Single knockouts of HIF1α or HIF2α led to intermediate β-Gal or C_12_FDG positivity and apoptosis rates compared to the control (Fig. 6D, F, Fig. S16C–E). Cell cycle analysis revealed that control cells arrested in the G2 phase, whereas dual knockout cells entered the S phase (Fig. 6E). RT-qPCR results showed significantly lower SASP marker expression (e.g., IL1a, IL1b, IL6, IL8, CCL2, and ATM) and the most pronounced upregulation of MSH2, a senescence-inhibiting gene, following dual knockout (Fig. 6G). Single knockouts yielded intermediate levels of these changes.

To eliminate the confounding effects of TMZ on senescence and stemness, cells were cultured in a hypoxic environment without TMZ, and miRNA sequence analysis was performed. GO term analysis identified activated processes related to senescence and stemness, including stem cell population maintenance, DNA replication, cell cycle arrest, centrosome activity, and p53 binding (Fig. 6H). KEGG pathway analysis further highlighted the activation of pathways associated with cellular senescence, Wnt signaling, pluripotency regulation, focal adhesion, and autophagy, confirming that both HIF1α and HIF2α regulate cell senescence and drive the formation of cancer stem-like cells (Fig. 6I).

## Discussion

TMZ is a cornerstone in GBM treatment, yet it extends overall survival (OS) by only a few months [20]. Despite its widespread use, GBM frequently recurs, with recurrent tumors being more malignant than primary GBM [2]. While TMZ effectively eliminates the majority of GBM cells, a small subset survives and acts as seeds for tumor recurrence. Identifying these residual cells and elucidating their transformation after TMZ treatment is crucial for devising more effective therapeutic strategies for GBM.

Recurrent GBM following TMZ treatment exhibits enhanced stemness compared to primary GBM, with three possible explanations [2, 21]. First, TMZ may preferentially eliminate differentiated cells, thereby increasing the relative proportion of stem-like cells in recurrent tumors. Second, TMZ may stimulate the proliferation of pre-existing glioma stem cells (GSCs). Third, it may induce the dedifferentiation of differentiated GBM cells into stem-like cells. Auffinger et al*.* [12] demonstrated that TMZ-induced cell death was 28% higher in pre-therapy GSCs compared to non-GSCs, a finding corroborated by other studies [22, 23]. These results suggest that the increased GSC pool in recurrent GBM predominantly arises from the conversion of non-GSCs into new GSCs. This aligns with evidence indicating that dedifferentiation can occur in GBM under specific conditions [13, 24]. Consequently, recurrent GBM likely harbors stem-like cells formed via dedifferentiation rather than pre-existing GSCs. Auffinger et al. [12] and Safa et al*.* [24] investigated this phenomenon, but the low serum content (DMEM + 1% FBS) used in their cell culture models raised the possibility that stem-like cells were induced due to low FBS levels rather than TMZ treatment.

To address this concern, in our study, CD133^−^CD15^−^ cells [18, 25] were cultured in DMEM + 10% FBS in the presence of BMP2 [26] during and after TMZ exposure, resulting in the formation of aggregations. Immunofluorescence staining and western blot analysis confirmed that these aggregations highly expressed stemness markers, including CD133, CD15, Nestin, and transcription factors Sox2 and Klf4. In vivo studies yielded similar results. These findings strongly suggest that TMZ induces differentiated GBM cells highly stemness expression.

We further examined the characteristics of newly formed aggregation stem-like cells. Consistent with previous studies [27, 28], these cells exhibited lower rates of apoptosis and necrosis than control cells exposed to the same TMZ dosage. Bioinformatic analyses revealed that these cells underwent cell cycle arrest, particularly at the G2/M checkpoint, leading to slower proliferation. However, upon TMZ treatment, control cells arrested in the G2 phase, while aggregation stem-like cells progressed to S phases, facilitating increased growth. This observation may explain the enhanced chemotherapy resistance observed in recurrent GBM. Additionally, sequence analysis revealed significant alterations in invasion pathways, including ECM-receptor interaction, focal adhesion, and integrin binding. Transwell assays confirmed that aggregation stem-like cells possessed greater invasive capacity. Co-culture experiments further demonstrated that control cells cultured with conditioned medium from aggregation stem-like cells exhibited increased invasion. These findings were corroborated by in vivo studies, which showed that mice implanted with aggregation stem-like cells exhibited shorter survival without TMZ treatment, likely due to the lower invasion of control cells. Under TMZ treatment, the proportion of aggregation stem-like cells increased, correlating with shorter survival. Collectively, these results indicate that aggregation of stem-like cells contributes to the malignancy of recurrent GBM by enhancing invasiveness and therapy resistance.

The formation of aggregation stem-like cells was then investigated. Previous studies have demonstrated that chemotherapy induces senescence in a small fraction of cancer cells while killing the majority [7, 29]. The results of this study, including Gene Ontology (GO) analysis, KEGG pathway analysis, and GSEA hallmark analysis, confirmed that TMZ treatment induced senescence in differentiated GBM cells. Senescence-associated secretory phenotype (SASP) detection revealed significant alterations in interleukins (IL1a, IL1b, IL6, IL8), chemokines (CCL2, CXCL3), and other factors [30]. These findings support the hypothesis that senescence enables some tumor cells to evade the cytotoxic effects of anticancer drugs, eventually facilitating regrowth with increased stemness and invasiveness potential [31, 32].

Our experiments revealed that aggregation stem-like cells were derived exclusively from C12FDG-positive senescent cells and not from C12FDG-negative cells. Regarding the mechanism by which senescent cells give rise to stem-like cells, previous studies have suggested that polyploidization resulting from cell cycle arrest and DNA replication plays a critical role [33]. These senescent polyploid cells reportedly divide into smaller cells via budding, producing colonies with stem-like characteristics under the regulation of SASP [11].

Our findings confirm that aggregation of stem-like cells originates from differentiated GBM senescent cells under TMZ treatment. However, the precise mechanisms by which these cells escape senescence and acquire stem-like properties under SASP regulation warrant further investigation. Notably, the frequency of senescence escape was approximately 1 in 10⁶ differentiated GBM cells, indicating that this phenomenon is relatively rare. Previous studies have shown that senescent cells arise from differentiated cells rather than cancer stem cells (CSCs), and chemotherapy-induced SASP promotes the dedifferentiation of differentiated cells into cancer stem-like cells rather than affecting CSCs directly [34]. In this study, we demonstrated that senescent cells were induced from differentiated GBM cells and subsequently promoted the formation of aggregation stem-like cells. These findings provide further evidence that higher stemness is expressed in GBM under TMZ treatment, with cellular senescence playing a pivotal role in this process.

To investigate the molecular mechanisms underlying our findings, we focused on HIF1α and HIF2α, given their involvement in the activation of the HIF1 pathway following TMZ treatment. Previous studies have shown that therapeutic stress induces a hypoxia-like response independent of intratumoral hypoxia [35]. Auffinger et al*.* [12, 36] reported that recurrent GBM post-TMZ treatment exhibits increased expression of HIF1α and HIF2α. These factors subsequently upregulate EGF, Sox2, and Klf4, which are associated with stemness, therapy resistance, and invasiveness [5, 13]. Similarly, radiation therapy has been shown to stabilize HIF1α protein expression, promoting malignant progression in GBM [37]. Silencing HIF1α combined with radiation therapy enhances therapeutic efficacy through regulation of cell cycle and apoptosis pathways [38].

Furthermore, HIF1α and HIF2α have been implicated in the regulation of SASP components [39, 40], such as IL1a, IL1b, IL6, IL8, CCL2, CDKN1A, CDKN2B, and FGF2. These findings provide additional evidence that HIF1α and HIF2α promote GBM cell senescence, subsequently inducing the formation of GBM stem-like cells. This aligns with the established mechanism wherein SASP facilitates the expression of stemness and invasiveness in tumor cells [41, 42]. Our study confirmed elevated expression levels of HIF1α and HIF2α in aggregation stem-like cells and senescent cells under TMZ treatment. Knockout experiments demonstrated that cells lacking HIF1α and HIF2α exhibited decreased β-galactosidase (β-Gal) and C12FDG positivity, reduced SASP expression, and lower levels of stemness markers. Bioinformatic analyses supported these observations, revealing that senescence- and stemness-associated pathways were activated in cells expressing HIF1α and HIF2α.

Targeting both HIF1α and HIF2α simultaneously may represent an effective strategy for improving GBM prognosis. This dual-targeting approach potentially addresses the limitations of single-target therapies, which have not achieved success in clinical trials despite the development of several HIF inhibitors, including CCL-779, SCH66336, and FK228 [43]. To date, no HIF-targeted therapies have been approved for GBM treatment.

## Conclusion

**Fig. 7:**
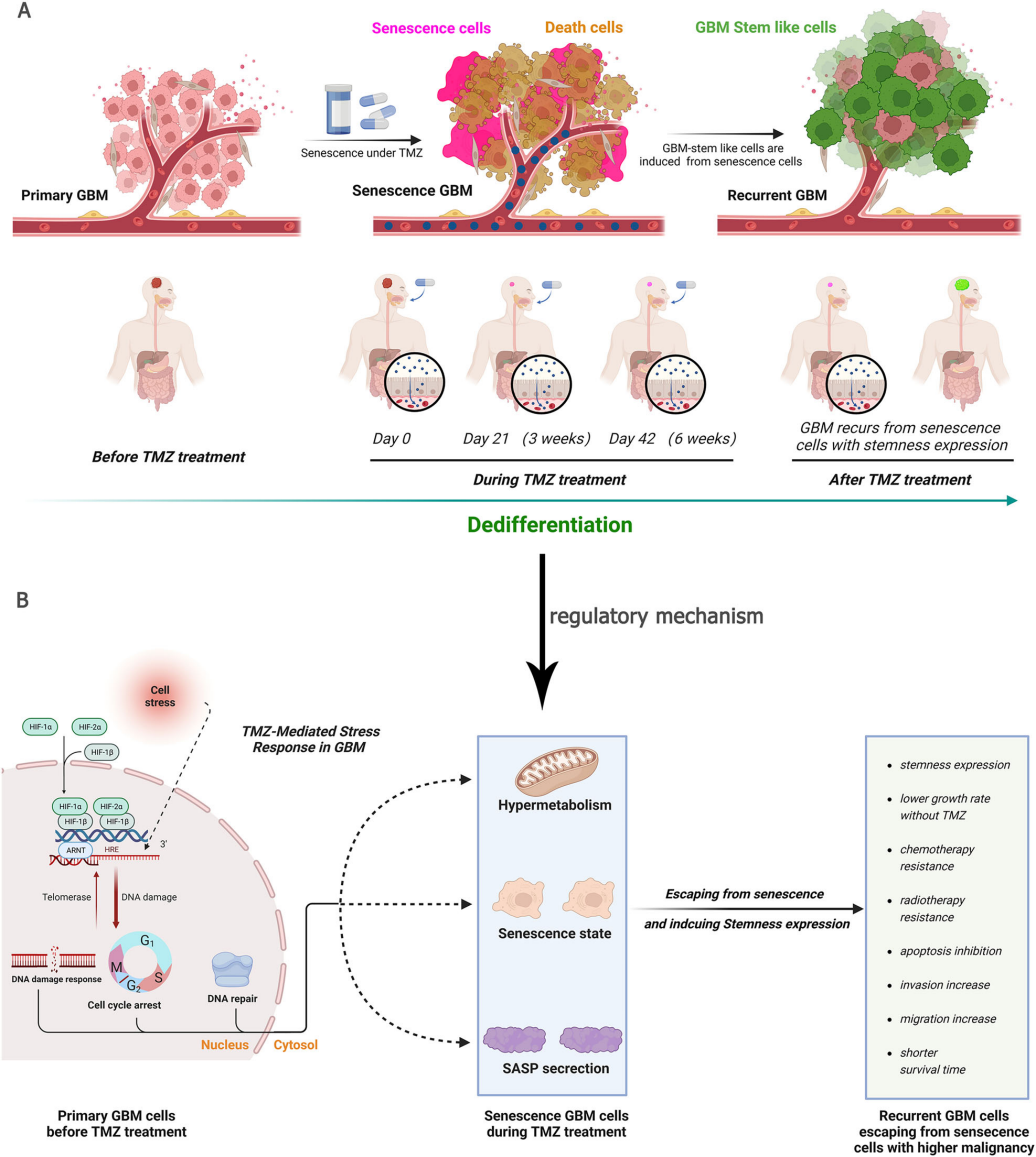
Dedifferentiation process in differentiated GBM cells under TMZ treatment and its mechanism. **A** Differentiated GBM cells exposed to TMZ undergo cellular senescence in addition to cell death. These senescent GBM cells experience growth retardation and hypermetabolism, ultimately dividing into new cells characterized by enhanced stemness, higher invasion potential, reduced proliferation, and increased therapy resistance. This process contributes to the heightened malignancy of recurrent GBM. **B** HIF1α and HIF2α play critical roles in the formation of senescent cells, promoting hypermetabolism, cell cycle arrest, and DNA damage response. These cells subsequently divide rapidly into more malignant progeny, thereby accelerating GBM recurrence.

In summary, TMZ treatment provides only a transient delay in GBM recurrence, which is often followed by rapid progression characterized by increased malignancy. Our findings suggest that differentiated GBM cells exposed to TMZ enter a state of cellular senescence, marked by growth retardation and hypermetabolism. These senescent cells eventually divide into new cells with enhanced stemness, invasiveness, reduced proliferation, and therapy resistance, thereby contributing to the increased malignancy of recurrent GBM (Fig. 7A). Both HIF1α and HIF2α are critical in inducing senescence, resulting in hypermetabolism, cell cycle arrest, and DNA damage responses, and subsequently driving the formation of more malignant cells (Fig. 7B). Inhibiting the formation of senescent cells represents a key clinical objective. Eliminating these cells could reduce chemotherapy resistance and invasiveness, thereby prolonging recurrence-free survival and improving patient outcomes.

This study utilized multi-omics analyses, including proteomic, mRNA, and miRNA sequencing, to elucidate the molecular mechanisms underlying these processes. However, limitations exist in our study. For instance, we focused on the long-term effects of TMZ exposure, neglecting the short-term impacts of TMZ on GBM cells. Additionally, while we examined the effects of TMZ, we did not address the influence of the tumor microenvironment, such as hypoxia. The detailed mechanisms by which stem-like cells escape from senescent cells also remain insufficiently understood. Future studies will aim to address these limitations and investigate whether radiation therapy induces similar phenomena as TMZ treatment.

## Supplementary information

Supplementary Tables

Table S2

Table S3

Table S4

Table S5

Table S6

Table S7

Table S8

Table S9

Table S10

Supplementary Figures and Figure Legends

Original Data

## Data Availability

All data are available in the manuscript or supplementary materials and can also be obtained from the corresponding author upon reasonable request.
