# Supplementary material for: Temozolomide promotes glioblastoma stemness expression through senescence-associated reprogramming via HIF1α/HIF2α regulation
Source: Cell Death Dis. 2025 Apr 19;16(1):317. doi: 10.1038/s41419-025-07617-w (PMC12009364; doi:10.1038/s41419-025-07617-w)
Supplement: Supplementary file 1 — Supplementary Tables [file 41419_2025_7617_MOESM1_ESM.pdf]

**Table S1 Differentiation protein expression involved in invasion and stemness**

| Gene name | U87 | | | U118 | | |
|---|---|---|---|---|---|---|
| | Agg/Con ratio | Agg/Con P value | Expression | Agg/Con ratio | Agg/Con P value | Expression |
| PROCR | 1.64339029 | 0.002590493 | Up | 1.542069014 | 0.001487405 | Up |
| SLC25A22 | 1.684460012 | 0.001237498 | Up | 1.679189709 | 0.010561844 | Up |
| ALDH1A3 | 1.666207324 | 0.022763125 | Up | 12.33089355 | 0.000199883 | Up |
| HMGA2 | 2.734122498 | 0.004089914 | Up | 6.415397755 | 0.004453681 | Up |
| ABCA1 | 1.822717988 | 0.002792701 | Up | 5.821167423 | 0.001473723 | Up |
| ABCC4 | 1.642151438 | 0.021642199 | Up | 1.850064674 | 0.001539279 | Up |
| YAP1 | 1.618440004 | 0.019292912 | Up | 1.683824399 | 0.020803378 | Up |
| CHD1L | 2.2706641 | 0.023733524 | Up | 3.531937176 | 0.001306488 | Up |
| ALDH1A3 | 1.666207324 | 0.022763125 | Up | 12.33089355 | 0.000199883 | Up |
| BCL2L12 | 1.875979596 | 0.011402308 | Up | 1.787983912 | 0.004826449 | Up |
| CPA4 | 1.724904096 | 0.009107091 | Up | 1.724904096 | 0.009107091 | Up |
| FMNL3 | 1.938707113 | 0.003569857 | Up | 1.521213265 | 0.011327822 | Up |
| MAD1L1 | 3.909310763 | 0.013593438 | Up | 2.128446532 | 0.049159972 | Up |
| AKAP12 | 1.612046539 | 0.000607309 | Up | 1.663916656 | 0.020692759 | Up |
| EFNB2 | 2.292769715 | 0.040322356 | Up | 3.238374673 | 0.004184769 | Up |
| LGR4 | 3.331164947 | 0.001778385 | Up | 3.38951925 | 0.02671365 | Up |
| ALDH2 | 0.459492923 | 0.042712638 | Down | 0.514765935 | 0.011881771 | Down |
| CDH13 | 1.724591559 | 0.028315429 | Up | 1.700706001 | 0.015546471 | Up |
| TFPI2 | 4.510653521 | 0.005343053 | Up | 11.91634982 | 0.001598346 | Up |
| ACP2 | 2.460589362 | 0.000467601 | Up | 2.460589362 | 0.000467601 | Up |
| ADCY6 | 1.930759618 | 0.002655308 | Up | 1.611433749 | 0.003022489 | Up |
| AGPAT5 | 2.113259514 | 0.009295051 | Up | 1.772299555 | 0.042227232 | Up |
| ARHGAP15 | 3.530810397 | 0.021776376 | Up | 1.834796709 | 0.019972447 | Up |
| DCUN1D3 | 2.098061453 | 0.000721579 | Up | 2.004772704 | 0.006440267 | Up |
| ERP44 | 1.622378885 | 0.018466168 | Up | 1.60954367 | 0.043383631 | Up |
| MRC2 | 1.501926877 | 0.019714605 | Up | 0.337649214 | 0.00529449 | Down |
| PCDHGA9 | 1.986472628 | 0.001890832 | Up | 9.528483007 | 0.009568114 | Up |
| SLC38A2 | 1.643606596 | 0.020262963 | Up | 2.625518759 | 0.000264924 | Up |
| ANXA5 | 0.293085125 | 0.000472448 | Down | 0.425817687 | 0.004006131 | Down |
| GBP1 | 0.286989675 | 0.001355958 | Down | 0.291345488 | 0.009519559 | Down |
| LAMC1 | 0.517711463 | 0.048023484 | Down | 0.510026322 | 0.007799092 | Down |
| PYGB | 0.221578436 | 0.000112287 | Down | 0.322638967 | 0.00044365 | Down |
| NCK1 | 0.617162452 | 0.00380581 | Down | 0.325194115 | 0.004493593 | Down |
| PRDX2 | 0.206533566 | 1.08517E-05 | Down | 0.075807349 | 1.75031E-05 | Down |
| MCM4 | 0.397207912 | 0.00282304 | Down | 0.578415801 | 0.007850114 | Down |
| SOAT1 | 0.355221771 | 0.0001752 | Down | 0.648680302 | 0.033116151 | Down |
| UBXN1 | 0.6155213 | 0.027240479 | Down | 0.494369153 | 0.002284093 | Down |
| FMR1 | 0.352006215 | 0.000633397 | Down | 0.426777216 | 0.001415617 | Down |
| CTBP1 | 0.639960219 | 0.021014772 | Down | 0.180271591 | 0.001145724 | Down |
| NCAPH | 0.558451077 | 0.003983147 | Down | 0.646013446 | 0.029448201 | Down |

| | | | | | | |
|---|---|---|---|---|---|---|
| MGME1 | 0.269693781 | 0.008006855 | Down | 0.269693781 | 0.008006855 | Down |
| C20orf27 | 0.32973774 | 7.36427E-05 | Down | 0.461172556 | 0.022721122 | Down |
| GOLT1B | 1.690862825 | 0.002939111 | Up | 1.98004382 | 0.003098555 | Up |
| ITGA2 | 1.884077767 | 0.001543564 | Up | 1.653649489 | 0.022124598 | Up |
| CD40 | 1.642376519 | 0.001185476 | Up | 2.525318085 | 0.009518257 | Up |
| TRIM22 | 2.350875526 | 0.023421135 | Up | 3.394343249 | 0.013234349 | Up |
| PAPPA | 2.66044296 | 0.00248275 | Up | 10.27873008 | 0.004473294 | Up |
| ITGB3 | 1.604448471 | 0.00613092 | Up | 39.6232232 | 4.0914E-06 | Up |
| PTPRE | 1.558311858 | 0.00433624 | Up | 5.008937909 | 0.003726418 | Up |
| ADCK5 | 2.211913924 | 0.010047887 | Up | 2.510016256 | 0.038427767 | Up |
| ANO6 | 1.662055729 | 0.005545972 | Up | 1.662055729 | 0.005545972 | Up |
| GALNT7 | 1.716855183 | 0.012206614 | Up | 1.716855183 | 0.012206614 | Up |
| GTPBP2 | 2.678059277 | 0.002723563 | Up | 1.601133177 | 0.008240658 | Up |
| IFITM2 | 3.26574891 | 0.001002159 | Up | 1.801622723 | 0.015511851 | Up |
| MTCH2 | 1.653421953 | 0.002304593 | Up | 2.138163911 | 0.003573504 | Up |
| MTFR1 | 1.82304682 | 0.045720925 | Up | 1.82304682 | 0.045720925 | Up |
| MYL9 | 2.869622154 | 0.000657681 | Up | 2.976227587 | 0.000429837 | Up |
| SHC1 | 1.606537062 | 0.00246315 | Up | 2.119041389 | 0.014227499 | Up |
| SIRT6 | 2.842612983 | 0.005148005 | Up | 1.529280061 | 0.00245528 | Up |
| TRIM21 | 0.468608681 | 0.003418182 | Down | 0.587910127 | 0.010807495 | Down |
| PTPRG | 0.552503419 | 0.023411176 | Down | 0.509613649 | 0.00376942 | Down |
| GSTM2 | 0.274882336 | 0.001269599 | Down | 0.558932731 | 0.027224447 | Down |
| VEPH1 | 0.579214294 | 0.028336165 | Down | 0.542508803 | 0.009766722 | Down |
| CUL5 | 0.558596177 | 0.002057316 | Down | 0.634696292 | 0.018025087 | Down |
| EHD2 | 0.442362897 | 0.002535756 | Down | 0.480175315 | 0.003943868 | Down |

Table S11 GSEA analysis of the hallmark according to differentiation expression genes

| | Hallmark | ES | NES | pvalue | p.adjust |
|---|---|---|---|---|---|
| U87 | AGING | 0.653644837 | 1.95956208 | 7.22E-06 | 4.60E-05 |
| | FRIDMAN_SENESCENCE_UP | 0.654627235 | 1.857033052 | 0.000531685 | 0.002764764 |
| | SENESCENCE_INFLAMMATORY_GENES | 0.627150418 | 1.844277137 | 0.000351987 | 0.002287919 |
| | EPITHELIAL_MESENCHYMAL_TRANSITION | 0.725035096 | 2.356584605 | 1.00E-10 | 2.55E-09 |
| | G2M_CHECKPOINT | -0.607274428 | -2.274846425 | 1.00E-10 | 2.55E-09 |
| | ATR_SUPPRESSED_TARGETS | -0.696105192 | -1.954468031 | 0.000283709 | 0.002287919 |
| | E2F_TARGETS | 0.653644837 | 1.95956208 | 7.22E-06 | 4.60E-05 |
| | SASP_COPPE | 0.795242572 | 2.147799142 | 2.32E-07 | 6.04E-06 |
| | MITOTIC_SPINDLE | -0.516380681 | -1.940309268 | 4.28E-07 | 3.12E-06 |
| | MYC_TARGETS_V1 | -0.395562368 | -1.476387655 | 0.002777726 | 0.010317266 |
| | APOPTOSIS | 0.602377972 | 1.901542905 | 8.46E-06 | 4.79E-05 |
| | TNFA_SIGNALING_VIA_NFKB | 0.601006037 | 1.956063655 | 2.25E-07 | 2.30E-06 |
| | P53_PATHWAY | 0.545122092 | 1.77420474 | 5.10E-05 | 0.000236324 |
| | COMPLEMENT | 0.501566773 | 1.617132782 | 0.001450824 | 0.005691694 |
| | COAGULATION | 0.644665104 | 1.944786264 | 1.27E-05 | 6.49E-05 |
| | DUY_CISG_UP | 0.604562267 | 1.961069616 | 5.03E-07 | 6.53E-06 |
| | HYPOXIA | 0.635268159 | 2.048469026 | 1.76E-08 | 2.24E-07 |
| | UV_RESPONSE_DN | 0.50254338 | 1.566128925 | 0.002989288 | 0.010889549 |
| | INFLAMMATORY_RESPONSE | 0.612688606 | 1.962149625 | 2.92E-07 | 2.48E-06 |
| U118 | G2M_CHECKPOINT | -0.448870134 | -1.429109502 | 0.006665003 | 0.028326264 |
| | TNFA_SIGNALING_VIA_NFKB | 0.574879845 | 1.910784555 | 2.63E-07 | 1.34E-05 |
| | P53_PATHWAY | 0.535963403 | 1.776692157 | 5.70E-06 | 9.69E-05 |
| | UV_RESPONSE_DN | -0.524412753 | -1.608459916 | 0.001789167 | 0.013035359 |
| | DUY_CISG_UP | 0.635605387 | 2.103660013 | 1.00E-10 | 2.60E-09 |
| | HALLMARK INFLAMMATORY_RESPONSE | 0.559753293 | 1.846275672 | 1.44E-06 | 3.68E-05 |
| | COMPLEMENT | 0.45754483 | 1.51354693 | 0.002108431 | 0.013441249 |
| | XENOBIOTIC_METABOLISM | 0.482555119 | 1.586900596 | 0.000534472 | 0.004543011 |
| | INFLAMMATORY_RESPONSE | 0.586791351 | 1.814374429 | 0.000283057 | 0.003679735 |
| | HEMATOPOIETIC_STEM_CELL_UP | -0.41641134 | -1.36606057 | 0.009225004 | 0.039975019 |
| | SENESCENCE_INFLAMMATORY_GENES | 0.56916797 | 1.720450393 | 0.000984155 | 0.008529342 |
| | IL6_JAK_STAT3_SIGNALING | 0.533406577 | 1.564943426 | 0.005871441 | 0.027222136 |
| | INTERFERON_GAMMA_RESPONSE | 0.443228294 | 1.472394967 | 0.004295787 | 0.024342794 |
| | KRAS_SIGNALING_UP | 0.481487591 | 1.583812104 | 0.000329111 | 0.003356927 |
| | REGULATION_OF_MITOTIC_CELL_CYCLE | -0.547423946 | -1.679394785 | 0.000778731 | 0.020247017 |
| | MITOTIC_SPINDLE | -0.493053817 | -1.641861505 | 2.03E-05 | 0.000680125 |
| | PROTEIN_SECRETION | -0.495291908 | -1.53513565 | 0.009711427 | 0.041273567 |
| | HEDGEHOG_SIGNALING | -0.63712589 | -1.701050161 | 0.002179613 | 0.013895035 |

| | | | | | |
|---|---|---|---|---|---|
| GSE 100736 | UV_RESPONSE_DN | -0.465941857 | -1.510069557 | 0.00378118 | 0.019284017 |
| | APICAL_JUNCTION | -0.469537413 | -1.557875894 | 0.000470281 | 0.004796863 |
| | P53_PATHWAY | 0.362576594 | 1.515562676 | 0.000146685 | 0.00249364 |
| | HYPOXIA | 0.348673959 | 1.468438956 | 0.00105447 | 0.008962997 |
| | COMPLEMENT | 0.312097867 | 1.316262671 | 0.009393509 | 0.041273567 |

**Table S12 Common elements of differential expression genes with SASP expression according to proteome, mRNA and miRNA sequence analysis**

**U87 mRNA sequence**

| 1) | Common elements in SASP and mRNA DEGs |
|---|---|
| DEGs | IL6, CXCL3, CCL26, FGF2, MMP7, TIMP3, NOS1, COLQ, COL11A2, COL5A1, LAMB3, CDK10, CDK8, ATM, CDC25C, ITGBL1, ITGA4 |
| Total | 17 |
| 2) | Common elements in SASP and mRNA transcript DEGs |
| DEGs | IL7, OCLN, MMP2, MMP19, TIMP2, EGFR, COL1A2, LAMA3, LAMB1, LAMB2, CDK19, CDK13, MSH6, ITGA7 |
| Total | 14 |
| 3) | Common elements in SASP, mRNA DEGs and mRNA transcript DEGs |
| DEGs | CXCL2, CXCL8, CCL2, CCL3, CCL5, CSF3, HGF, NGF, IGFBP3, IGFBP4, IGFBP5, IGFBP6, IGFBP7, MMP14, MMP15, TIMP1, CTSB, ICAM1, NOS3, FN1, IGFN1, COL5A2, COL17A1, COL6A3, COL5A3, COL3A1, COL15A1, COL4A6, COL6A1, LAMC2, FAT2, LAMA4, CDKN2D, CDKN3, CDK5R2, CDKL2, CDK16, CCNB1, CCNB2, CDKN1A, CDC25A, CDC25B, PDGFRB, ITGB2-AS1, ITGB3 |
| Total | 45 |

**U118 mRNA sequence**

| 1) | Common elements in SASP and mRNA DEGs |
|---|---|
| DEGs | OCLN, MMP10, MMP15, COL24A1, CDKN1B, CDKN1C, CCNB1, CCNB2, CCNB1IP1, MSH2, ITGB2-AS1, ITGB8 |
| Total | 12 |
| 2) | Common elements in SASP and mRNA transcript DEGs |
| DEGs | CXCL3, HGF, IGFBP7, MMP2, MMP14, CTSB, TNFRSF1A, COL6A3, COL5A1, CDK16, ITGB1 |
| Total | 11 |
| 3) | Common elements in SASP, mRNA DEGs and mRNA transcript DEGs |
| DEGs | IL6, CXCL2, CXCL5, CXCL8, CXCL10, CXCL11, CXCL12, CCL2, CCL3, CCL4, CCL5, CCL26, CSF2, CSF3, EREG, NGF, IGFBP3, IGFBP4, IGFBP5, IGFBP6, MMP1, MMP3, MMP7, MMP9, MMP12, MMP19, TIMP3, TIMP4, ICAM1, TNFRSF11B, FAS, EGFR, NOS3, FN1, COL5A2, COLQ, COL5A3, COL15A1, COL4A6, COL6A1, COL11A1, COL9A2, COL1A2, COL4A2, COL27A1, COL16A1, FAT2, LAMA4, LAMB3, LAMC1, NTN4, FAT4, DCHS2, LAMA3, LAMB1, LAMA2, NTNG1, LAMB2, LAMA5, MEGF9, CDKN2C, CDK10, CDK5R2, CDK18, CDK5R1, CDK11A, CDK17, CCNT2, CDK19, CDKL1, CDKAL1, CDK11B, CDK13, CDKN1A, ATM, ATR, CDC25B, CDC25C, MSH6, MSH5, EXO1, ITGA6, ITGB3, ITGBL1, ITGA4, ITGAD, ITGAX, ITGA11, ITGA7, ITGA10, ITGAM, ITGA8, ITGA9-AS1 |
| Total | 93 |

**GSE100736 mRNA sequence**

| 1) | Common elements in SASP and mRNA DEGs |
|---|---|
| DEGs | IL7, CXCL14, CCL2, CCL26, OCLN, MIF, EREG, EGF, IGFBP2, IGFBP3, IGFBP4, IGFBP5, IGFBP6, MMP2, MMP14, MMP15, TIMP3, CTSB, TNFRSF11B, COL5A2, COL17A1, COL6A3, COL3A1, COL15A1, COL4A6, COL6A1, COL11A1, LAMA4, LAMA3, NTNG1, CDKN2D, CDKN2C, CDKN1C, CDKN2A, CDKN2B, PDGFRB, ITGBL1, ITGA4, ITGB8, ITGA7, ITGA9-AS1 |
| Total | 41 |

**Proteome detection**

| 1) | Common elements in SASP and U87 differentially expressed proteins |
|---|---|
| DEPs | IGFBP5, MMP15, COL16A1 |
| Total | 3 |
| 2) | Common elements in SASP and U118 differentially expressed proteins |
| DEGs | FGF2, HGF, IGFBP3, IGFBP4, MMP2, MMP3, TIMP1, TIMP3, CTSB, EGFR, COL5A2, COL6A3, COL5A1, COL6A1, COL1A2, LAMA4, NTN4, NTNG1, LAMB2, ATM, PDGFRB, ITGB8, ITGAX, ITGA11, ITGA7 |
| Total | 25 |
| 3) | Common elements in SASP, U87 and U118 differentially expressed proteins |
| DEGs | IGFBP7, FAS, FN1, LAMC1, MSH2, MSH6, ITGA6, ITGB3, ITGA4, ITGA8 |
| Total | 10 |

**U87 miRNA sequence**

| 1) | Common elements in SASP and U87 DE-miRNA targets genes |
|---|---|
| DE_miRNA target_genes | IL15, CXCL14, CCL5, CSF3, AREG, IGFBP2, IGFBP3, IGFBP4, IGFBP7, MMP2, MMP15, TIMP2, TIMP3, ICAM1, ICAM3, TNFRSF1A, TNFRSF11B, EGFR, FN1, IGFN1, COL5A2, COL6A3, COL5A3, COL3A1, COL15A1, COL4A6, COL5A1, COL11A1, COL1A2, COL4A2, COL27A1, LAMA4, LAMC1, LAMA3, LAMA5, MEGF9, CDKN2D, CDKN2C, CDKN1B, CDK5R2, CDK16, CDK8, CDK18, CDK5R1, CDK11A, CDK17, CDK11B, CDK13, CDKN2A, MYC, ATM, CDC25A, CDC25B, MSH5, ITGA6, ITGA11, ITGA8 |
| Total | 57 |

**U118 miRNA sequence**

| 1) | Common elements in SASP and U118 DE-miRNA targets genes |
|---|---|
| DE_miRNA target_genes | COL5A2, COL3A1, COL6A3, IGFBP2, ITGA4, CDC25B, FN1, IGFBP5, CDK5R2, COL16A1, COL9A2, CDK11B, CDK11A, COL11A1, NTNG1, ITGA10, CDK18, LAMC1, IGFN1, LAMC2, LAMB3, ITGA8, CXCL12, COL17A1, FAS, CDKN1C, ATM, CDKN1B, TNFRSF1A, MMP13, ITGA7, CDK17, NTN4, NOS1, MMP14, COL4A2, CCNB1IP1, CDKL1, ITGA11, RAD51, ITGAX, ITGAM, MMP15, MMP2, CDK10, CCL5, IGFBP4, CDK5R1, CSF3, CCL16, TIMP2, LAMA3, MSH6, MSH2, ITGA6, ICAM3, ICAM1, COL5A3, CDKN2D, TIMP3, COLQ, LAMB2, CSF2RB, CDC25A, EREG, CXCL2, CXCL5, IL13, FAT2, PDGFRB, CDKAL1, CXCL14, CDK13, IGFBP3, |

| | |
|---|---|
| | ITGB8, EGFR, IL6, LAMA5, COL6A1, MIF, COL15A1, COL5A1, COL27A1, MEGF9, DCHS2, FGF2, EGF, OCLN, IL15, FAT4, TNFRSF11B, CDKN2A, CDKN2B, MYC, ATR, COL4A6, CDK16, TIMP1, COL11A2, MSH5, CDK19, CDKN1A, LAMA2, LAMA4, CTSB, COL1A2, LAMB1, NOS3 |
| Total | 108 |

**Table S13 Primary antibodies used for immunofluorescence staining**

| Antigens | Manufacturer | Catalogue numbers | Application |
|---|---|---|---|
| CD133 | Proteintech | 18470-1-AP | 1:300 |
| Nestin | Proteintech | 19483-1-AP | 1:300 |
| Sox2 | abcam | ab171380 | 1:200 |
| Klf4 | Proteintech | 11880-1-AP | 1:100 |
| HIF1A | abcam | ab179483 | 1:500 |
| HIF2A | NOVUS | NB100-132SS | 1:200 |

**Table S14 Primary antibodies used for western blotting**

| Antigens | Manufacturer | Catalogue numbers | Application |
|---|---|---|---|
| CD133 | Proteintech | 18470-1-AP | 1:2000 |
| CD15 | NOVUS | NB100-1831 | 2ug/ml |
| Nestin | Proteintech | 19483-1-AP | 1:1000 |
| Sox2 | abcam | ab171380 | 1:1000 |
| Klf4 | Proteintech | 11880-1-AP | 1:2000 |
| HIF1A | abcam | ab179483 | 1:1000 |
| HIF2A | abcam | ab207607 | 1:1000 |
| β-actin | Sangon Biotech | D191047-0100 | 1:5000 |

**Table S15 Sequences of primers used for RT–qPCR analysis**

| | | |
|---|---|---|
| IL1a | Forward(5'-3') | TGTATGTGACTGCCCAAGATGAA |
| | Reverse(5'-3') | GGATGGGCAACTGATGTGAAATA |
| IL1b | Forward(5'-3') | CTACGAATCTCCGACCACCACTA |
| | Reverse(5'-3') | CTCGTTATCCCATGTGTCGAAGA |
| IL6 | Forward(5'-3') | TGAAAGCAGCAAAGAGGCACT |
| | Reverse(5'-3') | TTCACCAGGCAAGTCTCCTCA |
| IL8 | Forward(5'-3') | TTCTAGGACAAGAGCCAGGAAGA |
| | Reverse(5'-3') | GGTCCACTCTCAATCACTCTCAG |
| MSH2 | Forward(5'-3') | ACAGTGCGCCTTTTCGAC |
| | Reverse(5'-3') | TGGATGCCTTATTTCCAGCTC |
| CCL2 | Forward(5'-3') | TCGCGAGCTATAGAAGAATCACC |
| | Reverse(5'-3') | GAATCCTGAACCCACTTCTGCTT |
| CDKN1A | Forward(5'-3') | CTGTCACTGTCTTGTACCCTTGT |
| | Reverse(5'-3') | CCCAGCAGAGGAACCACTACTA |
| CDKN2B | Forward(5'-3') | GCGTTCACTCCAATGTCTGC |
| | Reverse(5'-3') | CTTACTGAAGCCCACCTCGG |
| P53 | Forward(5'-3') | TCTGACTGTACCACCATCCACTA |
| | Reverse(5'-3') | TGTTCCGTCCCAGTAGATTACCA |
| CXCL3 | Forward(5'-3') | CGAAGTCATAGCCACACTCAAGA |
| | Reverse(5'-3') | CTGCAGGAAGTGTCAATGATACG |
| ATM | Forward(5'-3') | TCCCTCCACCTGCATATGTATCT |
| | Reverse(5'-3') | AACATCTTGGTCACGACGATACA |
| COL5A1 | Forward(5'-3') | CACCGGGCAGCTTATGATTACT |
| | Reverse(5'-3') | GCCGTCTCCTTCCGTGTAATATT |
| EGF | Forward(5'-3') | TGTGATTGCTTTCCTGGGTATGA |
| | Reverse(5'-3') | CAGGGCTGTATGGGCAAAGTATA |
| FGF2 | Forward(5'-3') | TCTCTGGCAGTTCCTTATGATAGA |
| | Reverse(5'-3') | ACAACTCCCATCACCAGCAG |
| IGFBP3 | Forward(5'-3') | CTTCTGCTGGTGTGTGGATAAGT |
| | Reverse(5'-3') | GGTCATGTCCTTGGCAGTCTTT |
| MMP1 | Forward(5'-3') | TGATAGCACATGACTTTCCTGGA |
| | Reverse(5'-3') | TCCTGCAGTTGAACCAGCTATTA |
| uPA | Forward(5'-3') | AACGACATTGCCTTGCTGAAGAT |
| | Reverse(5'-3') | GTGACTTCAGAGCCGTAGTAGTG |
| β-Actin | Forward(5'-3') | ACCCGCCGCCAGCTCACC |
| | Reverse(5'-3') | GGGGGGCACGAAGGCTCATC |

**Table S16 The sequences of sgRNA for knockout of HIF1α and HIF2α**

| Target | Oligonucleotide sequence(5'-3') |
|---|---|
| HIF1A | GAACTCACATTATGTGGAAG |
| HIF2A | CTTGGAGGGTTTCATTGCCG |
