# Supplementary material for: Temozolomide promotes glioblastoma stemness expression through senescence-associated reprogramming via HIF1α/HIF2α regulation
Source: Cell Death Dis. 2025 Apr 19;16(1):317. doi: 10.1038/s41419-025-07617-w (PMC12009364; doi:10.1038/s41419-025-07617-w)
Supplement: Supplementary file 11 — Supplementary Figures and Figure Legends [file 41419_2025_7617_MOESM11_ESM.pdf]

Sup 1

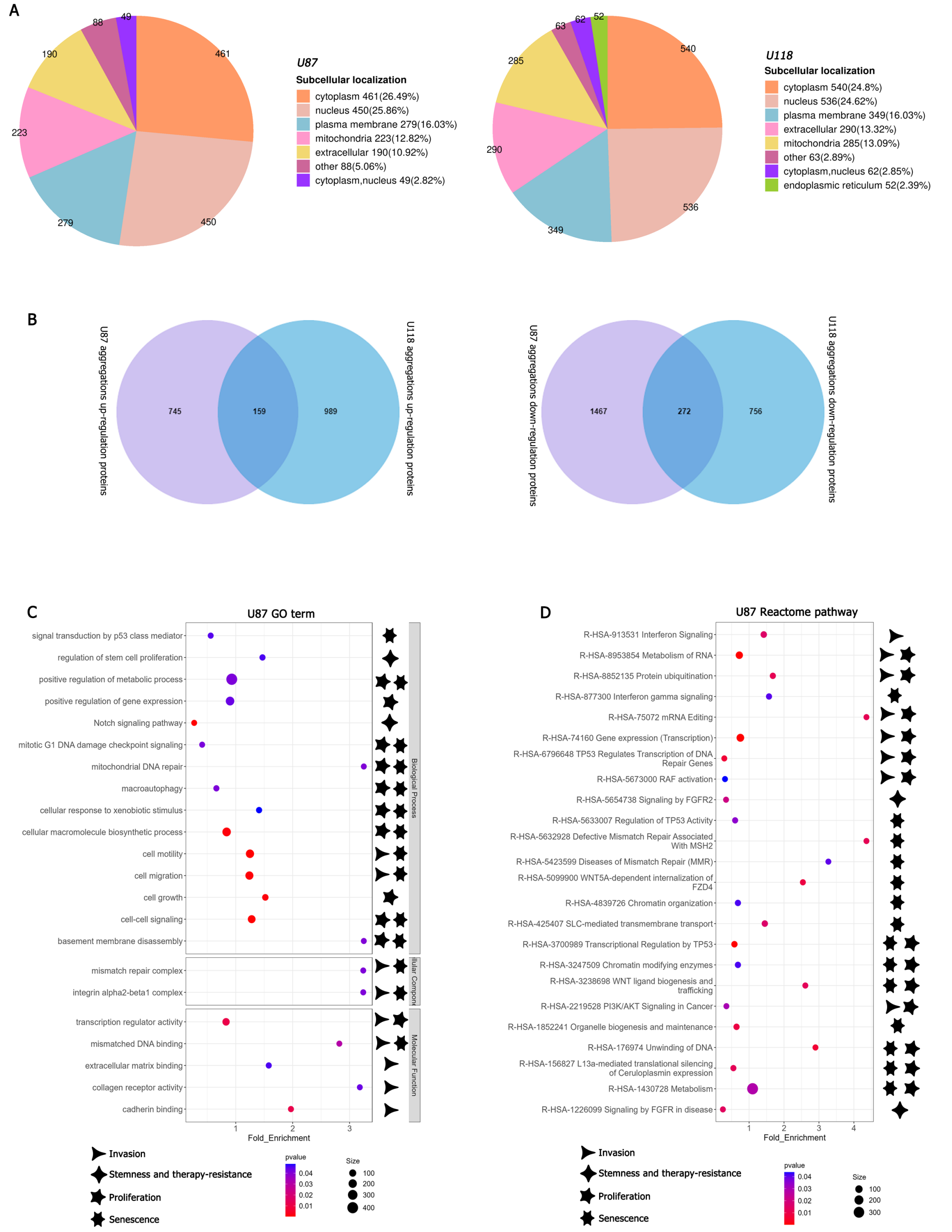
A
U87
Subcellular localization
cytoplasm 461(26.49%)
nucleus 450(25.86%)
plasma membrane 279(16.03%)
mitochondria 223(12.82%)
extracellular 190(10.92%)
other 88(5.06%)
cytoplasm,nucleus 49(2.82%)
U118
Subcellular localization
cytoplasm 540(24.8%)
nucleus 536(24.62%)
plasma membrane 349(16.03%)
extracellular 290(13.32%)
mitochondria 285(13.09%)
other 63(2.89%)
cytoplasm,nucleus 62(2.85%)
endoplasmic reticulum 52(2.39%)
B
U87 aggregations up-regulation proteins
U118 aggregations up-regulation proteins
745
159
989
U87 aggregations down-regulation proteins
U118 aggregations down-regulation proteins
1467
272
756
C
U87 GO term
signal transduction by p53 class mediator
regulation of stem cell proliferation
positive regulation of metabolic process
positive regulation of gene expression
Notch signaling pathway
mitotic G1 DNA damage checkpoint signaling
mitochondrial DNA repair
macroautophagy
cellular response to xenobiotic stimulus
cellular macromolecule biosynthetic process
cell motility
cell migration
cell growth
cell-cell signaling
basement membrane disassembly
mismatch repair complex
integrin alpha2-beta1 complex
transcription regulator activity
mismatched DNA binding
extracellular matrix binding
collagen receptor activity
cadherin binding
Biological Process
Molecular Function
Fold_Enrichment
Invasion
Stemness and therapy-resistance
Proliferation
Senescence
pvalue
Size
D
U87 Reactome pathway
R-HSA-913531 Interferon Signaling
R-HSA-8953854 Metabolism of RNA
R-HSA-8852135 Protein ubiquitination
R-HSA-877300 Interferon gamma signaling
R-HSA-75072 mRNA Editing
R-HSA-74160 Gene expression (Transcription)
R-HSA-6796648 TP53 Regulates Transcription of DNA Repair Genes
R-HSA-5673000 RAF activation
R-HSA-5654738 Signaling by FGFR2
R-HSA-5633007 Regulation of TP53 Activity
R-HSA-5632928 Defective Mismatch Repair Associated With MSH2
R-HSA-5423599 Diseases of Mismatch Repair (MMR)
R-HSA-5099900 WNT5A-dependent internalization of FZD4
R-HSA-4839726 Chromatin organization
R-HSA-425407 SLC-mediated transmembrane transport
R-HSA-3700989 Transcriptional Regulation by TP53
R-HSA-3247509 Chromatin modifying enzymes
R-HSA-3238698 WNT ligand biogenesis and trafficking
R-HSA-2219528 PI3K/AKT Signaling in Cancer
R-HSA-1852241 Organelle biogenesis and maintenance
R-HSA-176974 Unwinding of DNA
R-HSA-156827 L13a-mediated translational silencing of Ceruloplasmin expression
R-HSA-1430728 Metabolism
R-HSA-1226099 Signaling by FGFR in disease
Fold_Enrichment
Invasion
Stemness and therapy-resistance
Proliferation
Senescence
pvalue
Size

Sup 2

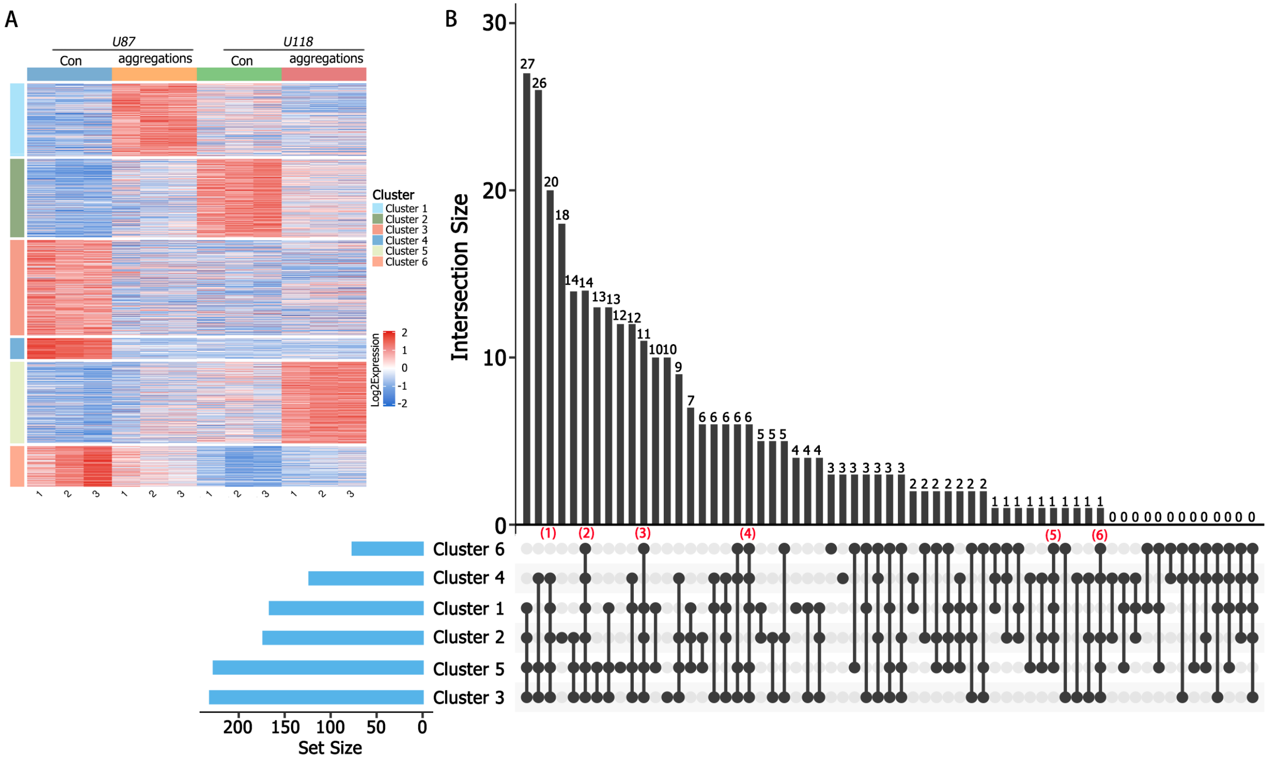


(1)+(2):

hsa04151 PI3K-Akt signaling pathway; hsa04210 Apoptosis; hsa05200 Pathways in cancer; hsa04510 Focal adhesion; hsa04014 Ras signaling pathway; hsa05206 MicroRNAs in cancer; MAPK signaling pathway; Proteoglycans in cancer; Phosphatidylinositol signaling system; Rap1 signaling pathway; Phospholipase D signaling pathway.............;

(2):

hsa04151 hsa05200 Pathways in cancer; hsa04510 Focal adhesion; hsa04014 Ras signaling pathway; hsa05206 MicroRNAs in cancer; MAPK signaling pathway; Phospholipase D signaling pathway.............;

(3)+(2):

hsa04151 hsa04210 Apoptosis; hsa05200 Pathways in cancer; hsa04510 Focal adhesion; hsa04014 Ras signaling pathway; hsa05206 MicroRNAs in cancer; MAPK signaling pathway; Phospholipase D signaling pathway.............;

(4)+(2) :

hsa04151 hsa05200 Pathways in cancer; hsa04510 Focal adhesion; hsa04014 Ras signaling pathway; hsa05206 MicroRNAs in cancer; MAPK signaling pathway; Phospholipase D signaling pathway; Cellular senescence.............;

(5)+(2) :

hsa04151 hsa05200 Pathways in cancer; hsa04510 Focal adhesion; hsa04014 Ras signaling pathway; hsa05206 MicroRNAs in cancer; MAPK signaling pathway; Phospholipase D signaling pathway.............;

(6)+(2) :

hsa04151 hsa05200 Pathways in cancer; hsa04510 Focal adhesion; hsa04014 Ras signaling pathway; hsa05206 MicroRNAs in cancer; MAPK signaling pathway; Phospholipase D signaling pathway.............;

Sup 3

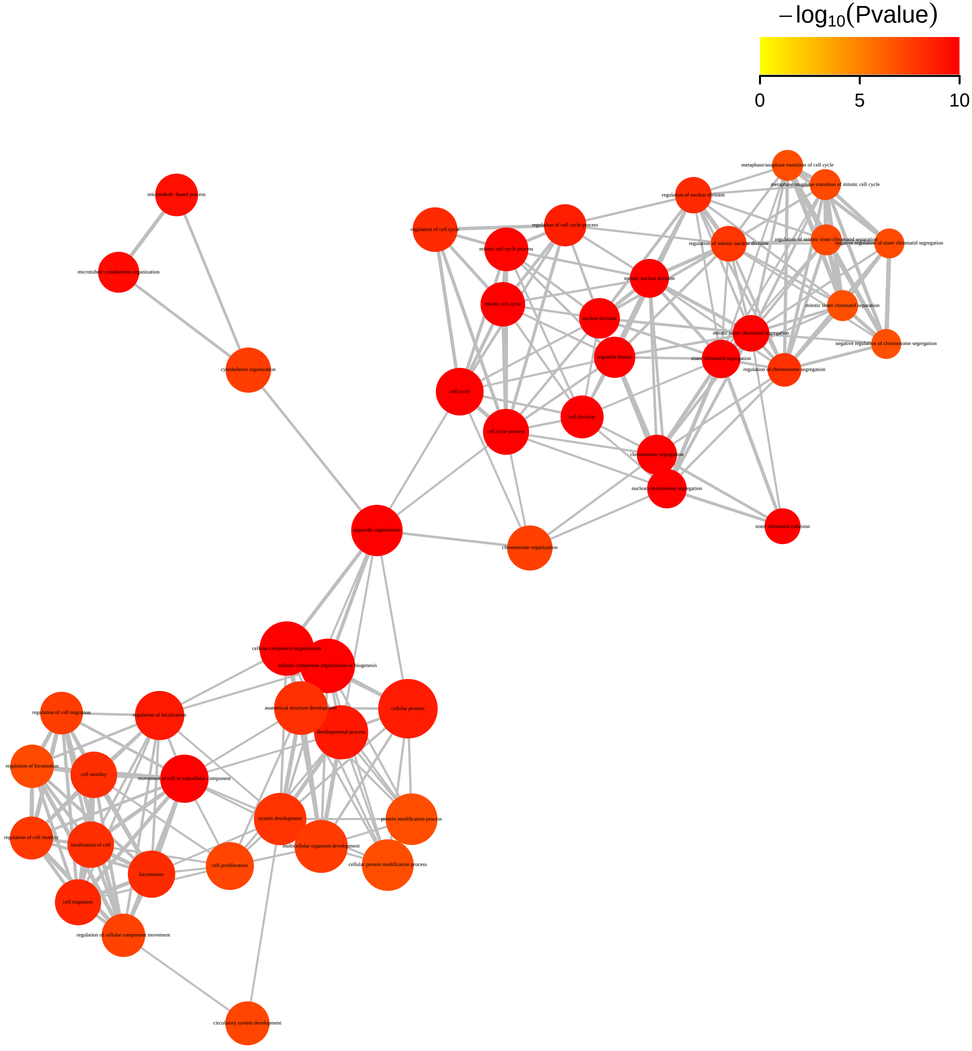

$-\log_{10}(\text{Pvalue})$
0
5
10
metaphase/anaphase transition of cell cycle
metaphase/anaphase transition of mitotic cell cycle
regulation of nuclear division
microtubule−based process
regulation of cell cycle process
regulation of cell cycle
regulation of mitotic sister chromatid separation
negative regulation of sister chromatid segregation
regulation of mitotic nuclear division
mitotic cell cycle process
microtubule cytoskeleton organization
mitotic nuclear division
mitotic cell cycle
nuclear division
mitotic sister chromatid separation
mitotic sister chromatid segregation
negative regulation of chromosome segregation
organelle fission
sister chromatid segregation
regulation of chromosome segregation
cytoskeleton organization
cell cycle
cell division
cell cycle process
chromosome segregation
nuclear chromosome segregation
sister chromatid cohesion
organelle organization
chromosome organization
cellular component organization
cellular component organization or biogenesis
regulation of cell migration
regulation of localization
anatomical structure development
cellular process
developmental process
regulation of locomotion
cell motility
movement of cell or subcellular component
system development
protein modification process
regulation of cell motility
localization of cell
multicellular organism development
locomotion
cell proliferation
cellular protein modification process
cell migration
regulation of cellular component movement
circulatory system development

Sup 4

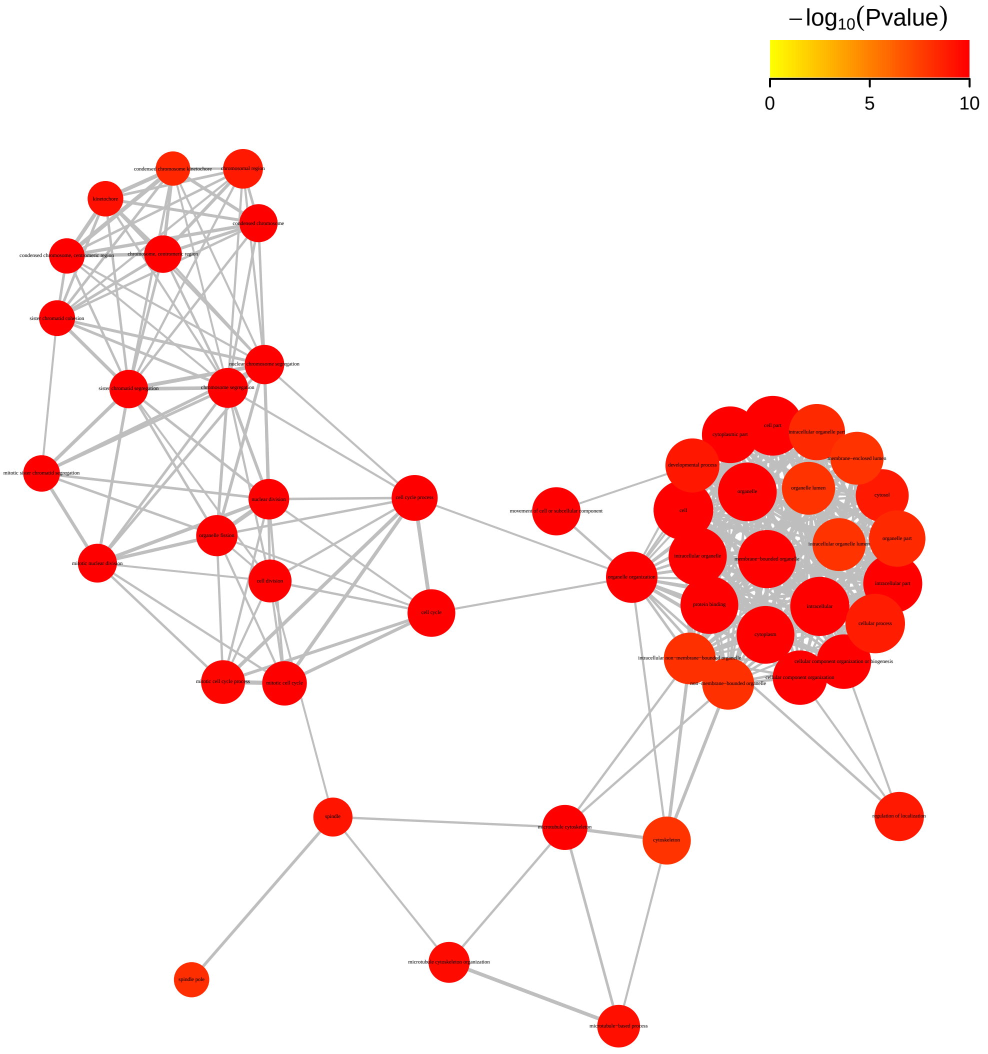

$-\log_{10}(\text{Pvalue})$
0
5
10
condensed chromosome kinetochore
chromosomal region
kinetochore
condensed chromosome
condensed chromosome, centromeric region
chromosome, centromeric region
sister chromatid cohesion
nuclear chromosome segregation
sister chromatid segregation
chromosome segregation
mitotic sister chromatid segregation
nuclear division
organelle fission
mitotic nuclear division
cell division
cell cycle process
cell cycle
mitotic cell cycle process
mitotic cell cycle
movement of cell or subcellular component
organelle organization
cytoplasmic part
cell part
intracellular organelle part
membrane-enclosed lumen
developmental process
organelle
organelle lumen
cytosol
cell
intracellular organelle lumen
organelle part
intracellular organelle
membrane-bounded organelle
intracellular part
protein binding
intracellular
cellular process
cytoplasm
intracellular non-membrane-bounded organelle
cellular component organization or biogenesis
cellular component organization
non-membrane-bounded organelle
spindle
microtubule cytoskeleton
cytoskeleton
regulation of localization
spindle pole
microtubule cytoskeleton organization
microtubule-based process

# Sup 5

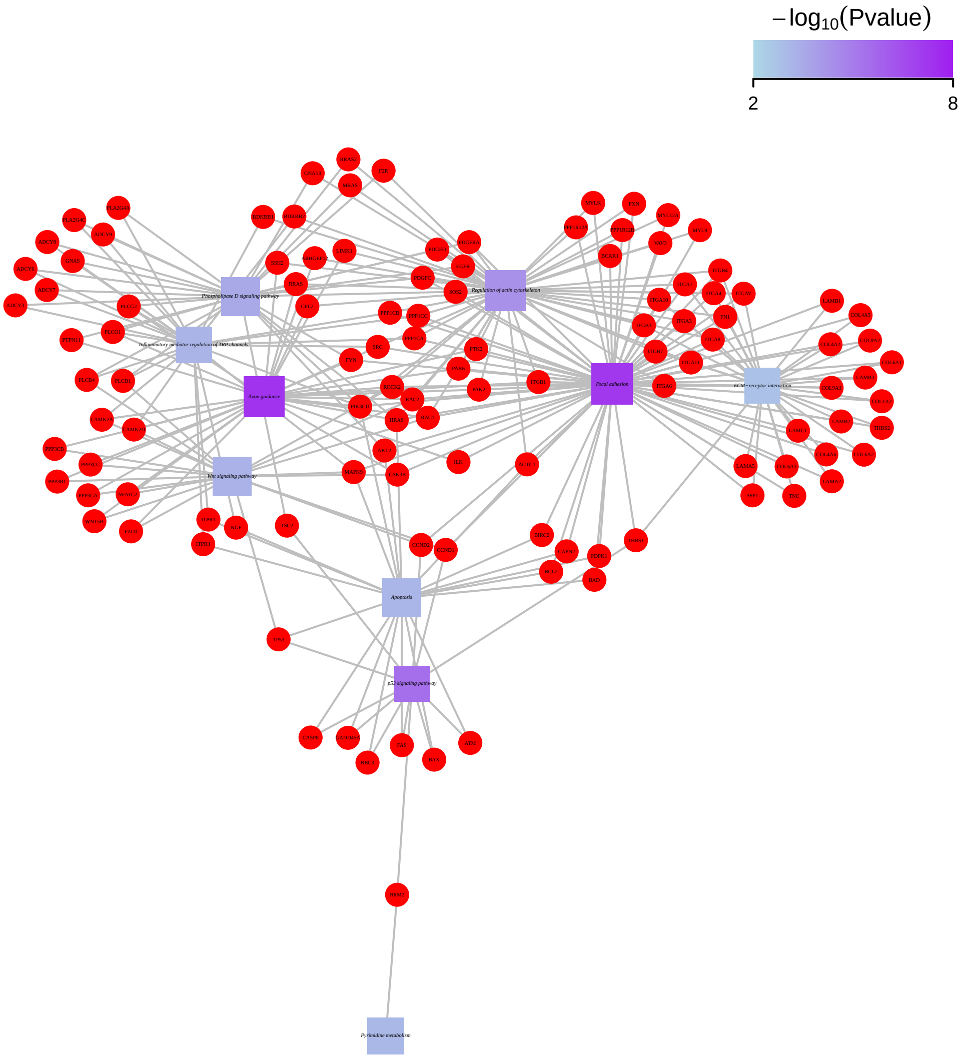
−log10(Pvalue)
2
8
Phospholipase D signaling pathway
Regulation of actin cytoskeleton
Inflammatory mediator regulation of TRP channels
Axon guidance
Focal adhesion
ECM−receptor interaction
Wnt signaling pathway
Apoptosis
p53 signaling pathway
Pyrimidine metabolism

Sup 6

A

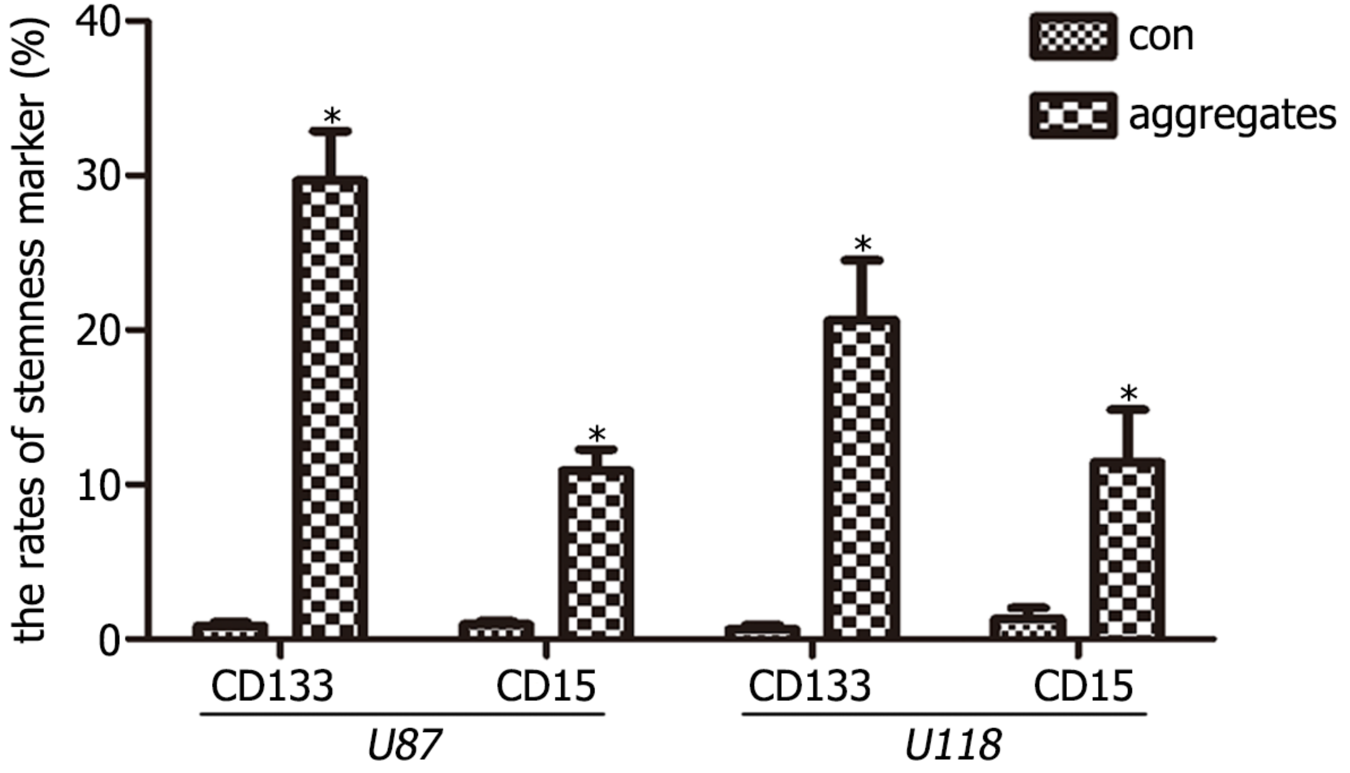


B

U118 aggregates

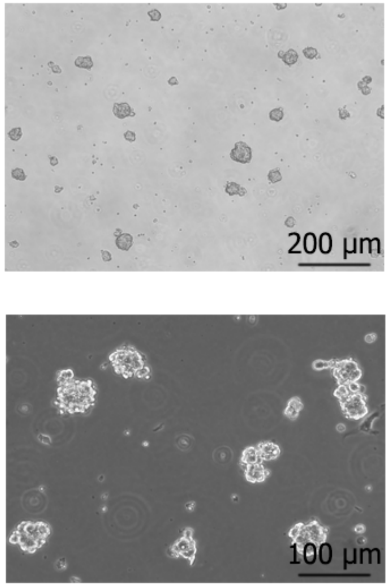

# Sup 7

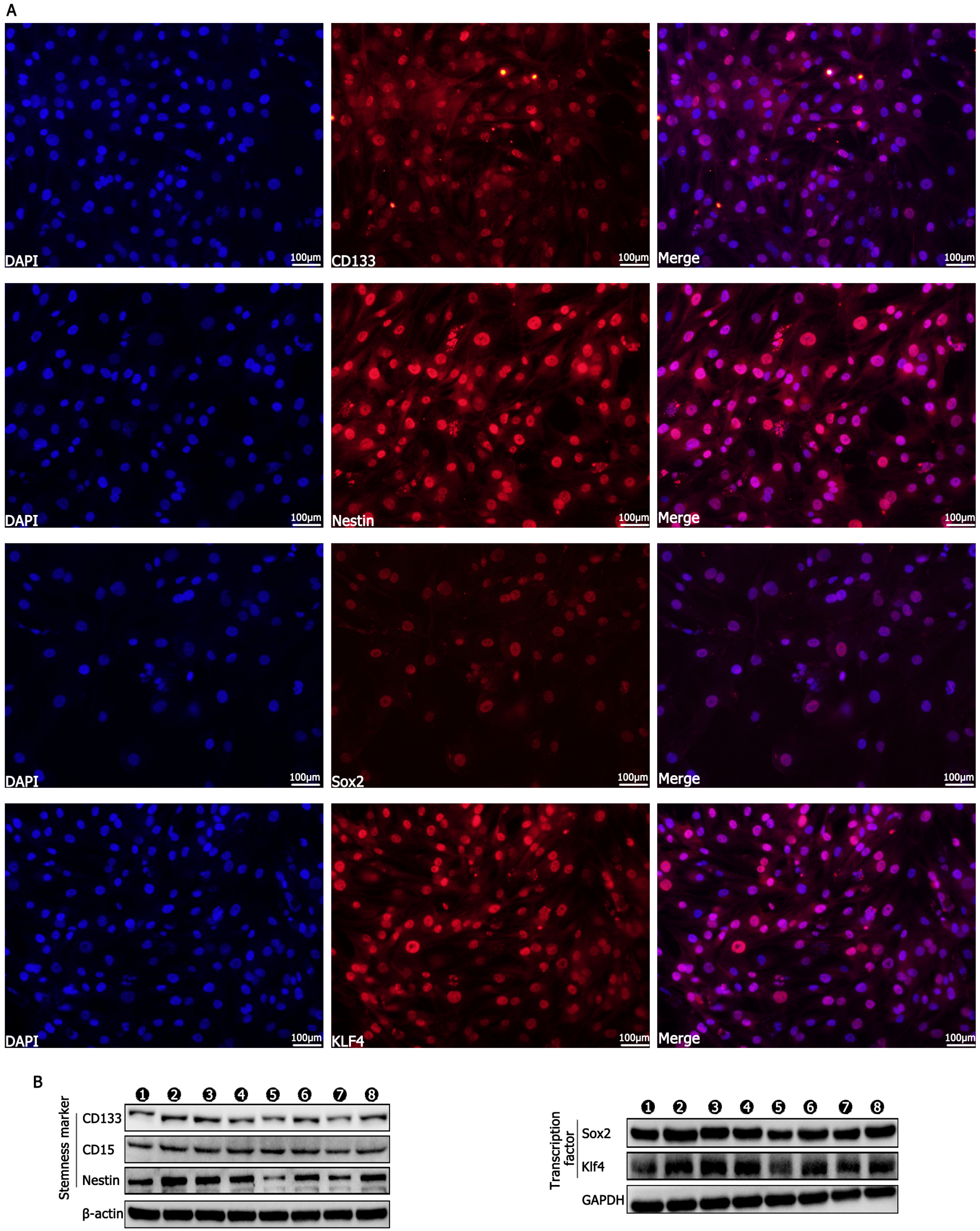

A
DAPI
CD133
Merge
Nestin
Sox2
KLF4
100μm
B
Stemness marker
CD133
CD15
Nestin
β-actin
Transcription factor
Sox2
Klf4
GAPDH
❶ ❷ ❸ ❹ ❺ ❻ ❼ ❽

Sup 8

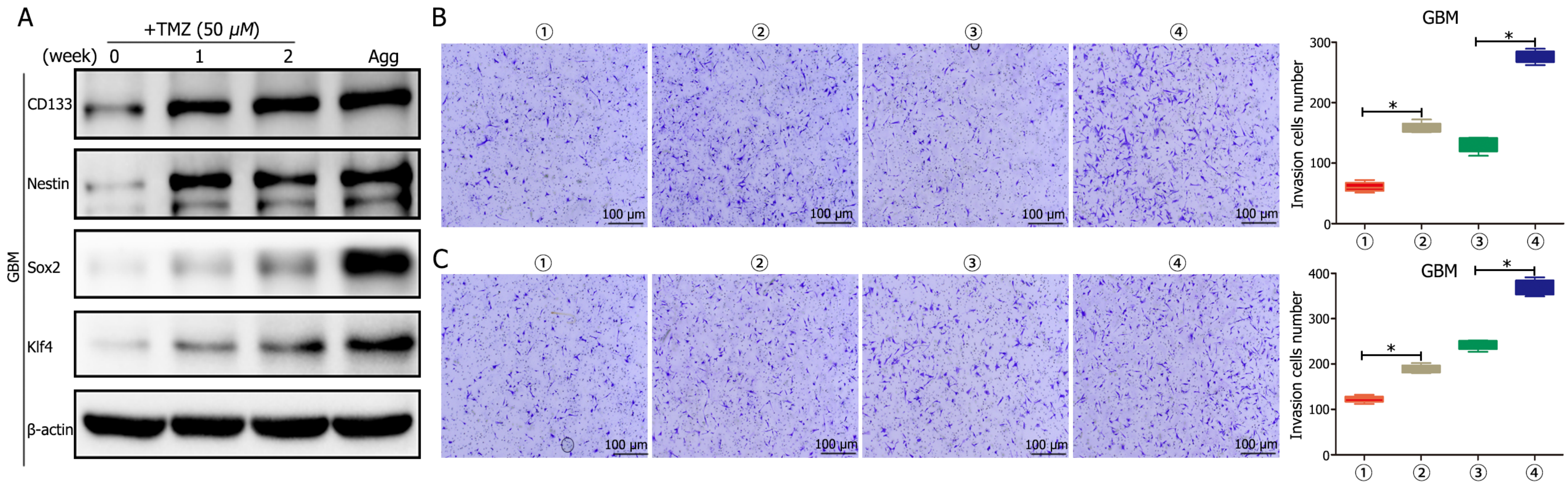

A
+TMZ (50 μM)
(week) 0 1 2 Agg
GBM
CD133
Nestin
Sox2
Klf4
β-actin
B
①
②
③
④
100 μm
GBM
Invasion cells number
300
200
100
0
*
C
400

Sup 9

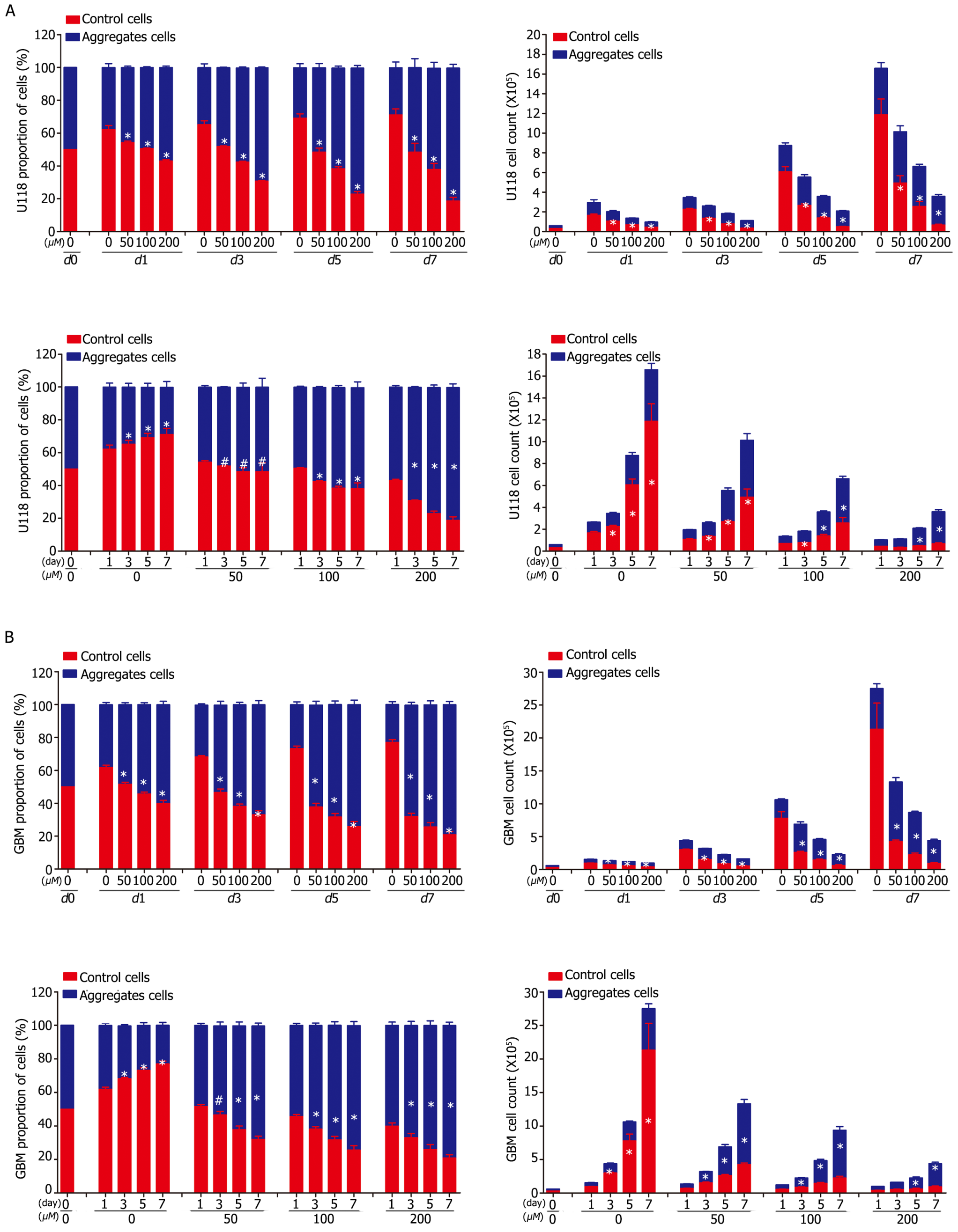

A
Control cells
Aggregates cells
U118 proportion of cells (%)
U118 cell count (X10⁵)
(μM)
(day)
d0
d1
d3
d5
d7
B
GBM proportion of cells (%)
GBM cell count (X10⁵)

Sup 10

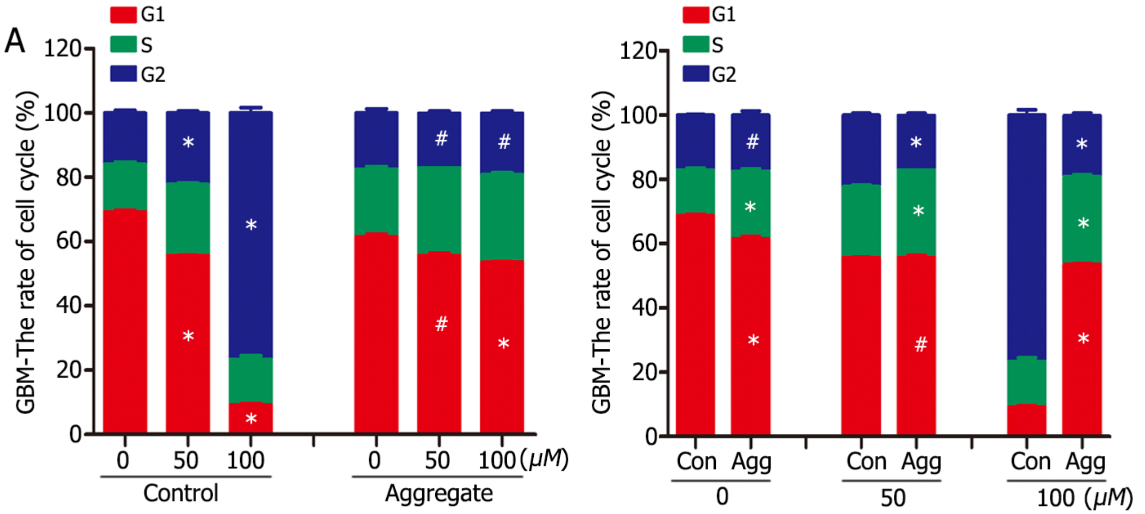

A
G1
S
G2
GBM-The rate of cell cycle (%)
0
50
100
0
50
100 (μM)
Control
Aggregate
Con
Agg
100 (μM)
B
Apoptosis
Necrosis
GBM-Cells death (%)
(μM) 50

Sup 11

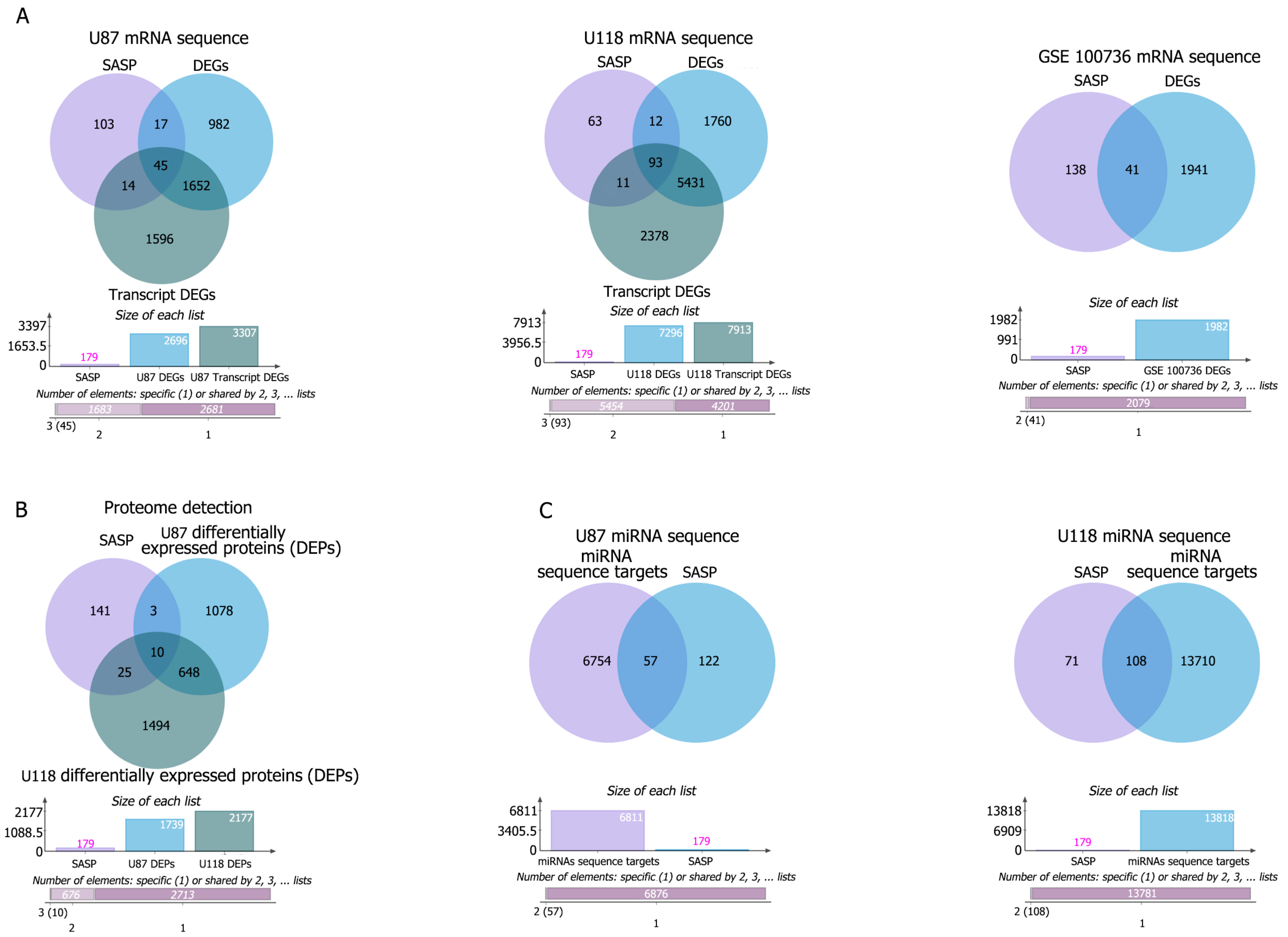
A
U87 mRNA sequence
SASP
DEGs
103
17
982
45
14
1652
1596
Transcript DEGs
Size of each list
3397
1653.5
0
179
2696
3307
SASP
U87 DEGs
U87 Transcript DEGs
Number of elements: specific (1) or shared by 2, 3, ... lists
1683
2681
3 (45)
2
1
U118 mRNA sequence
SASP
DEGs
63
12
1760
93
11
5431
2378
Transcript DEGs
Size of each list
7913
3956.5
0
179
7296
7913
SASP
U118 DEGs
U118 Transcript DEGs
Number of elements: specific (1) or shared by 2, 3, ... lists
5454
4201
3 (93)
2
1
GSE 100736 mRNA sequence
SASP
DEGs
138
41
1941
Size of each list
1982
991
0
179
1982
SASP
GSE 100736 DEGs
Number of elements: specific (1) or shared by 2, 3, ... lists
2079
2 (41)
1
B
Proteome detection
SASP
U87 differentially expressed proteins (DEPs)
141
3
1078
10
25
648
1494
U118 differentially expressed proteins (DEPs)
Size of each list
2177
1088.5
0
179
1739
2177
SASP
U87 DEPs
U118 DEPs
Number of elements: specific (1) or shared by 2, 3, ... lists
676
2713
3 (10)
2
1
C
U87 miRNA sequence
miRNA sequence targets
SASP
6754
57
122
Size of each list
6811
3405.5
0
6811
179
miRNAs sequence targets
SASP
Number of elements: specific (1) or shared by 2, 3, ... lists
6876
2 (57)
1
U118 miRNA sequence
SASP
miRNA sequence targets
71
108
13710
Size of each list
13818
6909
0
179
13818
SASP
miRNAs sequence targets
Number of elements: specific (1) or shared by 2, 3, ... lists
13781
2 (108)
1

# Sup 12

A

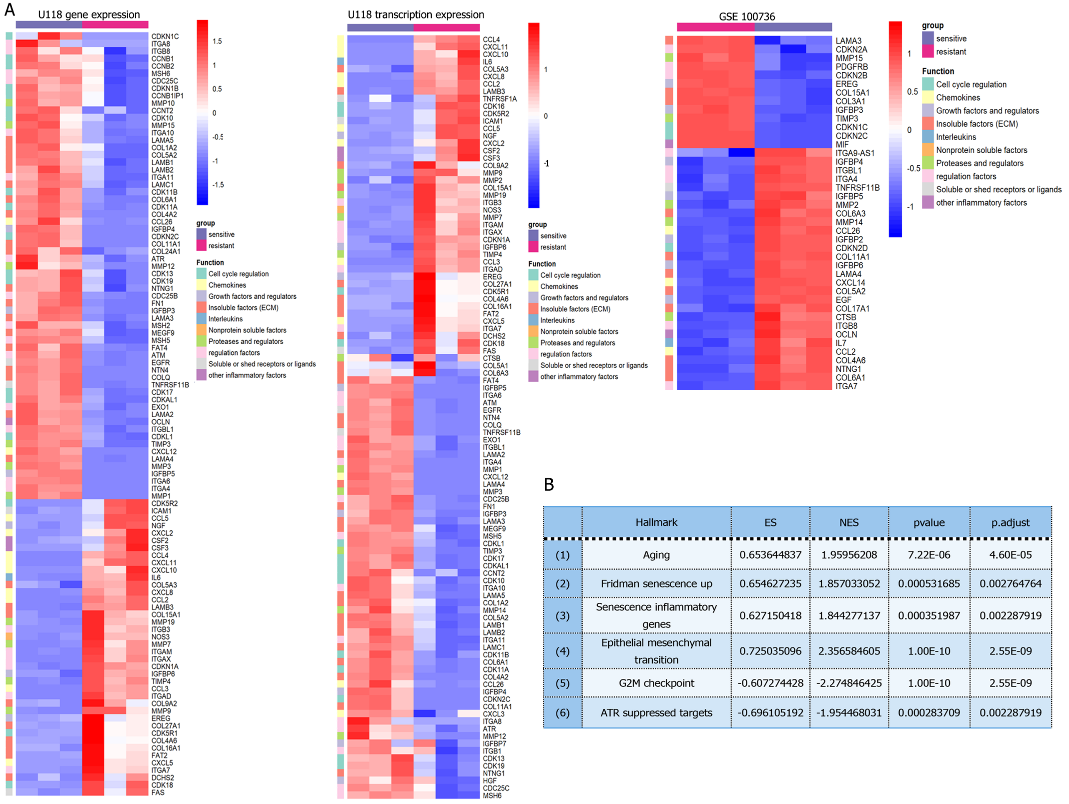


B

| | Hallmark | ES | NES | pvalue | p.adjust |
|---|---|---|---|---|---|
| (1) | Aging | 0.653644837 | 1.95956208 | 7.22E-06 | 4.60E-05 |
| (2) | Fridman senescence up | 0.654627235 | 1.857033052 | 0.000531685 | 0.002764764 |
| (3) | Senescence inflammatory genes | 0.627150418 | 1.844277137 | 0.000351987 | 0.002287919 |
| (4) | Epithelial mesenchymal transition | 0.725035096 | 2.356584605 | 1.00E-10 | 2.55E-09 |
| (5) | G2M checkpoint | -0.607274428 | -2.274846425 | 1.00E-10 | 2.55E-09 |
| (6) | ATR suppressed targets | -0.696105192 | -1.954468031 | 0.000283709 | 0.002287919 |

C

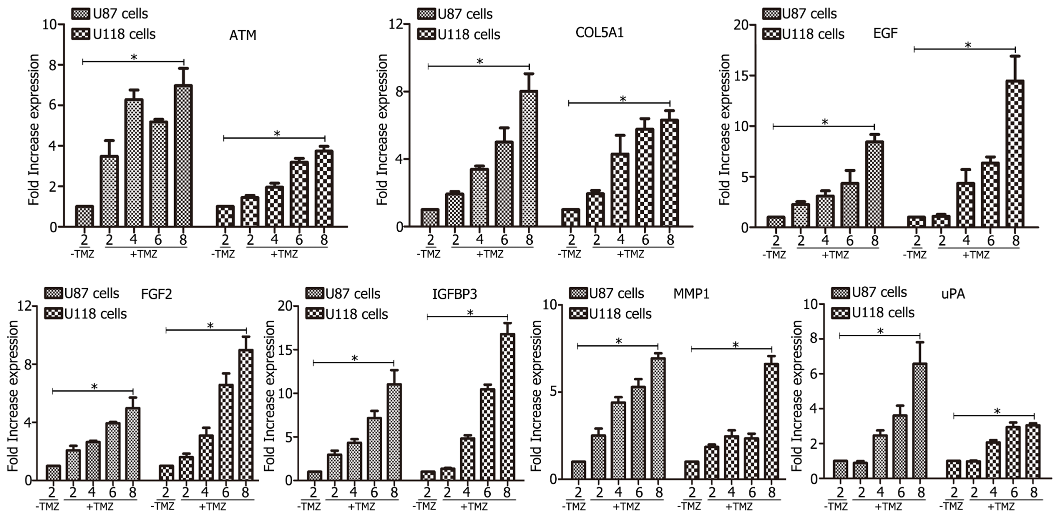

Sup 13

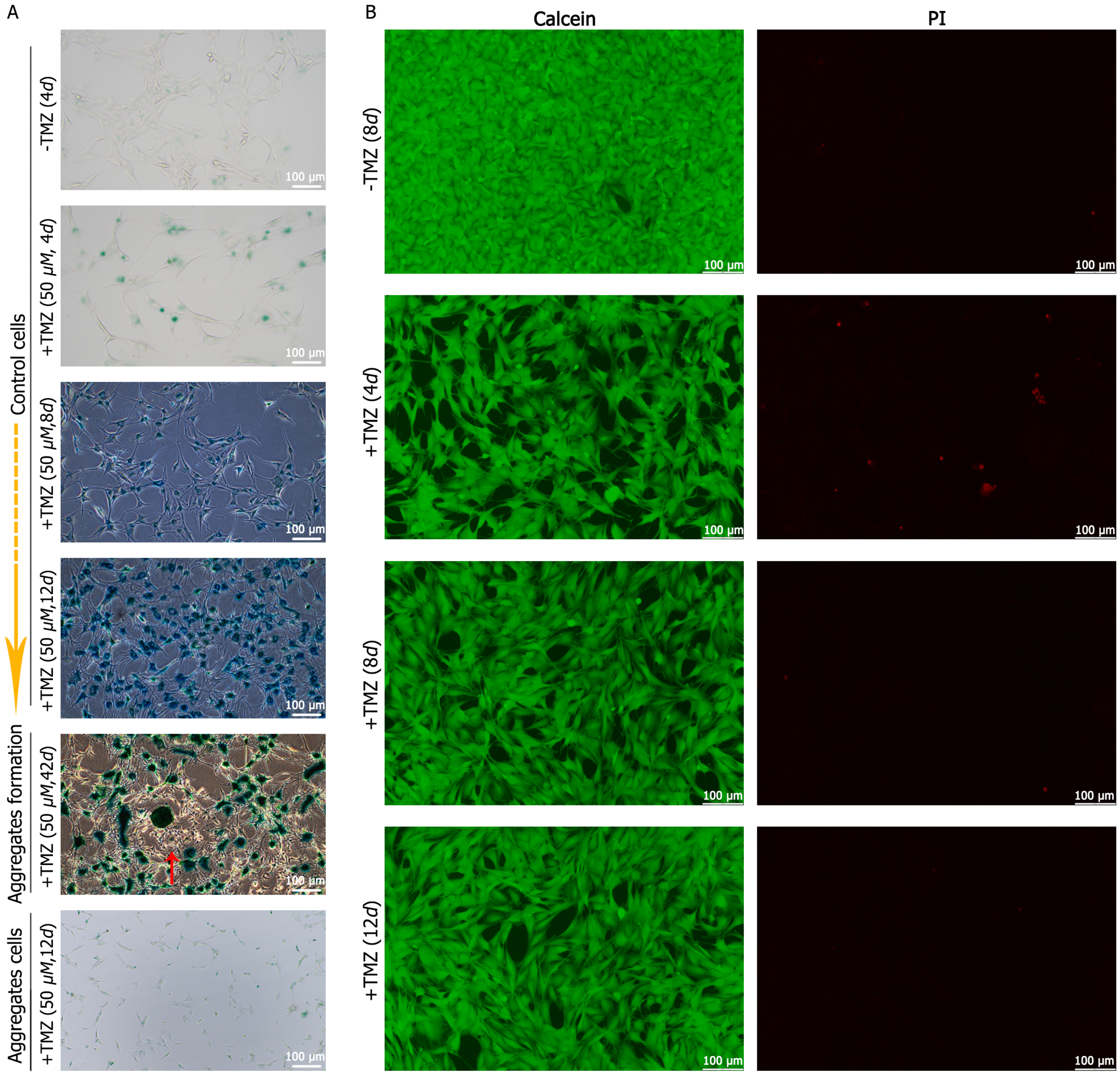
A
Control cells
Aggregates formation
Aggregates cells
-TMZ (4d)
+TMZ (50 μM, 4d)
+TMZ (50 μM,8d)
+TMZ (50 μM,12d)
+TMZ (50 μM,42d)
+TMZ (50 μM,12d)
100 μm
B
Calcein
PI
-TMZ (8d)
+TMZ (4d)
+TMZ (8d)
+TMZ (12d)
100 μm

Sup 14

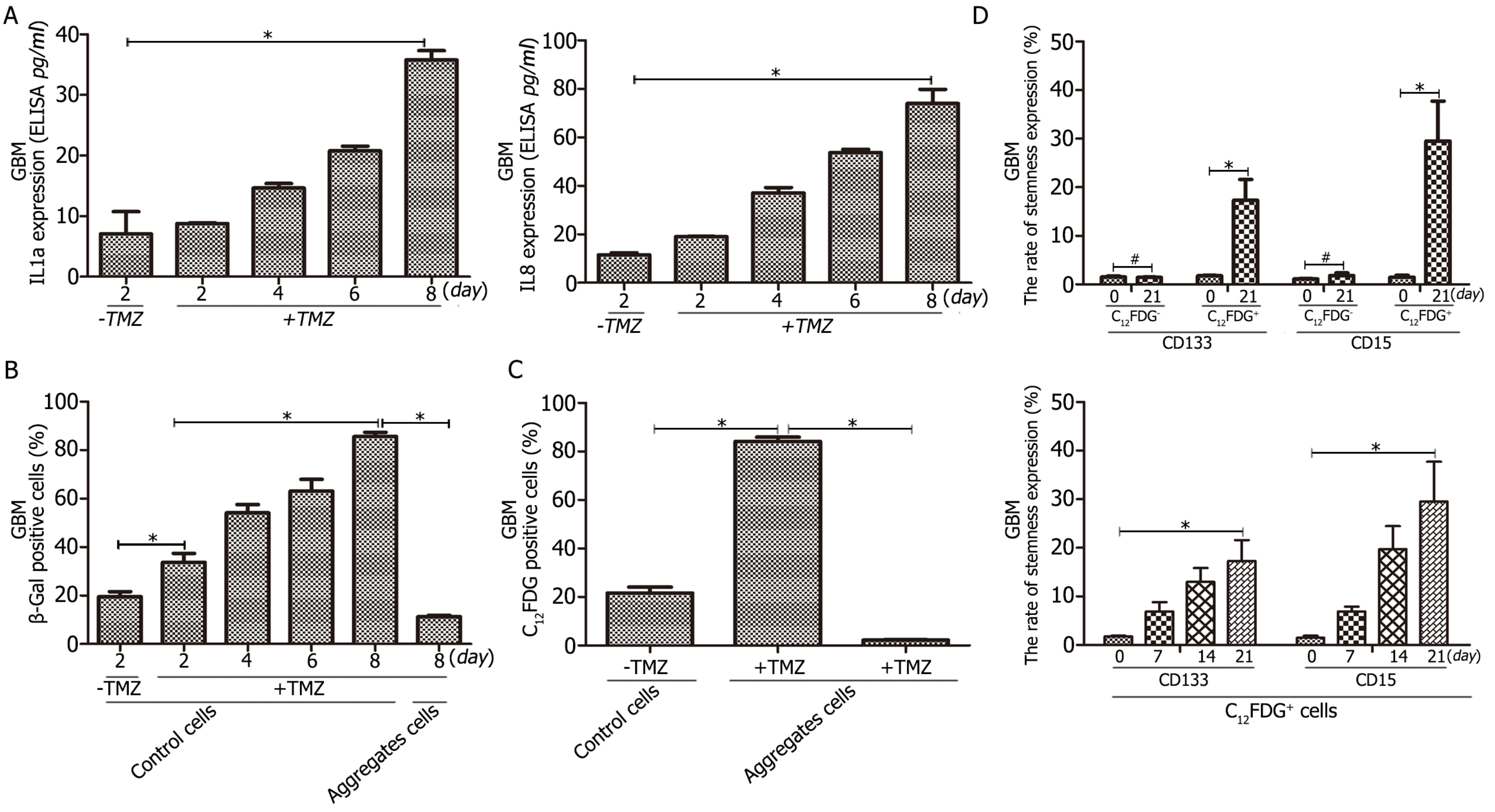

A
GBM
IL1a expression (ELISA pg/ml)
-TMZ
+TMZ
(day)
GBM
IL8 expression (ELISA pg/ml)
B
GBM
β-Gal positive cells (%)
Control cells
Aggregates cells
C
GBM
C12FDG positive cells (%)
D
GBM
The rate of stemness expression (%)
CD133
CD15
C12FDG+ cells

Sup 15

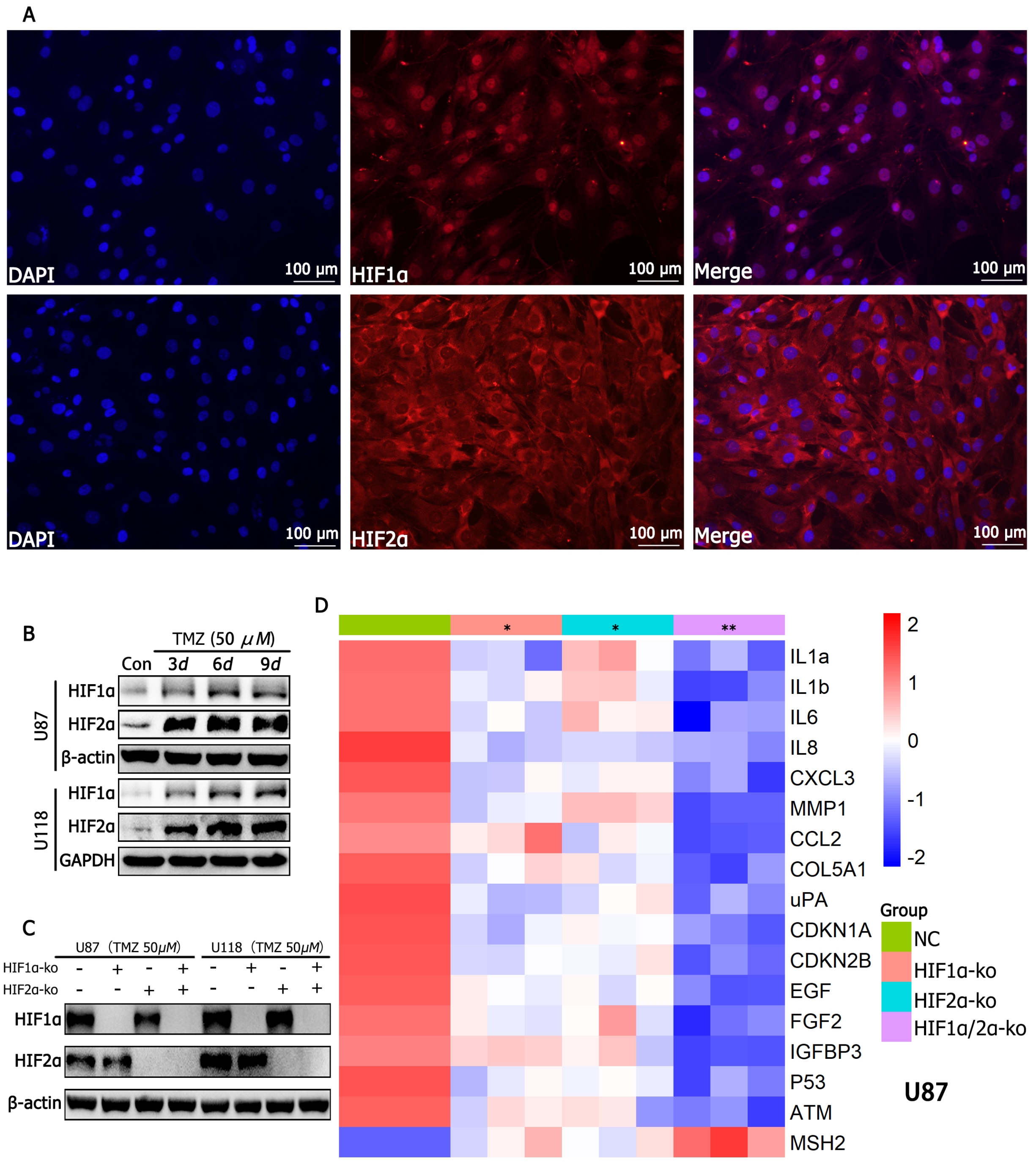
A
DAPI
100 μm
HIF1α
100 μm
Merge
100 μm
DAPI
100 μm
HIF2α
100 μm
Merge
100 μm
B
TMZ (50 μM)
Con
3d
6d
9d
U87
HIF1α
HIF2α
β-actin
U118
HIF1α
HIF2α
GAPDH
C
U87 (TMZ 50μM)
U118 (TMZ 50μM)
HIF1α-ko - + - + - + - +
HIF2α-ko - - + + - - + +
HIF1α
HIF2α
β-actin
D
*
*
**
IL1a
IL1b
IL6
IL8
CXCL3
MMP1
CCL2
COL5A1
uPA
CDKN1A
CDKN2B
EGF
FGF2
IGFBP3
P53
ATM
MSH2
2
1
0
-1
-2
Group
NC
HIF1α-ko
HIF2α-ko
HIF1α/2α-ko
U87

Sup 16

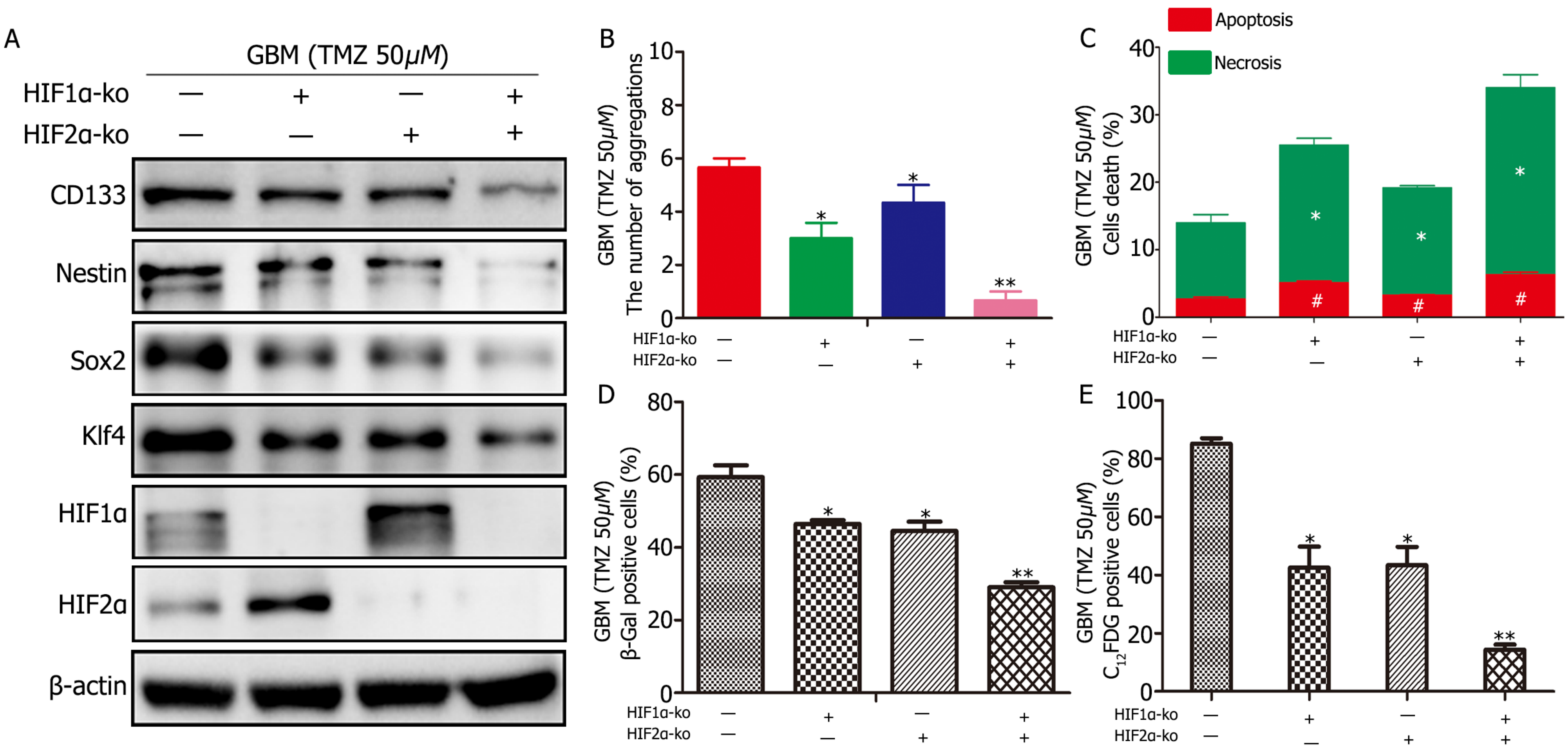

A
GBM (TMZ 50μM)
HIF1a-ko — + — +
HIF2a-ko — — + +
CD133
Nestin
Sox2
Klf4
HIF1a
HIF2a
β-actin
B
GBM (TMZ 50μM)
The number of aggregations
C
Apoptosis
Necrosis
GBM (TMZ 50μM)
Cells death (%)
D
GBM (TMZ 50μM)
β-Gal positive cells (%)
E
GBM (TMZ 50μM)
C12FDG positive cells (%)

**Supplementary Figure Legends**

**Supplementary Figure 1 Mass spectrometric detection showed factors associated with senescence, stemness, invasion, and chemotherapy resistance were activated. A** The localization of differential proteins between control and aggregations were mainly in the cytoplasm, followed by the nucleus, plasma membrane, mitochondria, and extracellular. **B** There were 159 overlapping up-regulated proteins and 272 overlapping down-regulated proteins between U87 and U118 differential proteins. **C** GO analysis presented stemness, invasion, chemotherapy resistance, and senescence-associated terms that had significant differences in the TMZ treatment group compared with control groups, including extracellular matrix, regulation of stem cell proliferation, Notch signaling pathway, cell migration, mismatch repair complex, integrin alpha2-beta1 complex, cell motility, and others. **D** Reactome pathway analysis showed that Transcriptional Regulation by TP53, Diseases of Mismatch Repair (MMR), Defective mismatch repair associated with MSH2, and others were activated.

**Supplementary Figure 2 UpSet plot analysis from the mass spectrometric detection A** Differential proteins were divided into six clusters according to the trend of protein expression. **B** The results from the UpSet plot showed that each cluster had the same activating pathways as other clusters, such as cellular senescence, focal adhesion, HIF-1 signaling pathway, mismatch repair, signaling pathways regulating pluripotency of stem cells, cell cycle, MAPK signaling pathway, and others, and all of them were activated.

**Supplementary Figure 3 Biological process enrichment map analysis** The biological process enrichment map showed cell senescence, cell cycle, and invasion-associated terms such as chromosome segregation, cellular component organization, organelle organization, cell cycle, metabolic process, and cell migration had significant correlations with each other.

**Supplementary Figure 4 Gene ontology enrichment map analysis** The whole gene ontology enrichment map showed cell senescence, cell cycle, and invasion-associated terms such as condensed chromosome, cell cycle process, cell division, intracellular organelle formation, and others were activated and had significant correlations with each other.

**Supplementary Figure 5 Significance intersection of KEGG pathway enrichment and different expression proteins correspondingly** The network of significance intersection of KEGG enrichment between mRNA and miRNAs showed invasion-associated pathways, including focal adhesion and ECM-receptor interaction, were activated due to the activation of collagen and laminin genes. In addition, the Phospholipase D signaling pathway, marking hypermetabolism and cellular senescence, was activated by ADCY family proteins. The Wnt signaling pathway and p53

signaling pathway regulated cellular senescence, stemness, cell cycle progression, and invasion, which were also activated due to the differential expression of TP53, CCND1, CCND2, FAS, and ATM.

**Supplementary Figure 6 Stemness markers detection of aggregation cells A** Flow cytometry demonstrated higher levels of CD133 and CD15 in the newly formed aggregations than in control. **B** Aggregations were cultured in stem cell medium (DMEM/F12+EGF+FGF2+B27), and they grew in suspension with stem cell morphology. $^{*}P<0.05$ was determined using Student's t test.

**Supplementary Figure 7 Stemness and transcription factors detection of aggregation cells under TMZ treatment A** CD133, Nestin, Sox2, and Klf4 were highly expressed after $CD133^{-}CD15^{-}$ cells were exposed to TMZ for two weeks. **B** Tumor tissues had elevated expression of CD133, CD15, Nestin, Sox2, and Klf4 in mice implanted with $CD133^{-}CD15^{-}$ cells treated with TMZ compared to controls without TMZ treatment.

**Supplementary Figure 8 Aggregations exhibited enhanced stemness and invasion A** The expression of CD133, CD15, Nestin, Sox2, and Klf4 was increased after one to two weeks of TMZ treatment, and higher levels in aggregation cells were observed. **B-C** Aggregation cells demonstrated greater invasive capacity compared to controls. $^{*}P<0.05$ was determined using Student's t test.

**Supplementary Figure 9 Aggregation cells presented lower proliferation rate but higher chemotherapy resistance** Without TMZ treatment for one day, the proportion of U118 and GBM aggregation cells was around 40% of the whole cell mass, and the proportion of aggregation cells decreased after being cultured for 7 days, and the number of aggregation cells was lower compared to control cells. After TMZ treatment, the results presented a higher proportion of aggregation cells in the cell mass, and the tendency became more significant with longer exposure time or higher TMZ concentration, and the trends of cell count assays showed the same results. The biggest statistical difference between the two groups was when cells were cultured with 200 μM TMZ for 7 days, and the proportion of aggregation cells reached almost 80%, while the proportion of control cells was less than 20%. $^{*}P<0.05$ were determined using one-way ANOVA or Student's t test and $^{\#}P>0.05$ were determined using Student's t test.

**Supplementary Figure 10 Aggregation cells presented higher chemotherapy resistance A** Control cells predominantly occupied at G1 phase, while aggregation cells were in the S state. TMZ (50 μM) arrested control cells in the G2 phase, with an increased G2 proportion at 100 μM. Aggregation cells displayed no significant change in the cell cycle proportion with increasing TMZ concentration. **B**: Aggregation cells exhibited lower apoptosis and necrosis rates compared

to control cells under the same TMZ concentrations. $^{*}P<0.05$ and $^{\#}P>0.05$ were determined using Student’s t test.

**Supplementary Figure 11 Venn diagram of differential expression genes according to proteome, mRNA, and miRNA sequence analysis A** Venn diagram among SASP, mRNA DEGs, and mRNA transcript DEGs. **B** Venn diagram among SASP, U87 differentially expressed proteins, and U118 differentially expressed proteins. **C** Venn diagram between SASP and U87 or U118 miRNA sequence target genes.

**Supplementary Figure 12 SASP detection according to mRNA sequence and RT-qPCR A** The heatmap of overlapping DEGs in U118 and GSE 100736 with SASP in mRNA levels showed that most DEGs were up-regulated, such as IL6, IL7, CXCL3, CXCL2, ICAM1, CCL2, CCL3, MMP7, and TIMP1, while some senescence-associated inhibiting genes such as CDC25B, CDC25C, CDC25A, CDKN2D, MSH6, MSH5, and MSH6 were down-regulated. **B** Parts of GSEA analysis of the hallmark in U87 for Figure 5A. **C** RT-qPCR showed SASP genes such as ATM, COL5A1, EGF, FGF2, IGFBP3, MMP1, and uPA were significantly increased after TMZ treatment in a time-dependent manner. $^{*}P<0.05$ was determined using Student’s t test.

**Supplementary Figure 13 Morphological examination of cellular senescence of $CD133^{-}CD15^{-}$ GBM cells under TMZ treatment A** The morphological examination showed $CD133^{-}CD15^{-}$ GBM cells became hypertrophic and flattened with SA-β-Gal high expression under TMZ treatment from day 4 to day 12, and after culturing for 2 months, newly formed aggregation cells appeared without features of cellular senescence. **B** $CD133^{-}CD15^{-}$ GBM cells were marked by calcein but showed low expression of PI.

**Supplementary Figure 14 SASP detection of primary GBM cells under TMZ treatment A** ELISA demonstrated an increase expression of IL1a, and IL8 in a time-dependent manner following TMZ treatment. **B** A significant increase of β-Gal-positive cells after TMZ treatment, and the rate of β-Gal-positive cells was lower in aggregation cells compared to control cells under TMZ treatment. **C** $C_{12}FDG$ expression increased after one week of TMZ treatment in $CD133^{-}CD15^{-}$ GBM cells, and there were lower levels of $C_{12}FDG$ in aggregation cells under equivalent TMZ concentrations. **D** Higher levels of CD133 and CD15 in $C_{12}FDG$-positive cells after culturing under TMZ for 21 days, while CD133 and CD15 expression was not change in $C_{12}FDG$-negative cells. $^{*}P<0.05$ were determined using one-way ANOVA or Student’s t test.

**Supplementary Figure 15 Cellular senescence and the formation of aggregation cells under TMZ treatment were regulated via HIF1α/HIF2α. A** $CD133^{-}CD15^{-}$ GBM cells were detected highly expressed HIF1α and HIF2α after exposure to TMZ for two weeks. **B** Western-blot

suggested CD133$^-$CD15$^-$ GBM cells under TMZ treatment for 3, 6, and 9 days had high expression of HIF1α and HIF2α. **C** Western-blot demonstrated successful knockout of HIF1α and HIF2α in CD133$^-$CD15$^-$ GBM cells. **D** RT-qPCR showed the lowest expression of SASP, including IL1a, IL1b, IL6, IL8, CCL2, and others after simultaneous HIF1α and HIF2α knockout, and solely HIF1α or HIF2α knockout cells had lower expression of SASP than control in U87 CD133$^-$CD15$^-$ cells.

**Supplementary Figure 16 HIF1α/HIF2α regulated cellular senescence and the formation of aggregation cells under TMZ treatment in primary GBM cells. A** Primary CD133$^-$CD15$^-$ GBM cells were knocked-out HIF1α and HIF2α successfully and stemness markers expression were decreased under the same TMZ concentrations compared to control cells. **B** Aggregation formation was significantly decreased in single HIF1α or HIF2α knockout primary CD133$^-$CD15$^-$ GBM cells, and the lowest formation of aggregation was after simultaneously knockout HIF1α and HIF2α**. C** There were most significantly increased apoptosis and necrosis rates in cells with simultaneous HIF1α and HIF2α knockouts, followed by single knockouts, compared to the control. **D-E** β-Gal-positive and $C_{12}$FDG-positive cells proportion decreased most significantly in simultaneous HIF1α and HIF2α knockout primary CD133$^-$CD15$^-$ GBM cells, with intermediate decreases in single knockouts compared to the control. $^*P<0.05$, $^{**}P<0.01$, and $^{\#}P>0.05$ were determined using Student's t test.

**Table S1** Differentiation protein expression involved in invasion and stemness
**Table S2** GO terms analysis according to differential protein expression of mass spectrometry
**Table S3** Reactome pathway analysis according to differential protein expression of mass spectrometry
**Table S4** KEGG pathway analysis according to differential protein expression of mass spectrometry
**Table S5** GO terms analysis according to differential gene expression of mRNA sequence
**Table S6** KEGG pathway analysis according to differential gene expression of mRNA sequence
**Table S7** GO terms analysis according to differential gene expression of miRNA sequence
**Table S8** KEGG pathway analysis according to differential gene expression of miRNA sequence
**Table S9** GO terms analysis according to differential gene expression of mRNA and miRNA sequence
**Table S10** KEGG pathway analysis according to differential gene expression of mRNA and miRNA sequence
**Table S11** GSEA analysis of the hallmark according to differential gene expression
**Table S12** Common elements of differential gene expression with SASP expression according to proteome, mRNA, and miRNA sequence analysis
**Table S13** Primary antibodies used for immunofluorescence staining

**Table S14** Primary antibodies used for western blotting

**Table S15** Sequences of primers used for RT–qPCR analysis

**Table S16** Sequences of sgRNA for knockout of HIF1α and HIF2α
