# Supplementary material for: Temozolomide promotes glioblastoma stemness expression through senescence-associated reprogramming via HIF1α/HIF2α regulation
Source: Cell Death Dis. 2025 Apr 19;16(1):317. doi: 10.1038/s41419-025-07617-w (PMC12009364; doi:10.1038/s41419-025-07617-w)

Figure 3 WB Raw data

U87 Raw data of WB

CD133

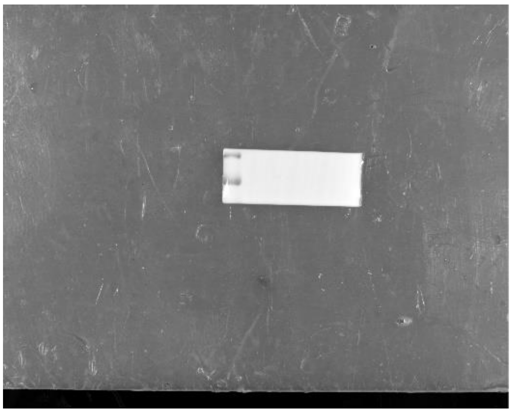

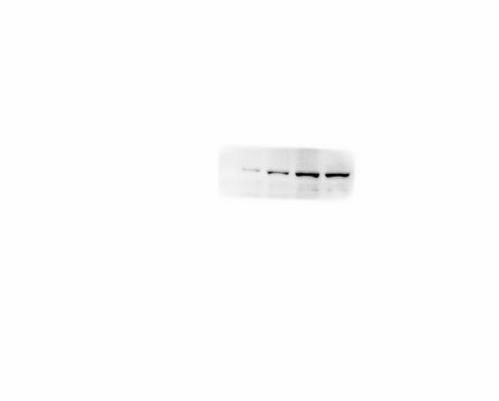

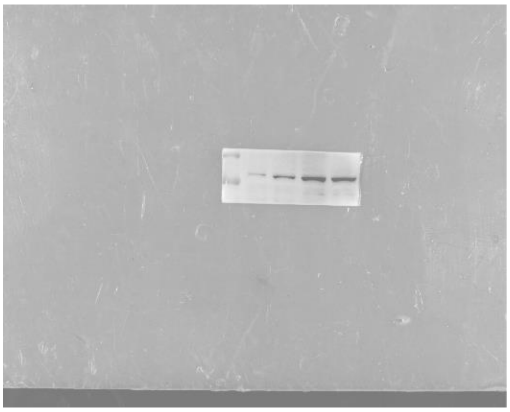

CD15

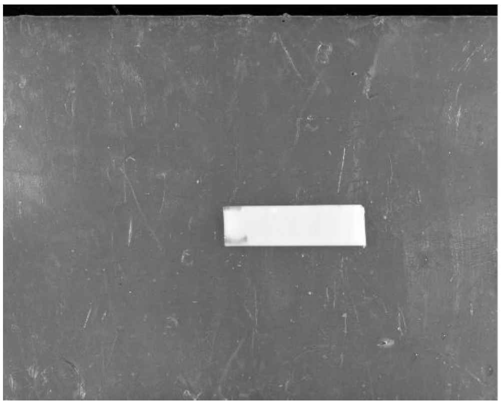

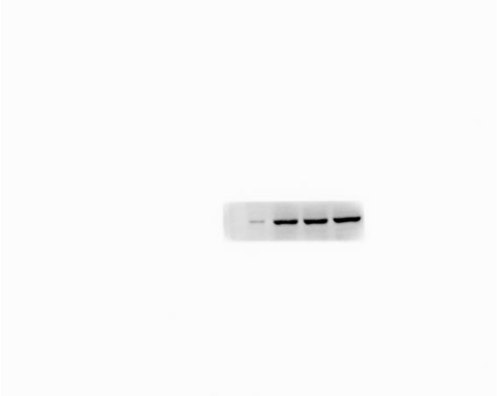

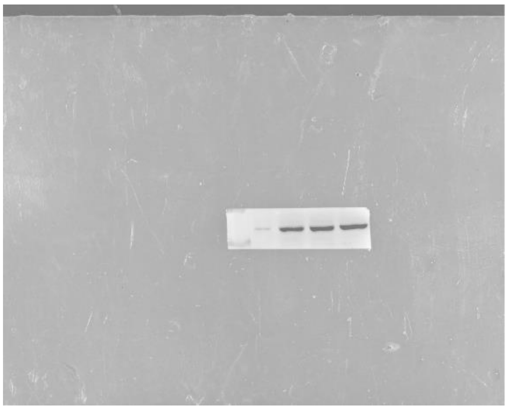

Nestin

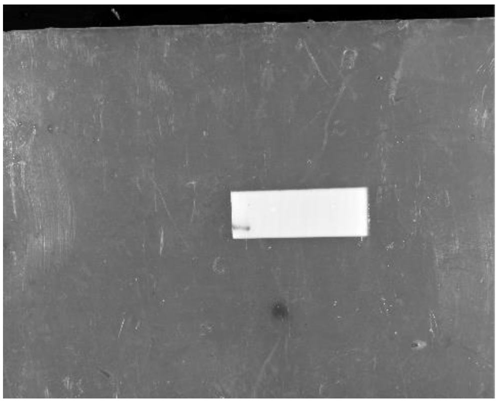

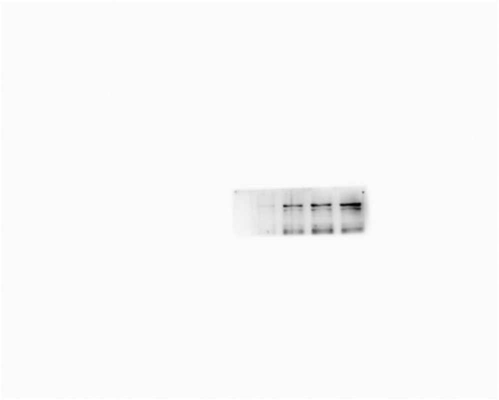

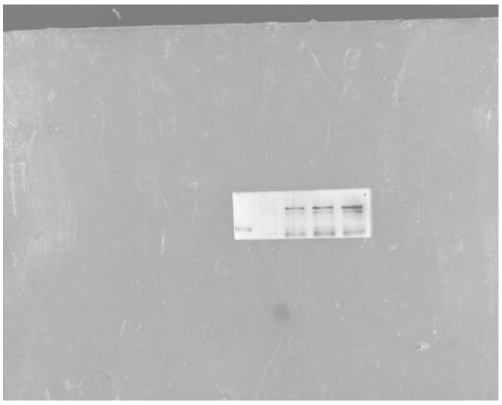

Sox2

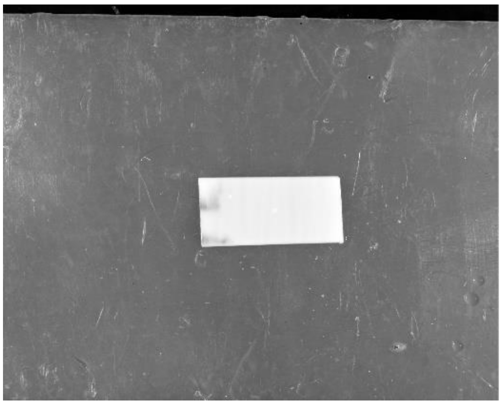

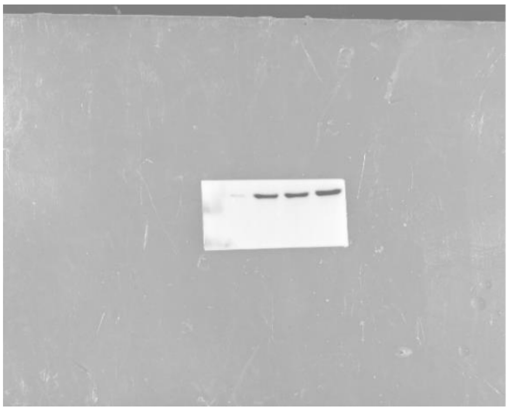

KLF4

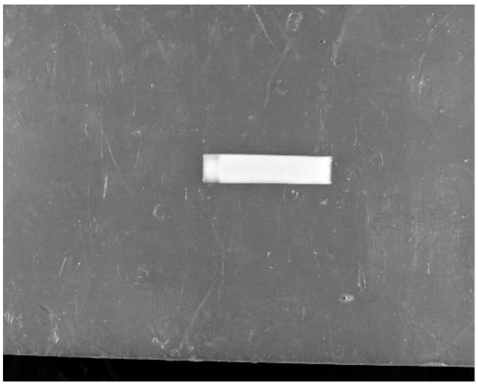

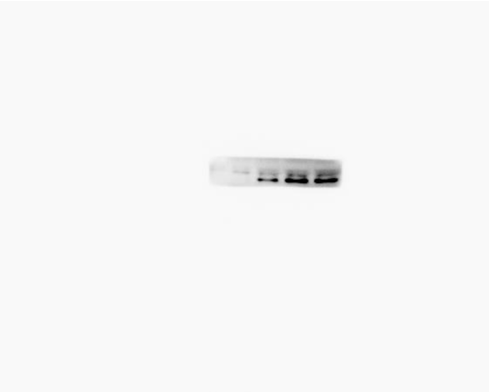

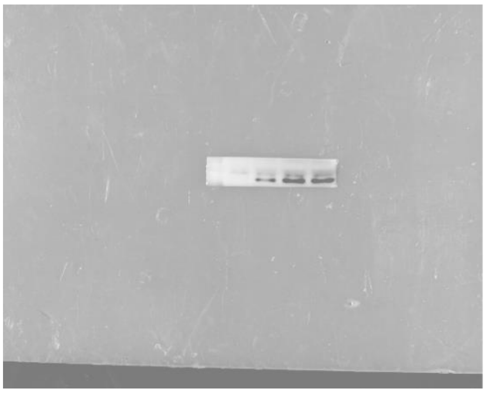

Actin

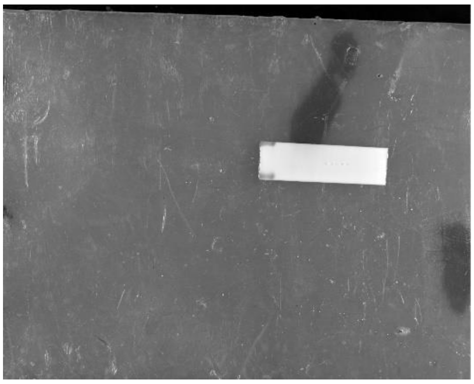

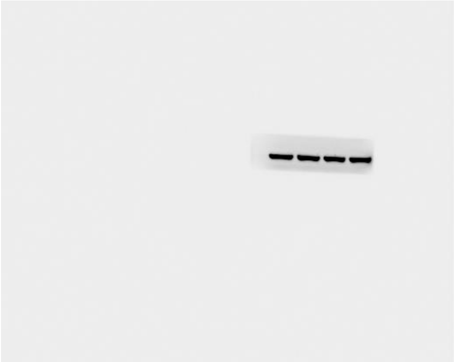

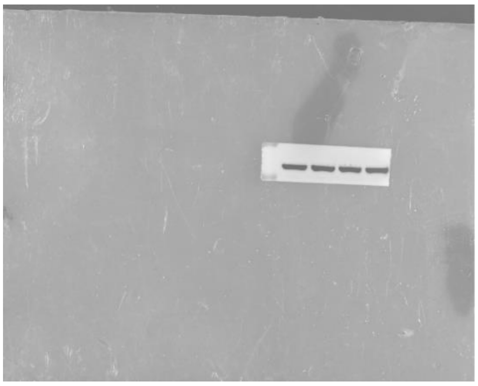

U118 Raw data of WB

CD133

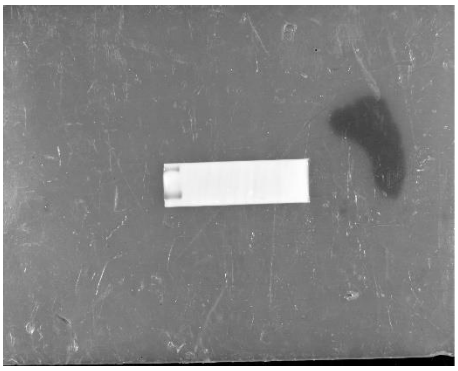

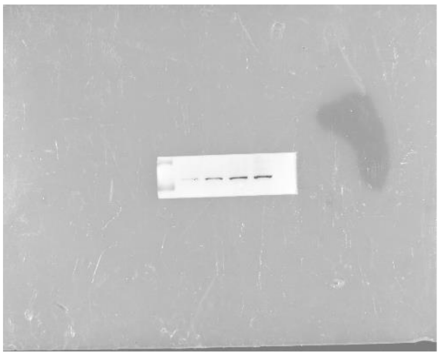

CD15

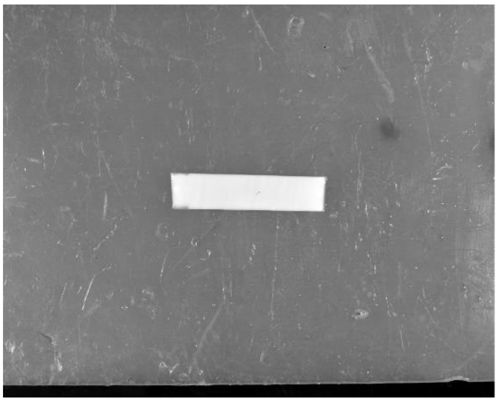

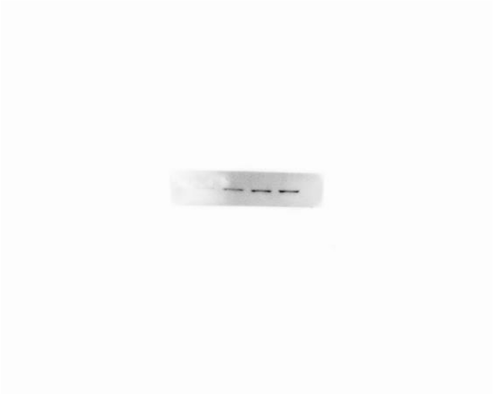

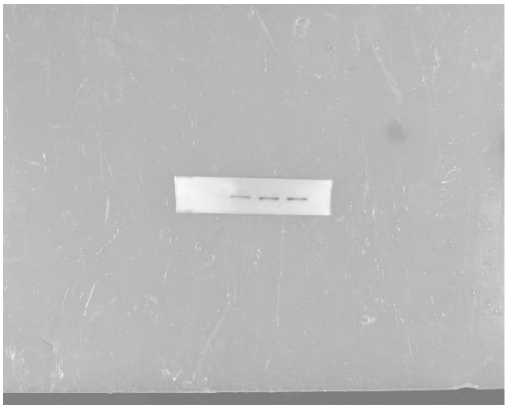

Nestin

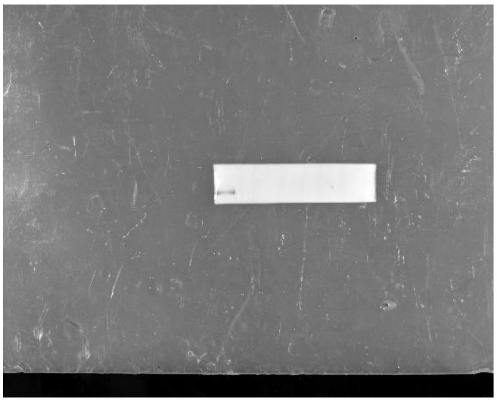

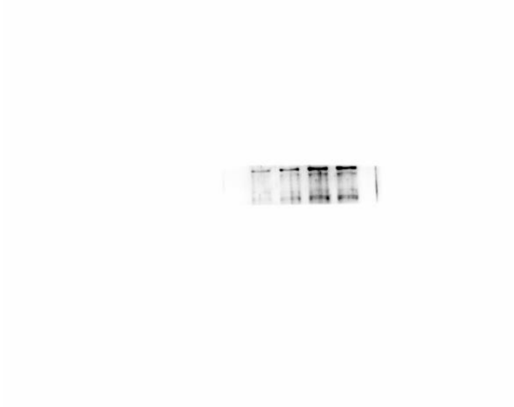

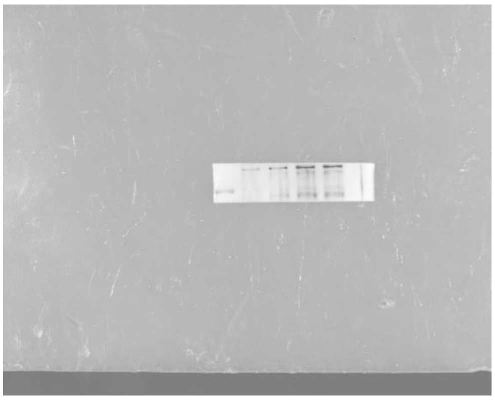

Sox2

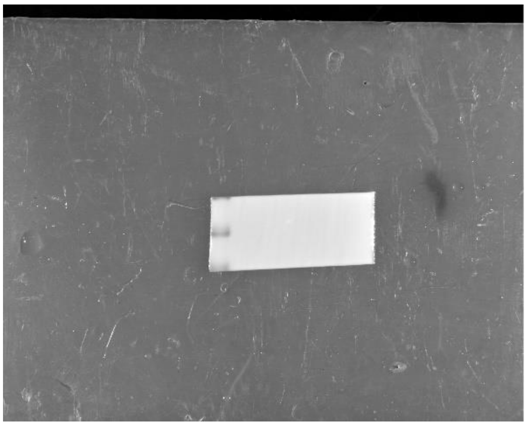

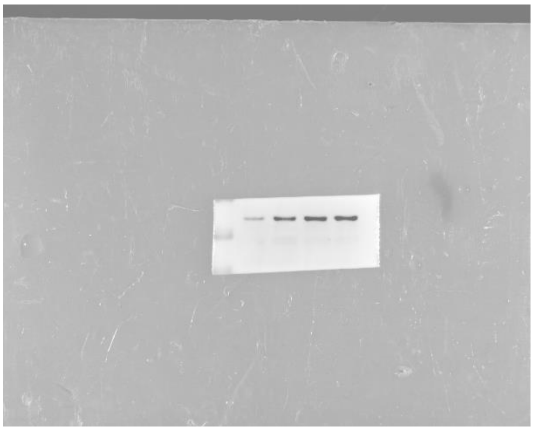

Klf4

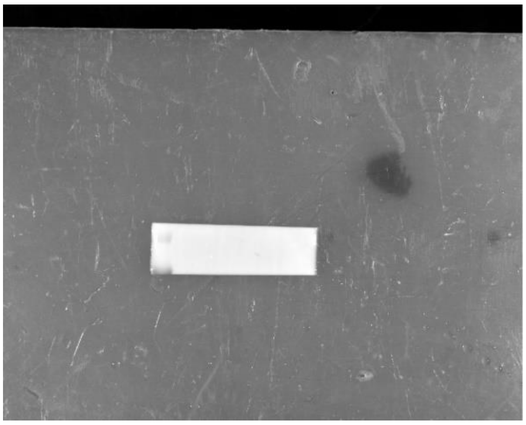

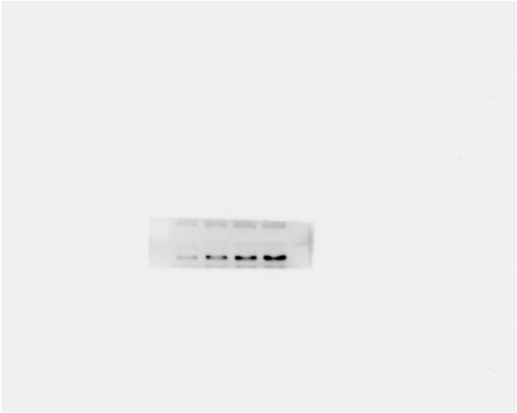

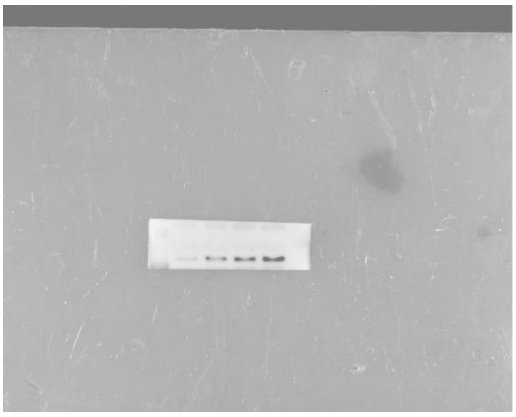

ACTIN

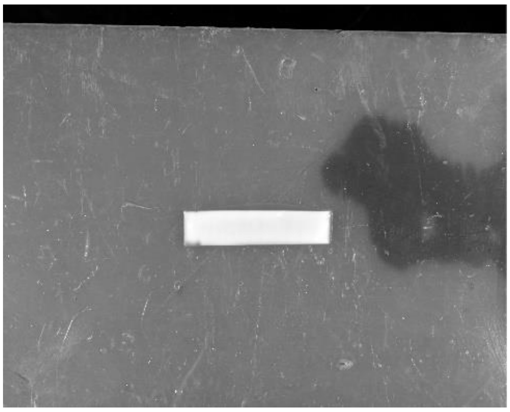

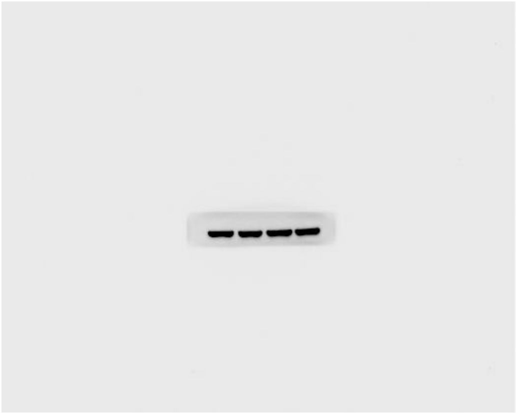

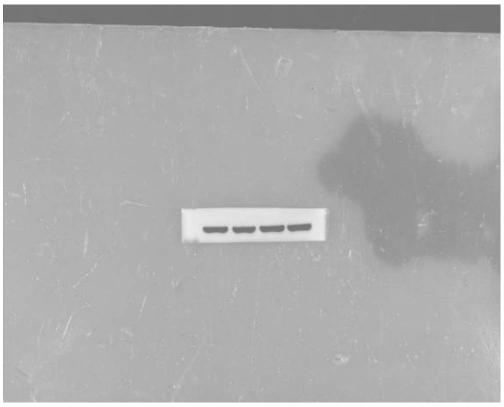

Figure 6 WB Raw data

CD15

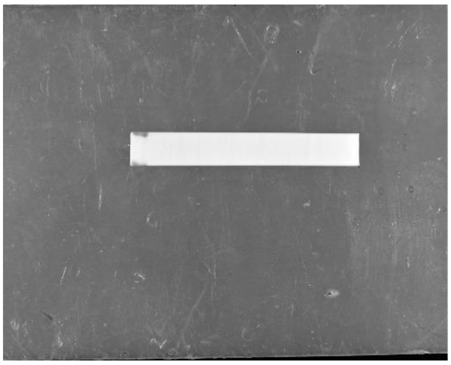

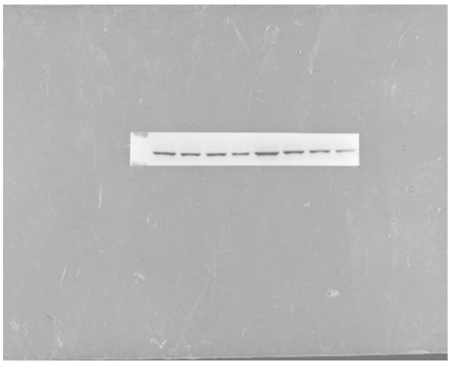

CD133

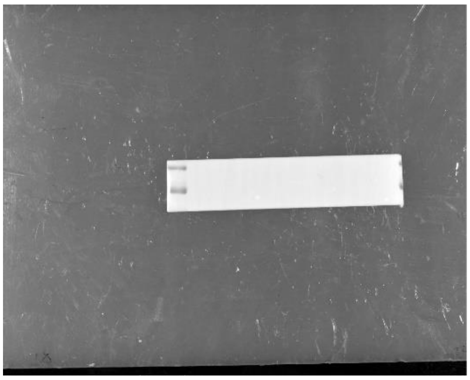

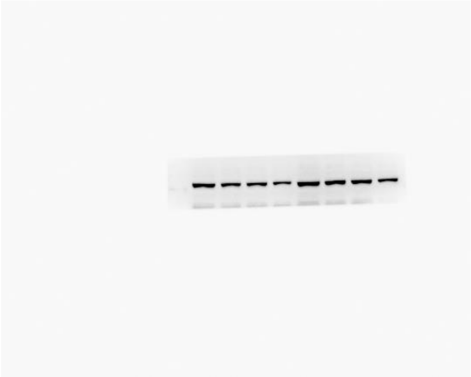

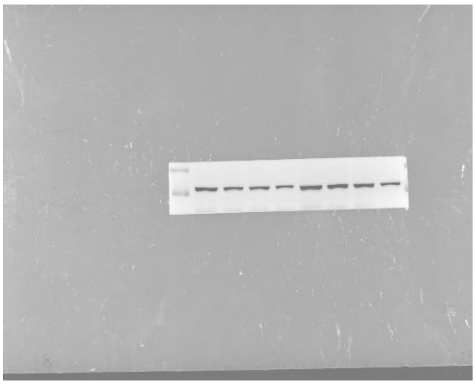

Nestin

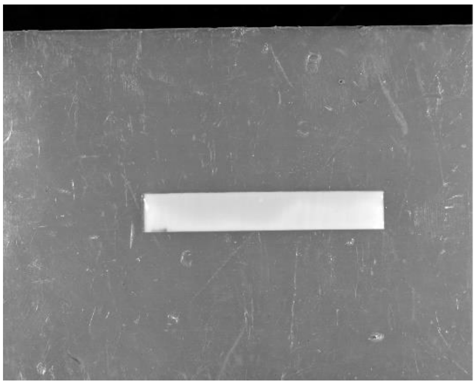

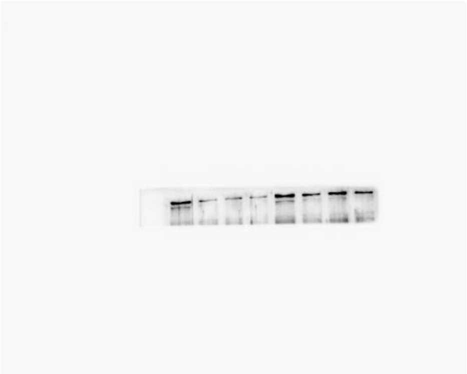

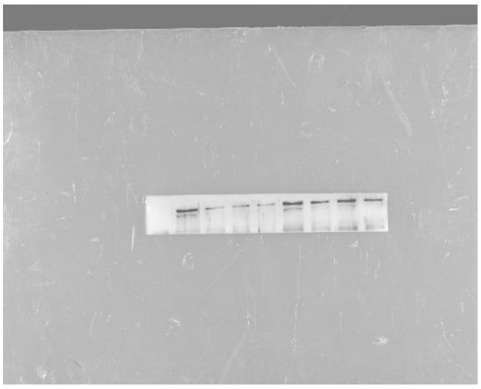

Sox2

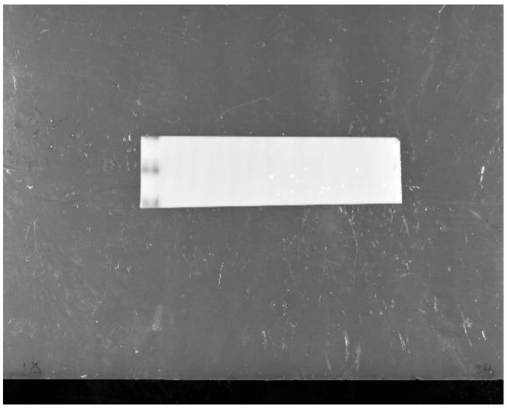

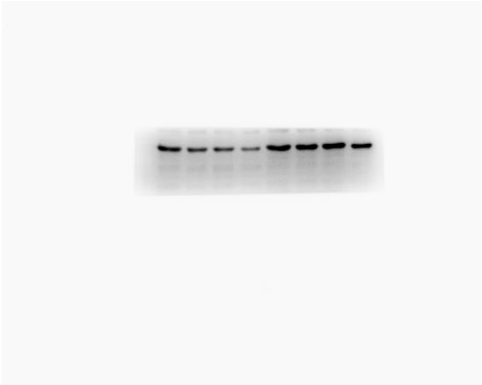

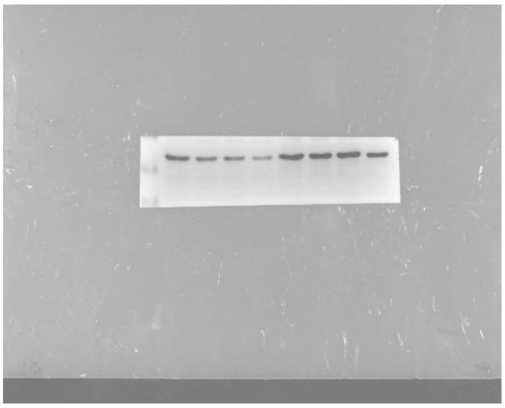

KLF4

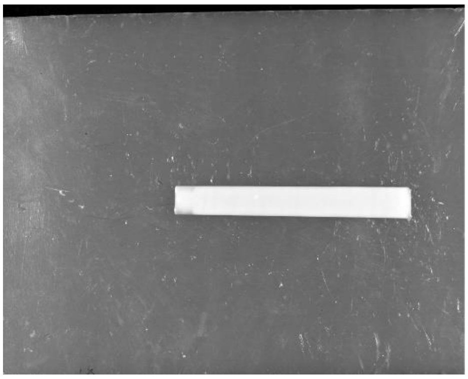

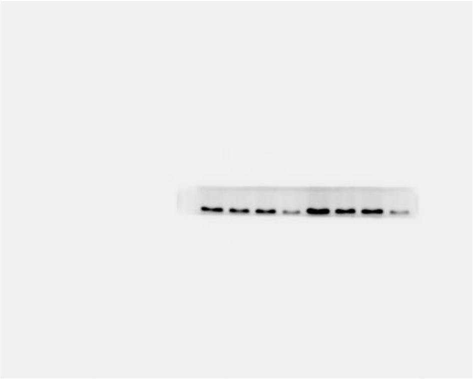

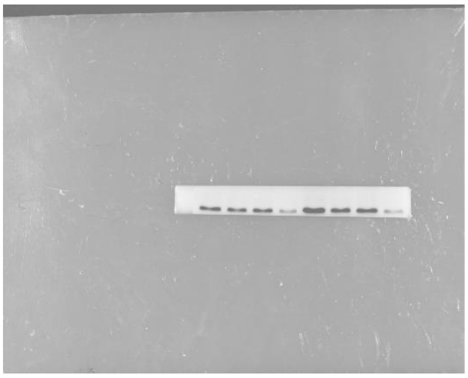

Actin

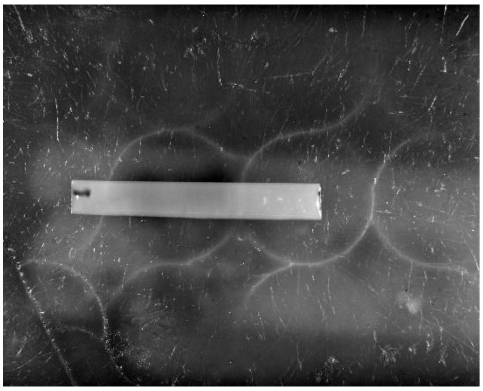

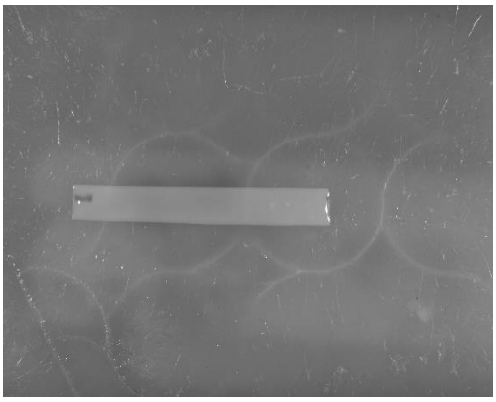

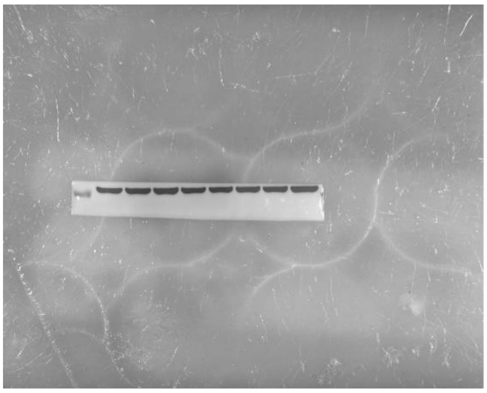

Sup 6 WB Raw data

CD133

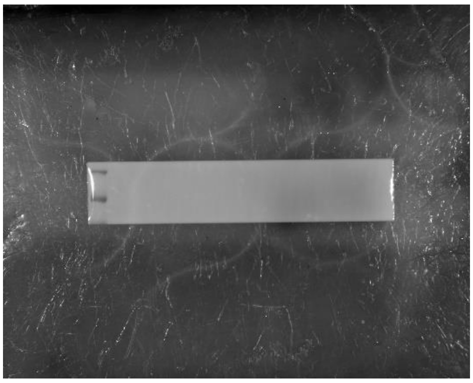

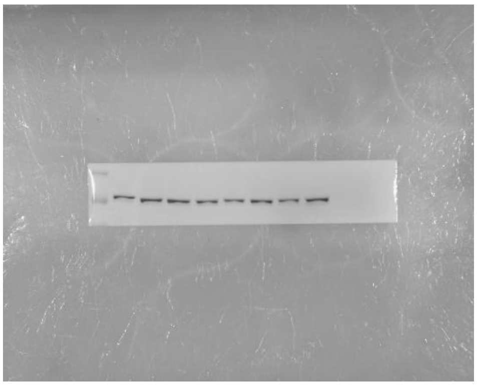

CD15

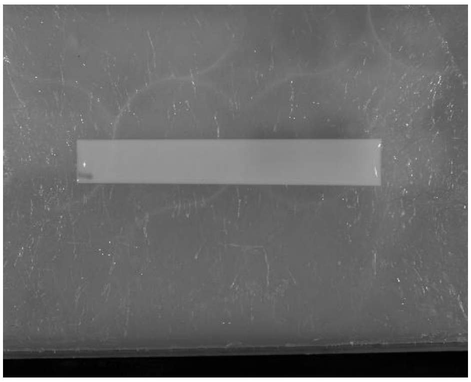

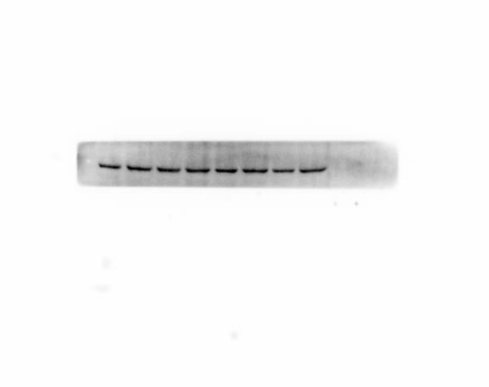

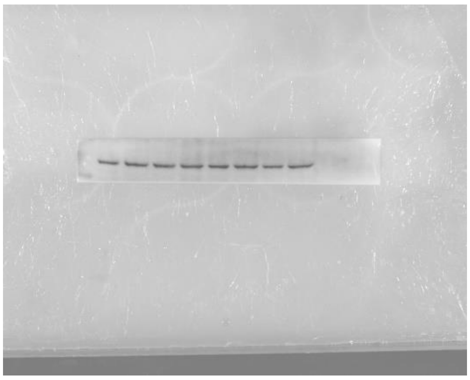

Nestin

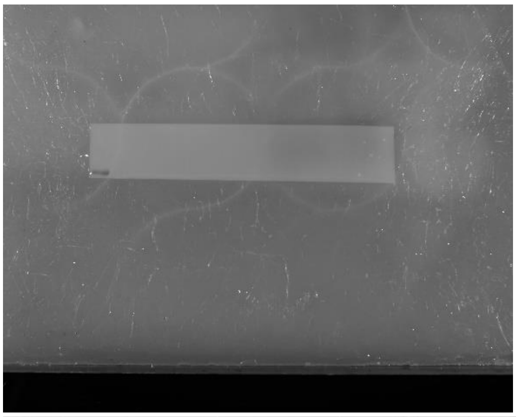

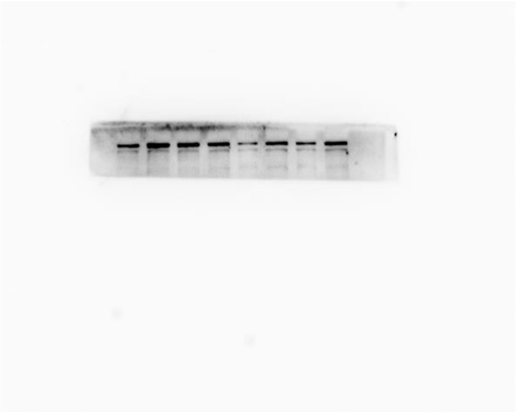

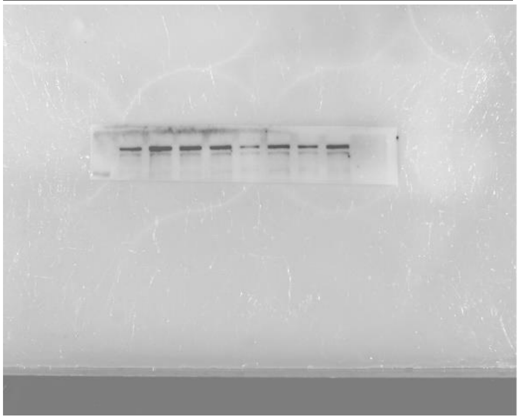

Actin

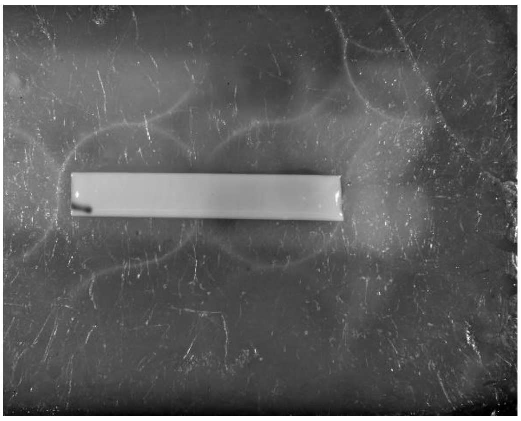

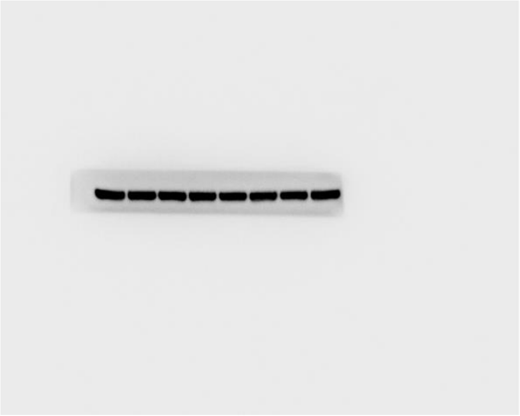

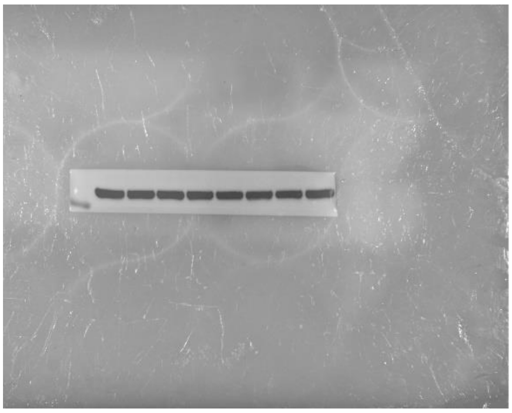

Sox2

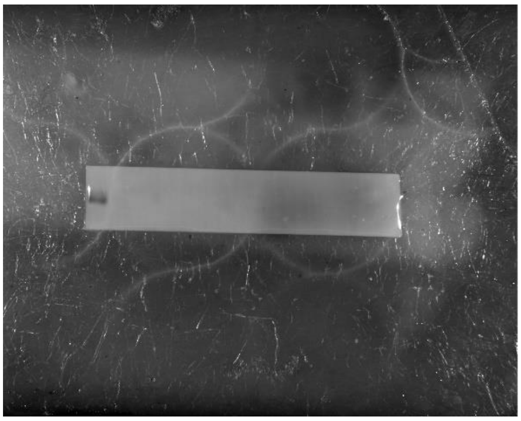

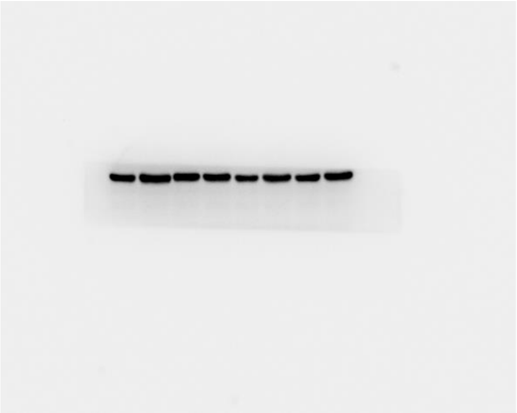

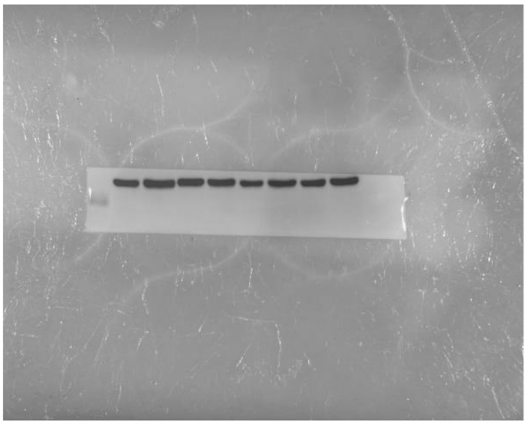

KLF4

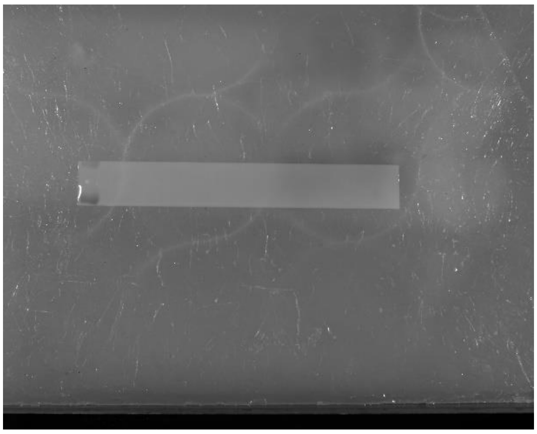

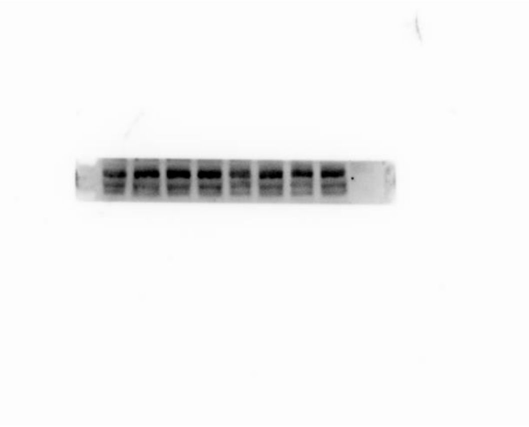

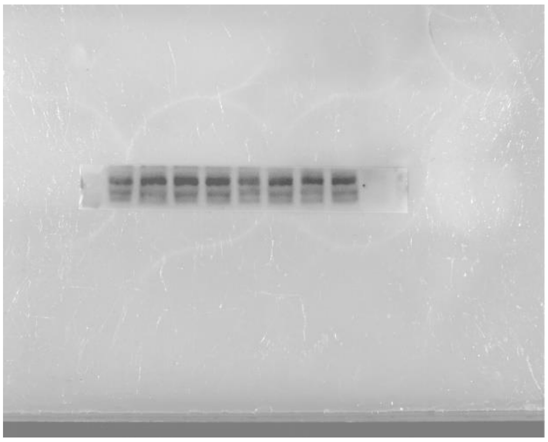

GAPDH

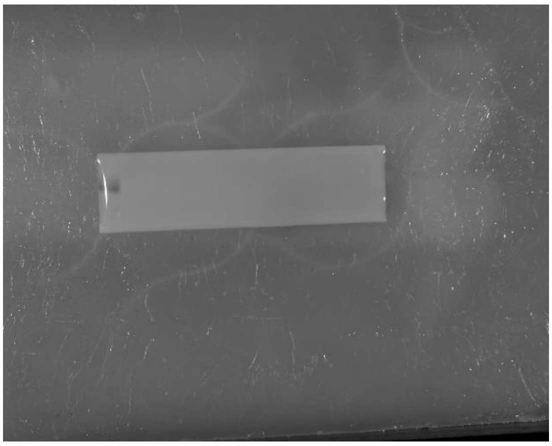

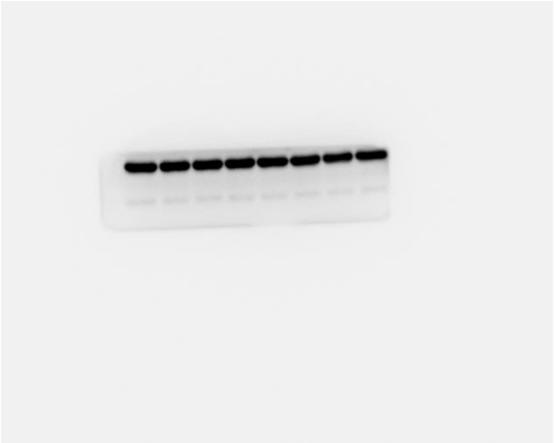

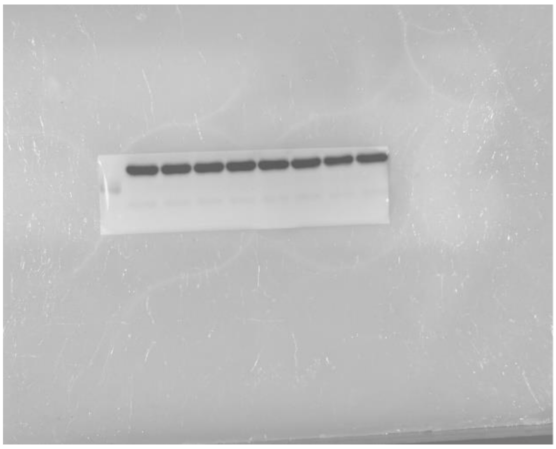

Sup 8 WB Raw data

CD133

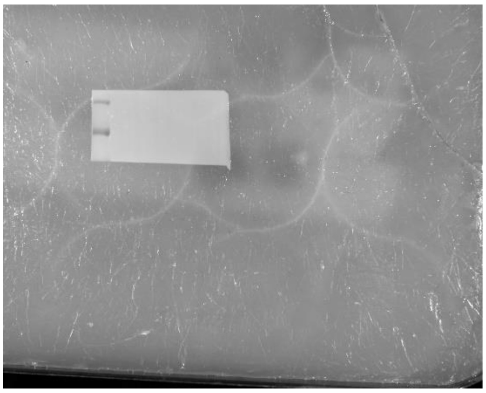

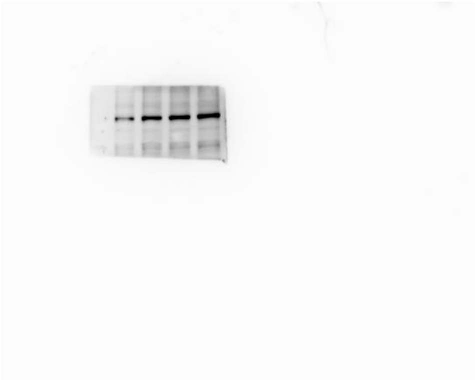

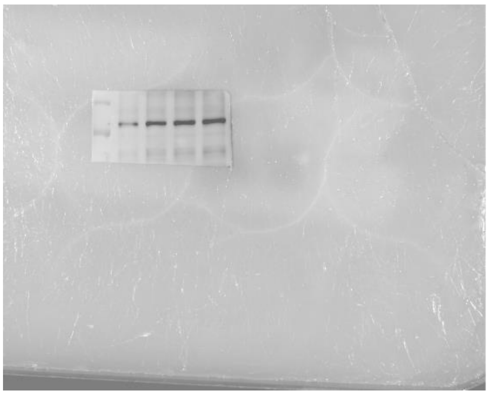

Nestin

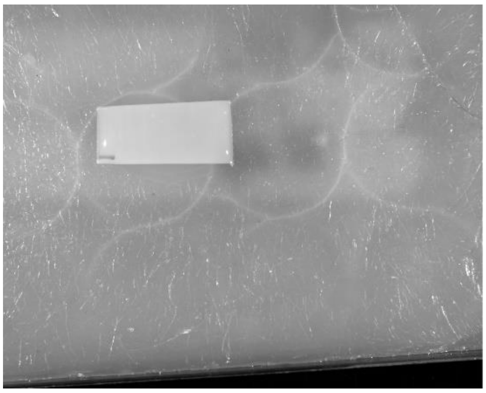

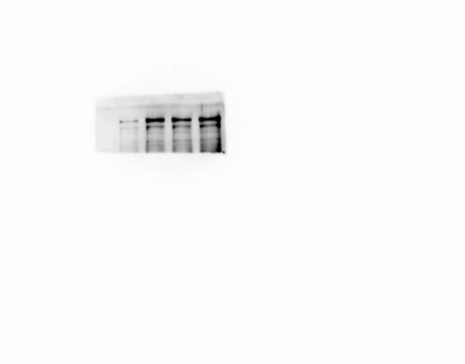

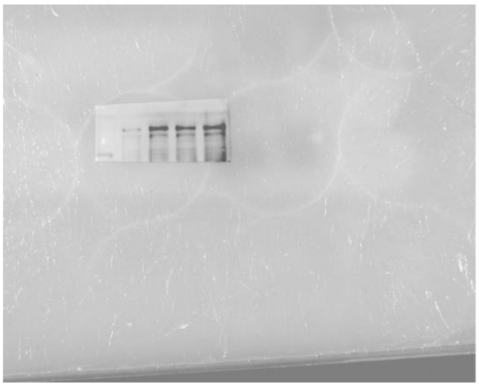

Sox2

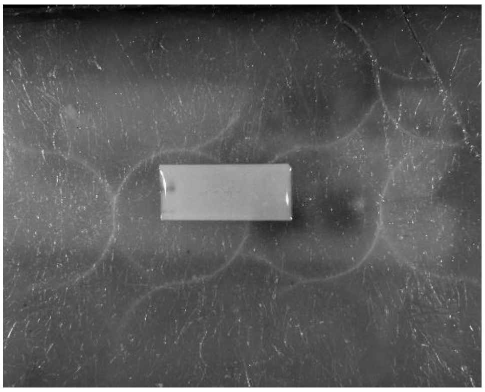

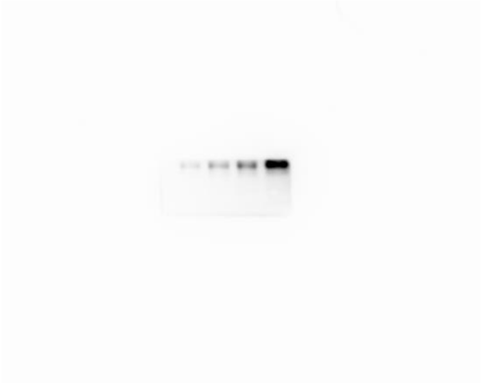

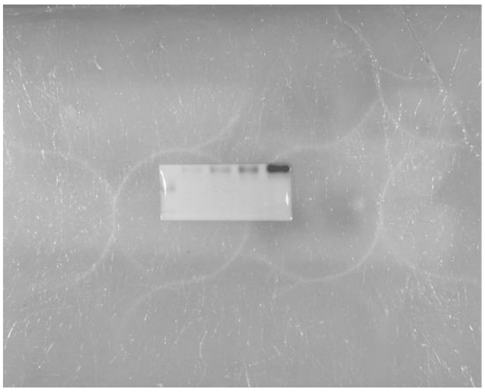

KLF4

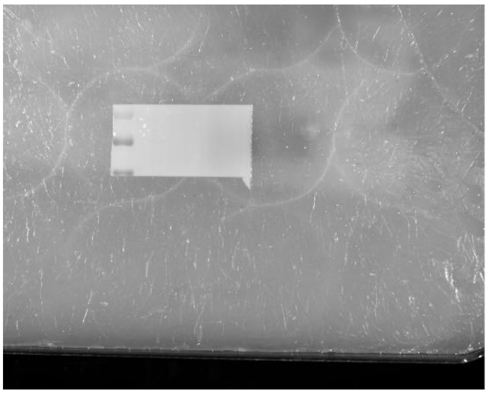

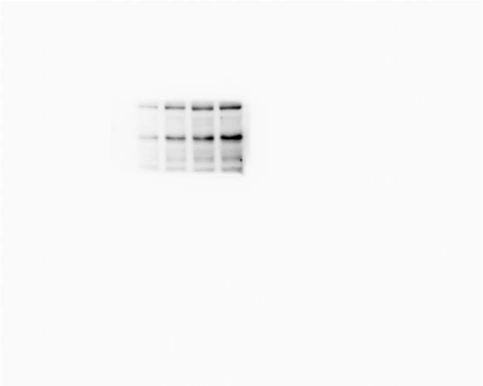

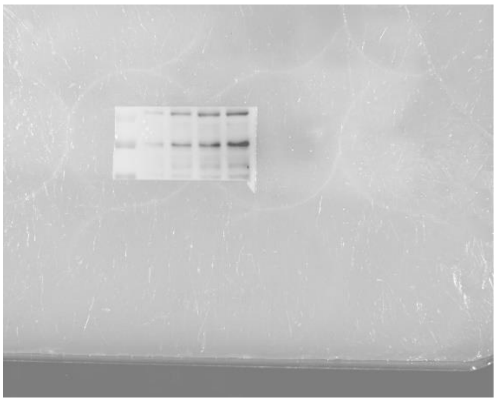

Actin

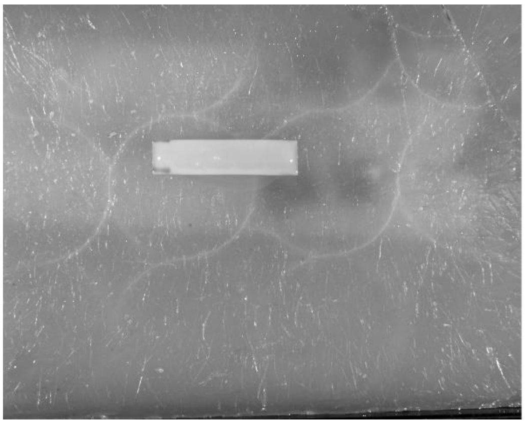

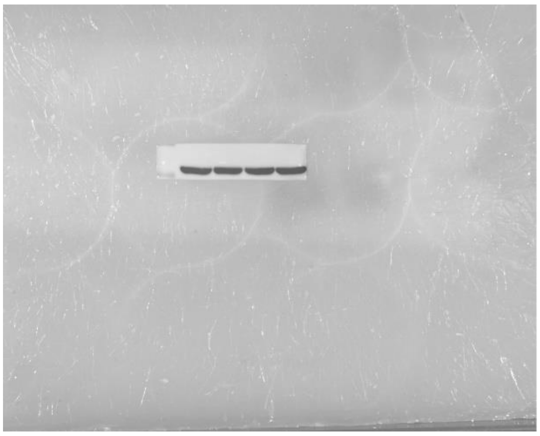

Sup 15 WB Raw data

B

U87-HIF1a

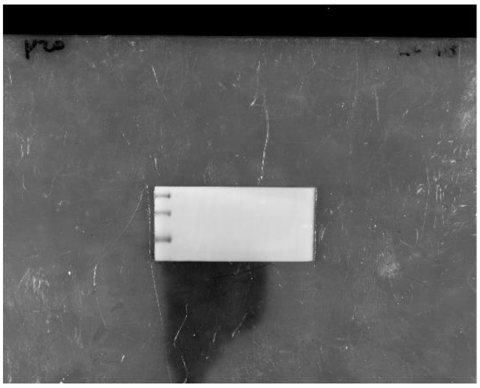

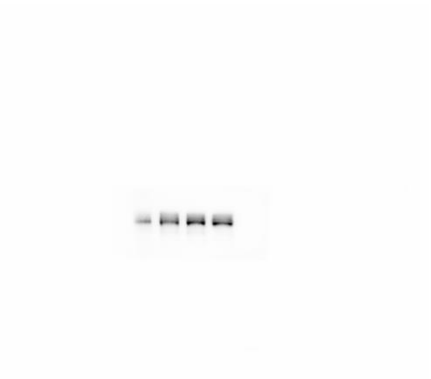

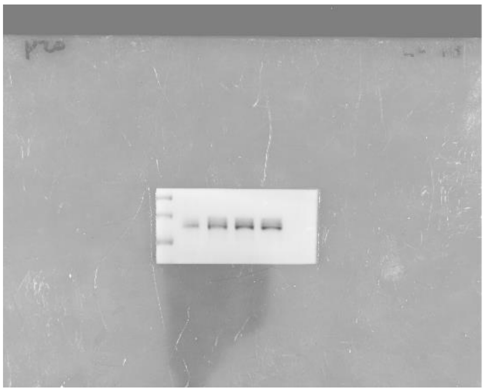

U87-HIF2a

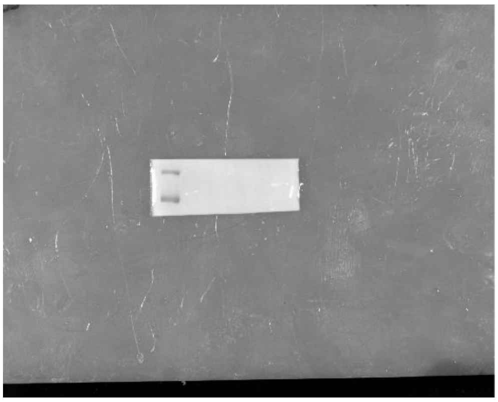

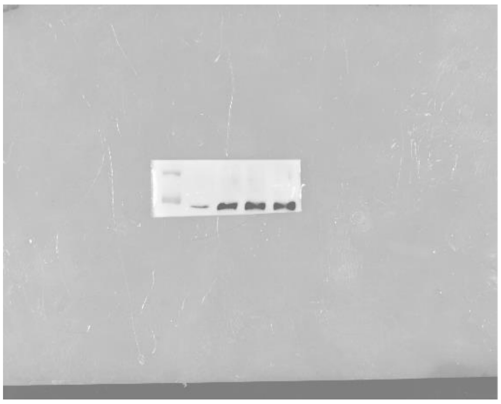

U87-actin

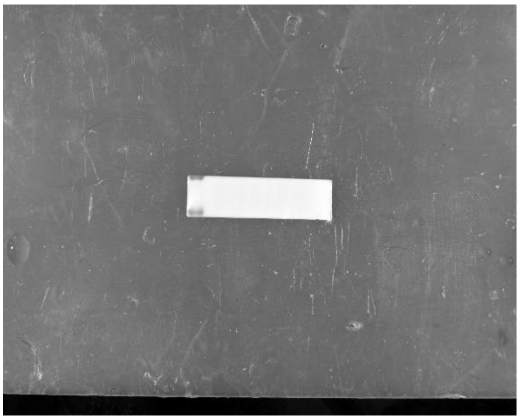

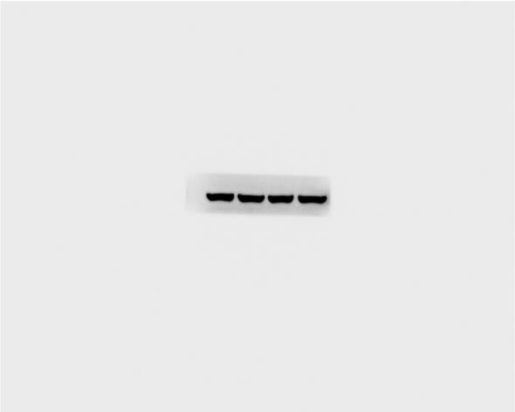

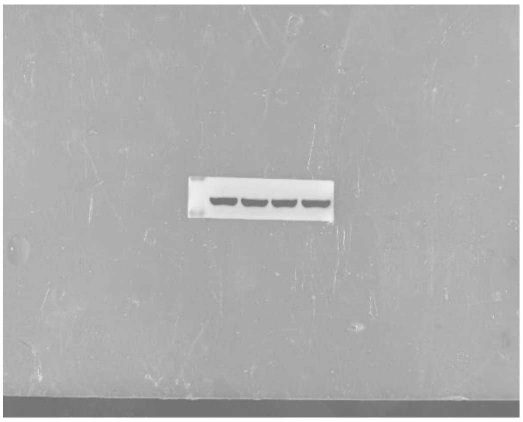

U118-HIF1a

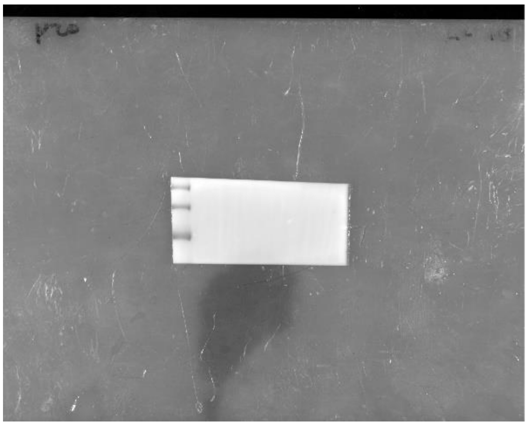

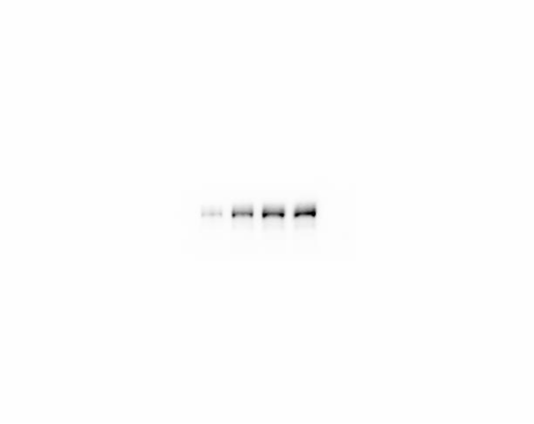

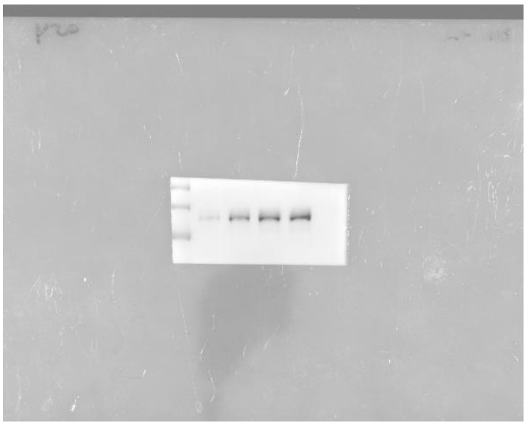

U118-HIF2a

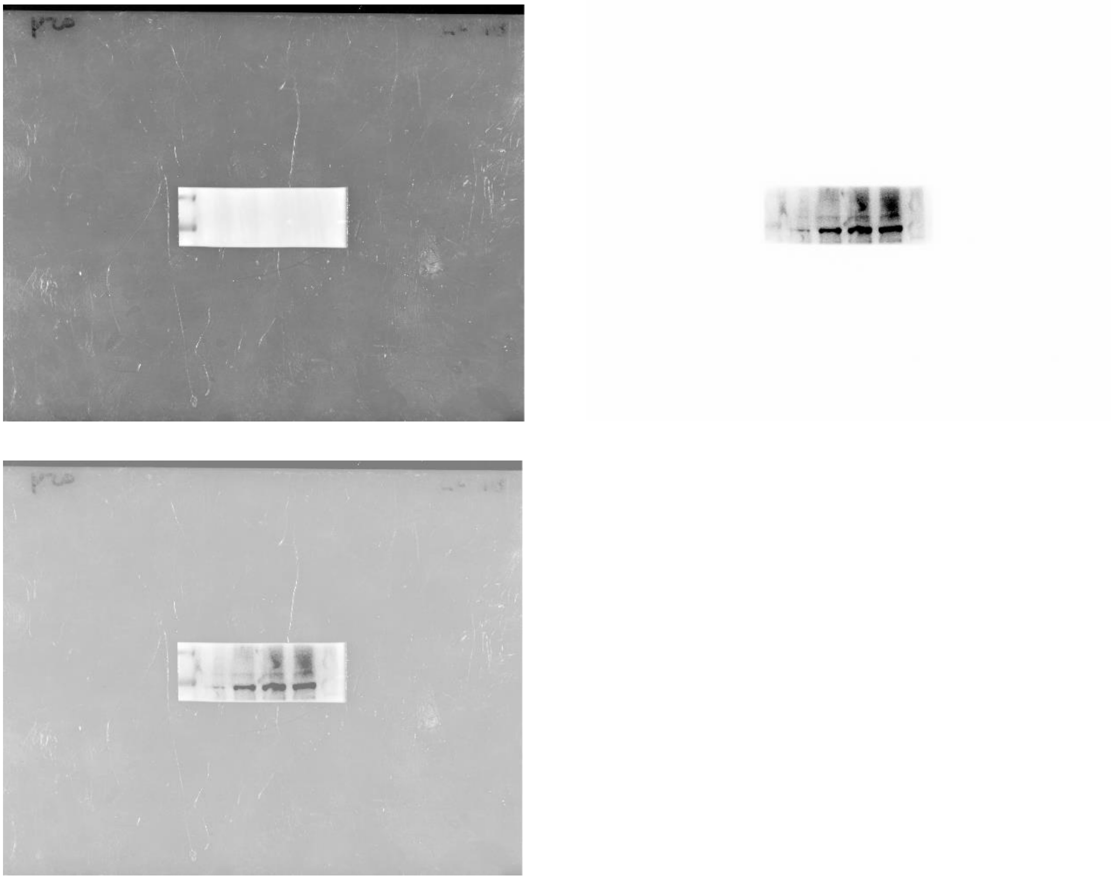

U118-GAPDH

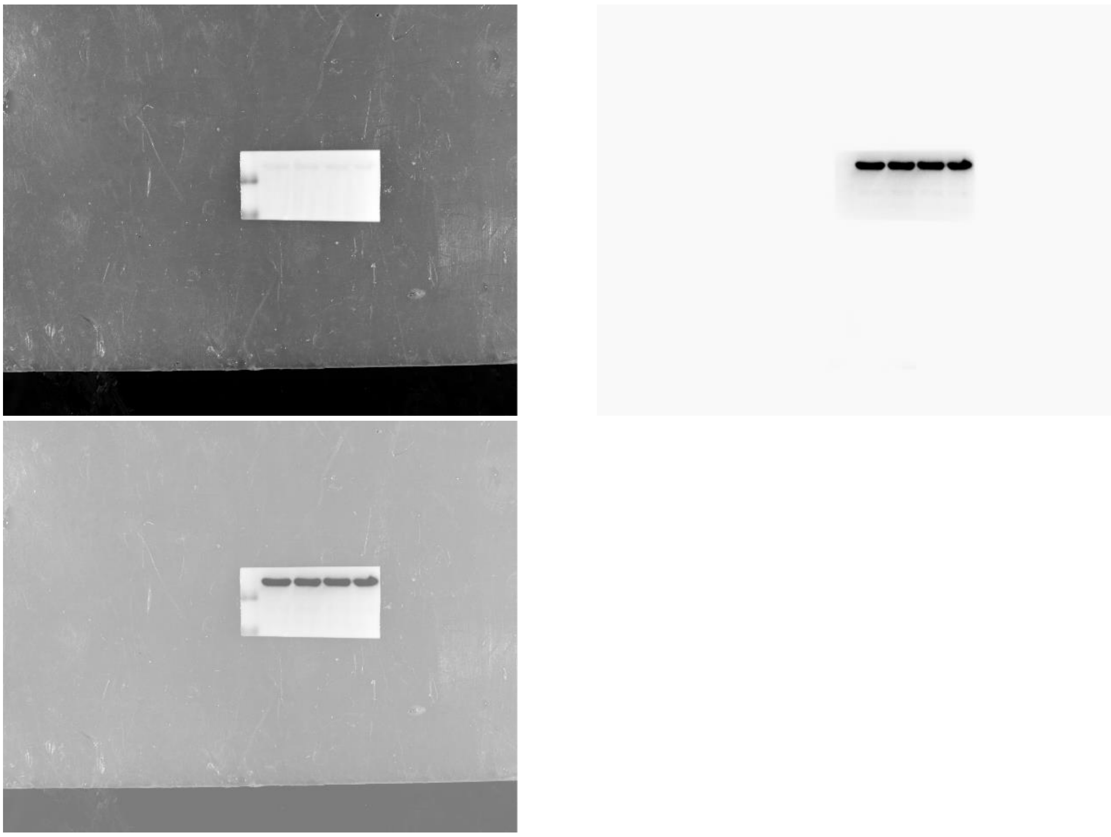

C

HIF1a

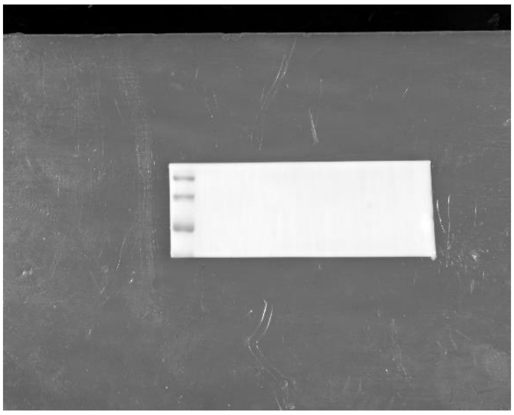

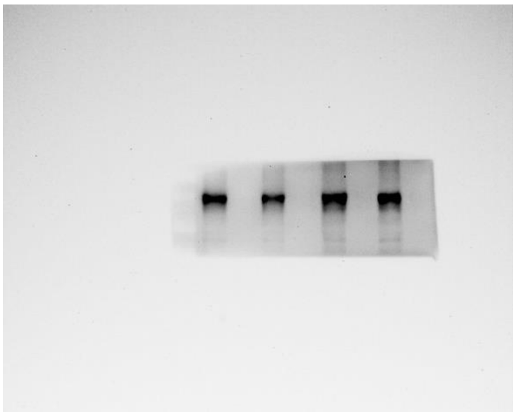

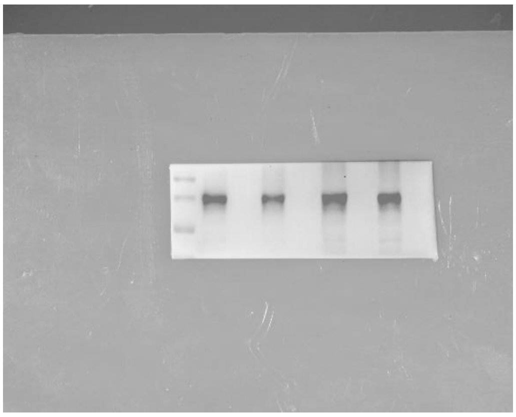

HIF2a

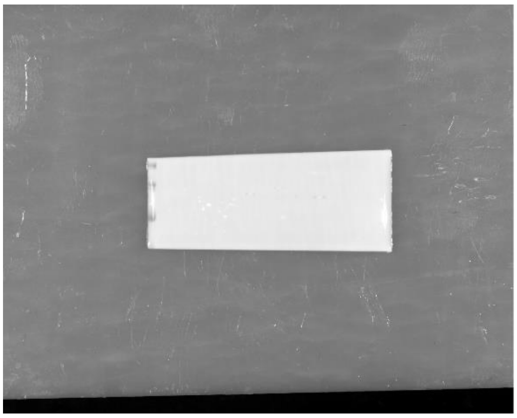

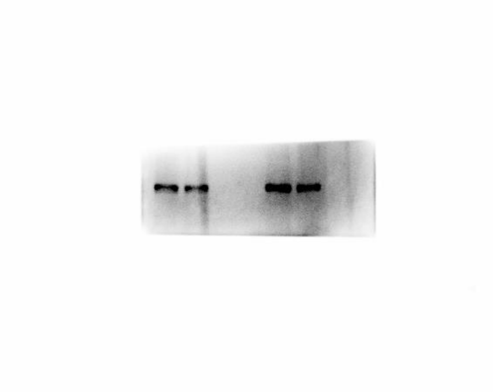

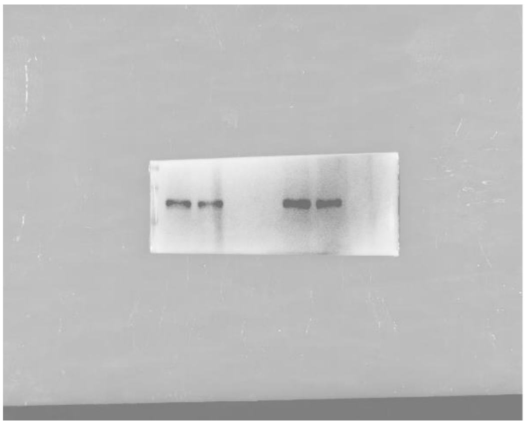

Actin

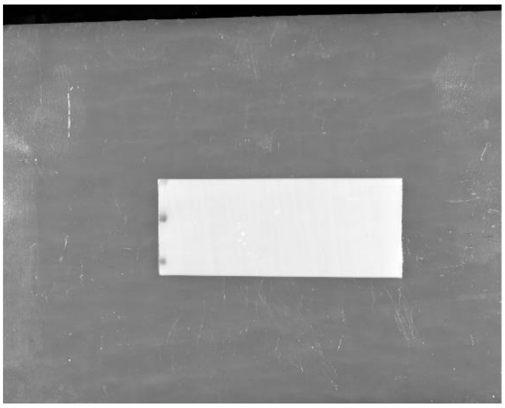

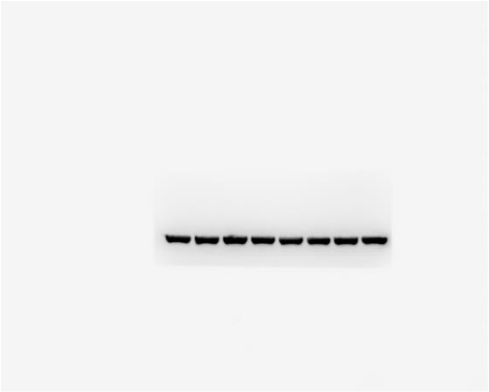

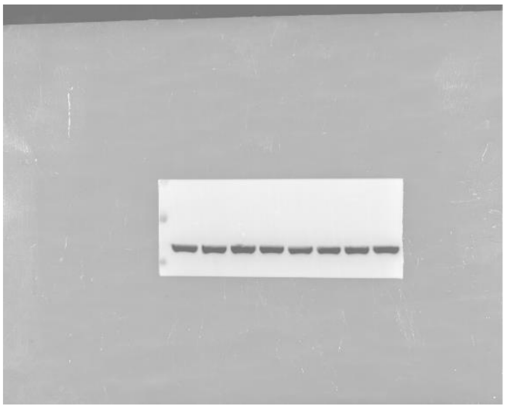

Sup 16 WB Raw data

CD133

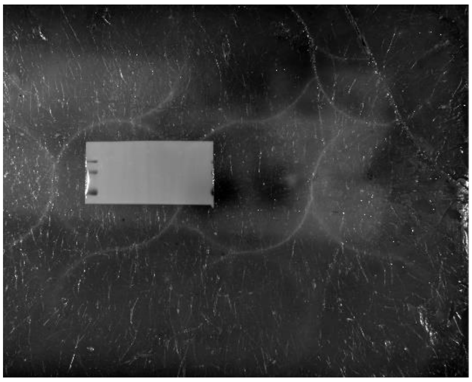

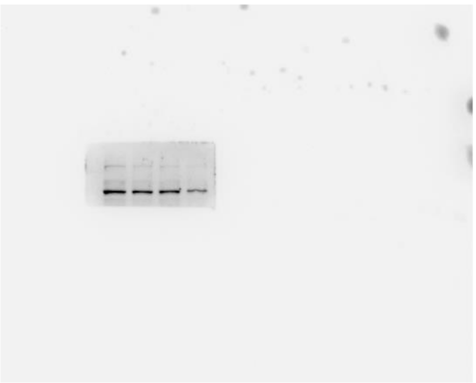

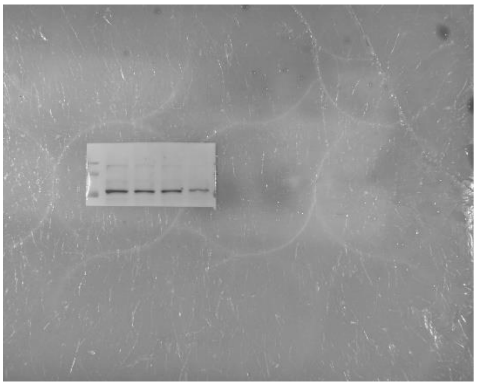

Nestin

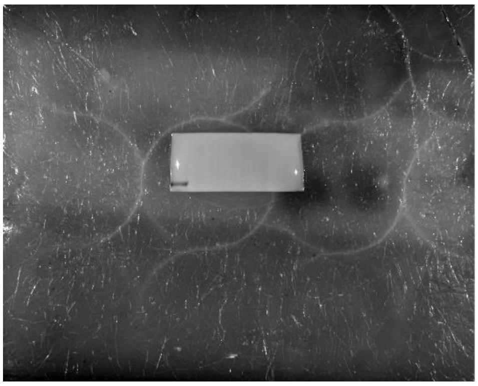

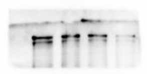

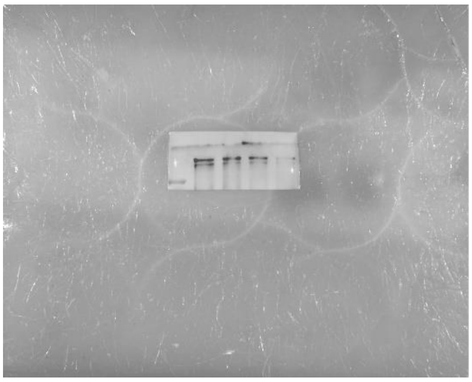

Sox2

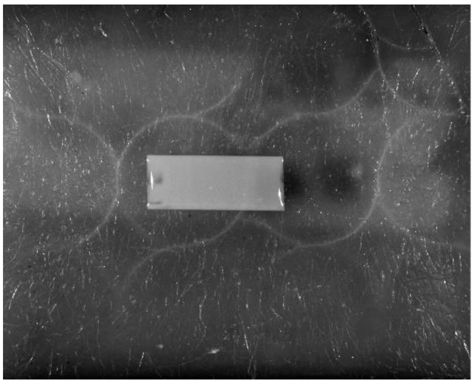

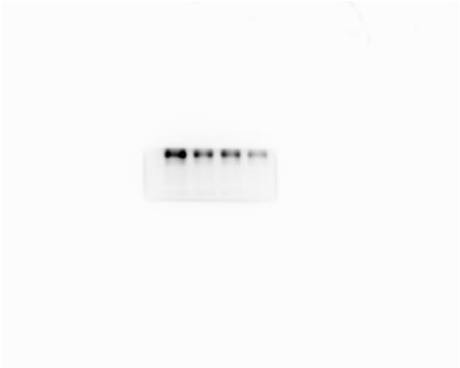

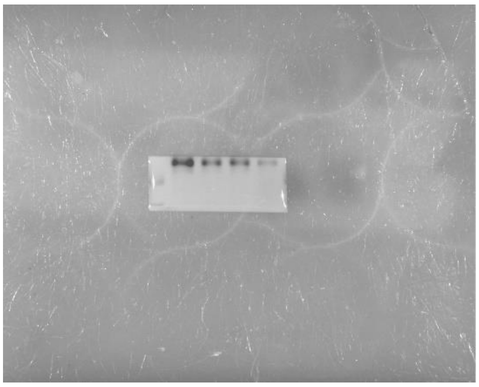

KLF4

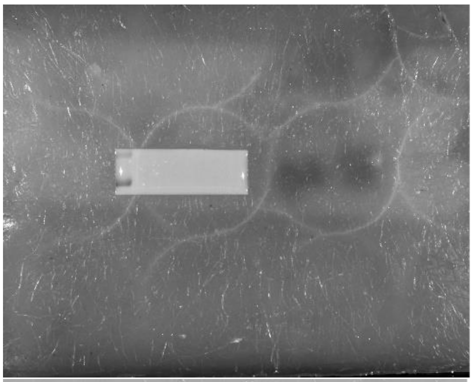

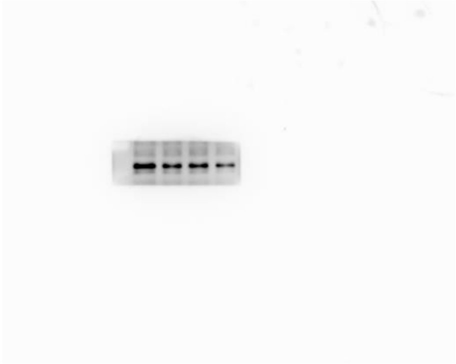

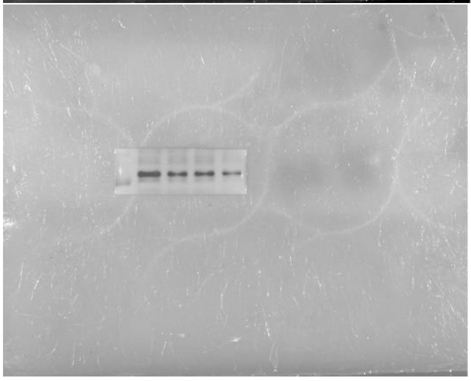

HIF1a

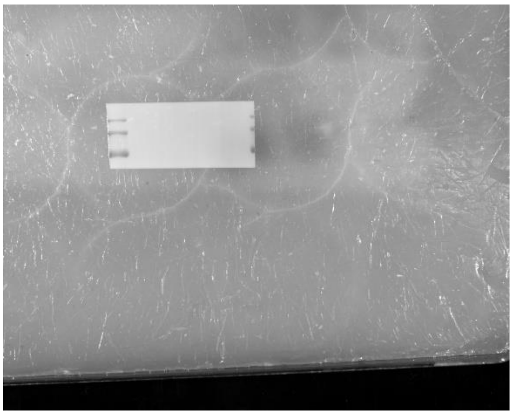

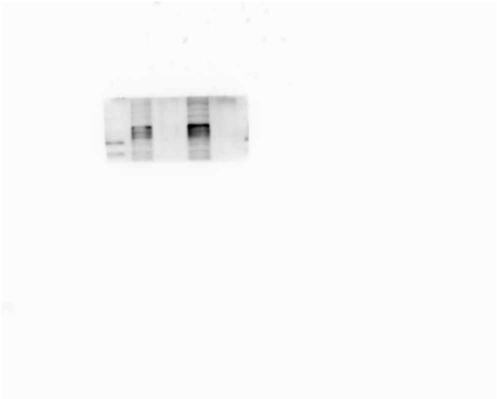

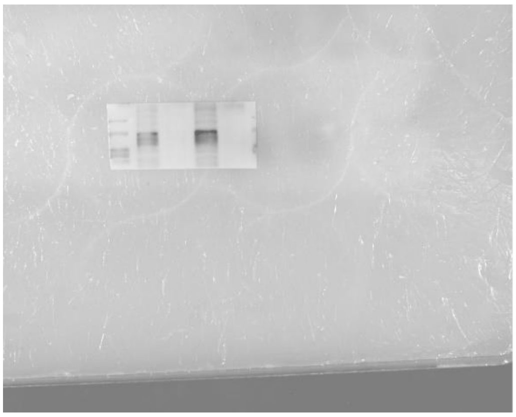

HIF2a

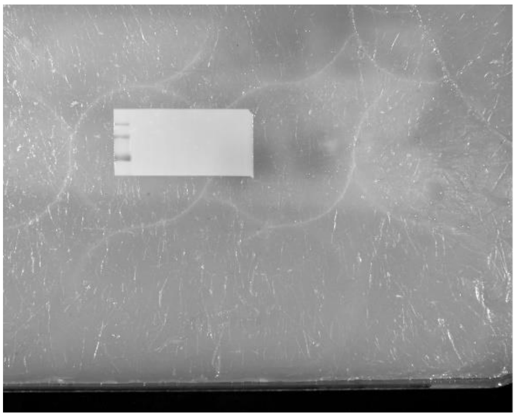

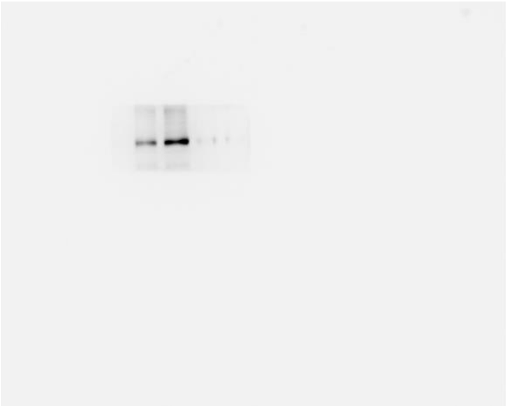

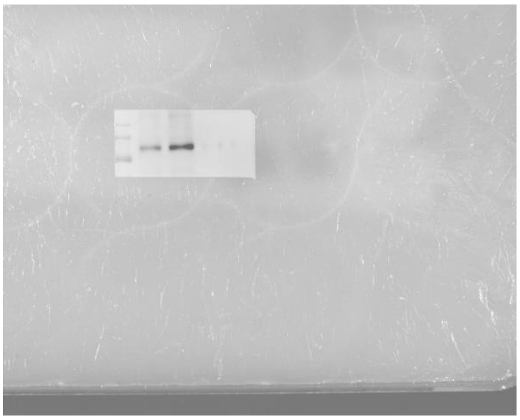

Actin

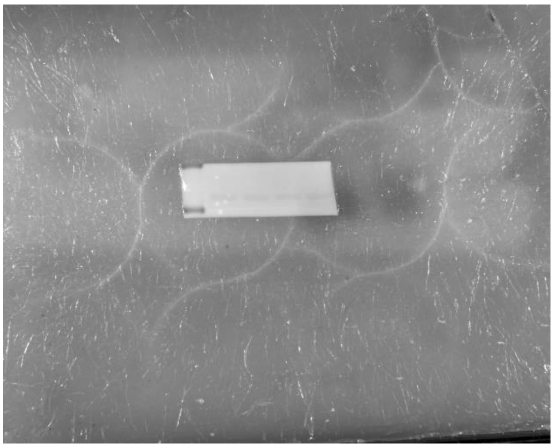

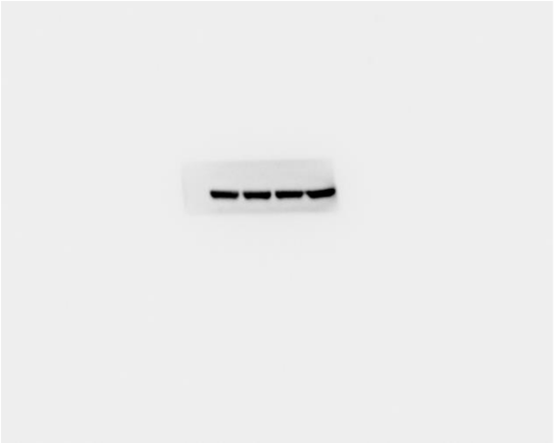

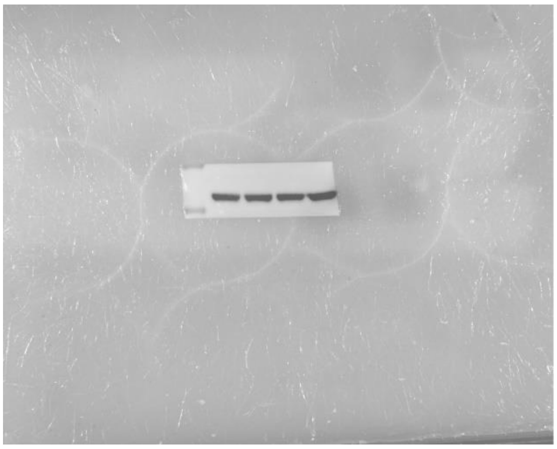

Supplement: Supplementary file 12 — Original Data [file 41419_2025_7617_MOESM12_ESM.pdf]
